# DrivAER: Identification of driving transcriptional programs in single-cell RNA sequencing data

**DOI:** 10.1093/gigascience/giaa122

**Published:** 2020-12-10

**Authors:** Lukas M Simon, Fangfang Yan, Zhongming Zhao

**Affiliations:** Center for Precision Health, School of Biomedical Informatics, The University of Texas Health Science Center at Houston, 7000 Fannin St, Houston, TX 77030, USA; Center for Precision Health, School of Biomedical Informatics, The University of Texas Health Science Center at Houston, 7000 Fannin St, Houston, TX 77030, USA; Center for Precision Health, School of Biomedical Informatics, The University of Texas Health Science Center at Houston, 7000 Fannin St, Houston, TX 77030, USA; Human Genetics Center, School of Public Health, The University of Texas Health Science Center at Houston, 7000 Fannin St, Houston, TX 77030, USA; MD Anderson Cancer Center UTHealth Graduate School of Biomedical Sciences, 6767 Bertner Ave, Houston, TX 77030, USA; Department of Biomedical Informatics, Vanderbilt University Medical Center, 2525 West End, Nashville, TN 37203, USA

**Keywords:** Autoencoder, machine learning, manifold interpretation, single-cell RNA sequencing, transcription factor

## Abstract

**Background:**

Single-cell RNA sequencing (scRNA-seq) unfolds complex transcriptomic datasets into detailed cellular maps. Despite recent success, there is a pressing need for specialized methods tailored towards the functional interpretation of these cellular maps.

**Findings:**

Here, we present DrivAER, a machine learning approach for the identification of driving transcriptional programs using autoencoder-based relevance scores. DrivAER scores annotated gene sets on the basis of their relevance to user-specified outcomes such as pseudotemporal ordering or disease status. DrivAER iteratively evaluates the information content of each gene set with respect to the outcome variable using autoencoders. We benchmark our method using extensive simulation analysis as well as comparison to existing methods for functional interpretation of scRNA-seq data. Furthermore, we demonstrate that DrivAER extracts key pathways and transcription factors that regulate complex biological processes from scRNA-seq data.

**Conclusions:**

By quantifying the relevance of annotated gene sets with respect to specified outcome variables, DrivAER greatly enhances our ability to understand the underlying molecular mechanisms.

## Findings

### Background

Single-cell RNA sequencing (scRNA-seq) experiments dissect biological processes or complex tissues at the cellular and molecular levels [1, 2]. Owing to the high complexity and large number of observations, 1 critical step in scRNA-seq analysis is dimension reduction [3]. Dimension reduction projects the high-dimensional expression matrix into a low-dimensional space, also called data manifold or cellular map, which captures the underlying biological processes [4]. A number of methods have been used for manifold learning in scRNA-seq data [5–10].

Biological meaning can be extracted from the data manifold following in-depth analysis. After cells are stratified into separate groups or along a continuum, differential expression analysis is performed. Gene set enrichment analysis represents one of the most popular approaches to interpreting lists of differentially expressed (DE) genes and has been frequently used on bulk RNA-seq data [11–13]. More recent work has adapted this approach to scRNA-seq data [14]. Additional tools for biological interpretation of scRNA-seq data focus on the identification of latent variation that is aligned with gene sets [15–18].

However, choosing the best parameters to identify DE genes across diverse scRNA-seq datasets is still an open challenge [19]. Moreover, subtle transcriptional signals driven by a specific set of genes may not be sufficiently reflected in the global data manifold. Therefore, there is a need for methods that facilitate biological interpretation without performing differential expression analysis that capture subtle transcriptional signals driven by knowledge-based annotated gene sets.

Here, we present DrivAER, a method for the identification of driving transcriptional programs based on autoencoder-derived relevance scores. Transcriptional programs (TPs) are sets of genes sharing biological properties [20] such as genes sharing transcription factor (TF) binding motifs or genes involved in the same biological pathway [11, 21]. TPs have been annotated extensively, and DrivAER infers TP relevance scores for existing gene set annotations with respect to specified outcomes of interest. These outcomes can represent extrinsic phenotypes, such as disease status, or intrinsic phenotypes derived from the data itself, such as pseudotemporal trajectories. Relevance scores allow researchers to rank TPs and help explain the underlying molecular mechanisms.

We evaluated DrivAER by application to 2 publicly available scRNA-seq datasets and comparison to 2 competing methods called VISION [22] and PAGODA [15]. Our results demonstrate that DrivAER correctly extracts well-known regulators from complex scRNA-seq datasets profiling interferon stimulation and blood development. Moreover, DrivAER outperforms existing methods when subtle transcriptional signals are present. Our user-friendly tool integrates smoothly downstream of the popular scRNA-seq analysis framework Scanpy [23].

## Results

### DrivAER correctly identifies interferon response

**Figure 1:**
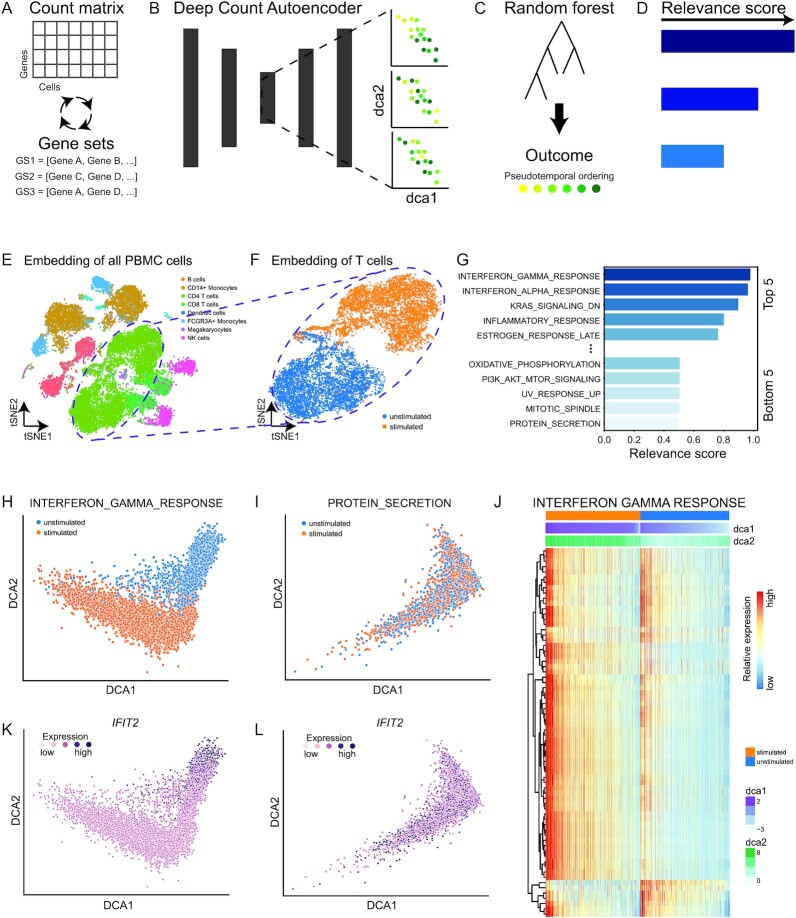
DrivAER correctly identifies interferon response. (A) DrivAER iteratively subjects annotated gene sets to unsupervised dimension reduction via Deep Count Autoencoder (DCA). (B) For each gene set, the 2D data manifold coordinates are calculated and (C) subsequently used as input features in a random forest model to predict the outcome of interest (i.e., pseudotemporal ordering). (D) The random forest prediction accuracy represents the relevance score. (E) t-Distributed stochastic neighbor embedding (tSNE) visualization displays all peripheral blood mononuclear cells (PBMCs) colored by cell type. NK: natural killer. (F) Cellular map (tSNE) of T cell subset clusters by stimulation status. (G) Bar plot indicates relevance scores of the 5 most and least relevant transcription programs. DCA embeddings calculated based on “INTERFERON_GAMMA_RESPONSE” (H) and “PROTEIN_SECRETION” (I) (negative control) gene sets are depicted. Cells are colored by stimulation status. (J) Heat map shows gene expression of “INTERFERON_GAMMA_RESPONSE” target genes and cells in rows and columns, respectively. Columns are ordered first by stimulation status and second by DCA coordinates. Bars on top of the heat map represent stimulation status and DCA coordinates 1 and 2. Red and blue colors correspond to high and low relative expression values. Relative expression of interferon gene *IFIT2* is overlaid on top of the DCA embeddings derived from “INTERFERON_GAMMA_RESPONSE” (K) and “PROTEIN_SECRETION” (L) gene sets. Dark colors indicate higher expression.

DrivAER is based on 1 assumption: the data manifold of relevant TPs shares information with the outcome of interest. Irrelevant TPs, on the other hand, will generate data manifolds where the cells fall randomly with respect to the outcome of interest. DrivAER builds upon our Deep Count Autoencoder (DCA) method [9], which has been shown to achieve high scalability for large scRNA-seq data [24]. DrivAER iteratively applies DCA to the raw counts of each annotated TP-specific gene set to generate a 2D data manifold in an unsupervised manner (Fig. 1A and B). Next, we associate the resulting manifold coordinates with the outcome of interest using random forest models (Fig. 1C). We interpret the random forest accuracy as relevance score, which quantifies the amount of information that is shared between the TP-specific data manifold and the outcome of interest (Fig. 1D).

To demonstrate the ability of DrivAER to perform correct manifold interpretation, we reanalyzed 2 publicly available scRNA-seq datasets. The first dataset by Kang et al. [25] described a transcriptional response to interferon stimulation (Fig. 1E). As a proof of principle, we asked whether DrivAER could recapitulate this biology and extract the interferon signature as the driving transcriptional program defining the T-cell data manifold (Fig. 1F). We applied DrivAER to the subset of T cells and evaluated all 50 hallmark gene sets from the Molecular Signatures Database (MolSigDB) [26] with respect to interferon stimulation (Supplementary Table S1). Indeed, the “INTERFERON_GAMMA_RESPONSE” gene set received the highest relevance score (Fig. 1G) among all 50 gene sets included in the analysis. Visualization of the T-cell DCA embedding derived from the “INTERFERON_GAMMA_RESPONSE” gene set showed clear separation by condition (Fig. 1H), implicating that this gene set is the main driving force separating the stimulated and unstimulated T cells. As a negative control, we show the DCA embedding for 1 of the lowest scoring gene sets “PROTEIN_SECRETION” (Fig. 1I). For this gene set, the cells cluster randomly with respect to the stimulation status. The heat map in Fig. 1J shows the expression levels of T cells for the “INTERFERON_GAMMA_RESPONSE” gene set. It is important to note that the cells (columns) are ordered by stimulation status and that the DCA coordinates are strongly associated with the stimulation status. Most “INTERFERON_GAMMA_RESPONSE” genes are upregulated in stimulated compared to unstimulated cells. Expression of genes in the “PROTEIN_SECRETION” gene set shows a random pattern (Supplementary Fig. S1).

To further manifest the biological meaning of the DCA embedding, we visualized the expression of interferon marker *IFIT2* in the “INTERFERON_GAMMA_RESPONSE” (Fig. 1K) and “PROTEIN_SECRETION” (Fig. 1L) embeddings. Expression levels of *IFIT2* increase along the DCA coordinates in the “INTERFERON_GAMMA_RESPONSE”-derived embedding. In contrast, *IFIT2* expression is distributed randomly in the “PROTEIN_SECRETION”-derived embedding. Therefore, DrivAER correctly identified the TPs driving interferon stimulation out of the entire collection of hallmark gene sets.

### DrivAER unveils key transcription factors in blood development

Next, we tested whether DrivAER is capable of extracting key TFs involved in differentiation trajectories. DrivAER is particularly well suited to infer the relevance of TFs for the following reasons. TF-mediated regulation is regarded as a combinatorial process that requires the coordination of multiple TFs and co-activators [27]. Moreover, there are vast differences in sensitivity and typical sequencing depth across various scRNA-seq technologies. Owing to the low RNA capture rate in some scRNA-seq technologies, generally TFs with low levels of expression may not be detected reliably [28]. Therefore, the expression levels of the target genes represent a better proxy of TF activity compared to the expression level of the TF itself [29].

**Figure 2:**
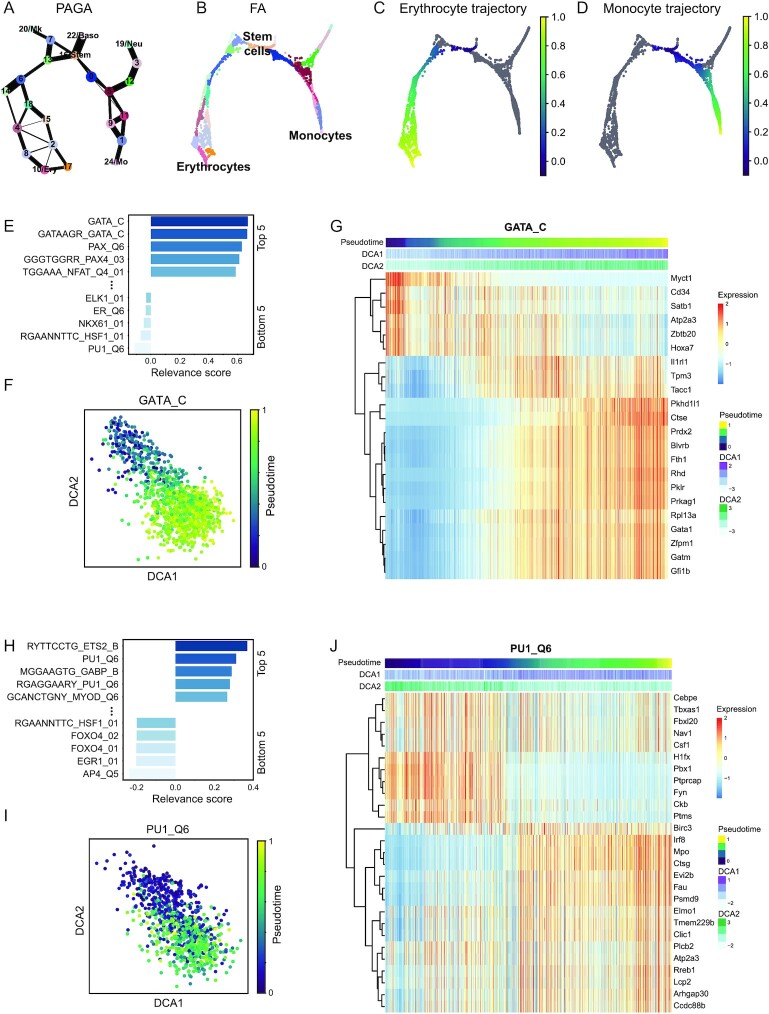
DrivAER unveils key transcription factors in blood development. PAGA (A) and cell-level graph (B) visualization of the Paul et al. [30] dataset. Cells are colored by Louvain clustering as provided by Scanpy. Two independent trajectories were calculated for erythrocyte (C) and monocyte (D) development. Cells are colored by pseudotime. (E) Bar plot displays relevance scores for the 5 most and least relevant transcription factors in the erythrocyte development trajectory. (F) DCA embedding plot was derived from the “GATA_C” gene set and is colored by pseudotime. (G) Heat map showing gene expression of cells and “GATA_C” target genes for the erythrocyte trajectory in columns and rows, respectively. (H) Bar plot displays relevance scores for the 5 most and least relevant transcription factors in the monocyte development trajectory. (I) DCA embedding plot was derived from the “PU1_Q6” gene set and is colored by pseudotime. (J) Heat map shows scaled gene expression of cells and “PU1_Q6” target genes for the monocyte trajectory in columns and rows, respectively. For both heat maps, columns are ordered by pseudotime. Bars on top of heat map indicate pseudotime, DCA coordinates 1 and 2. Red and blue colors reflect high and low expression values.

To demonstrate the utility of DrivAER, we use a collection of TF-target annotations to infer TF activity and reanalyzed a hematopoietic differentiation dataset by Paul et al. [30]. The authors identified and described the main blood development trajectories including differentiation from stem cells towards erythrocytes and monocytes (Fig. 2A and B). Next, we calculated 2 independent pseudotemporal trajectories for erythrocyte and monocyte differentiation (Fig. 2C and D). We then applied DrivAER to identify TFs that are relevant for erythrocyte and monocyte differentiation using the entire collection of motif gene sets contained in MolSigDB [31]. Among all 495 gene sets included in the analysis, DrivAER identified the GATA TF family as the most relevant in the erythrocyte trajectory (Fig. 2E, Supplementary Table S1). The DCA embedding derived from the “GATA_C” gene set showed strong clustering by pseudotime, demonstrating that GATA target gene expression is highly coordinated along this trajectory (Fig. 2F). Indeed, expression levels of GATA targets showed strong association with both pseudotime and DCA coordinates (Fig. 2G).

Of note, targets showed both up- and down-regulation. A fraction of targets increased in expression along the trajectory while a smaller fraction decreased. When integrating annotation from the TRRUST database [32] with the “GATAAGR_GATA_C” gene set, *Fli1* expression was predicted to be repressed by TF GATA1 and, correspondingly, we observed a negative correlation along the trajectory between these 2 genes (Supplementary Fig. S2).

Among the most relevant TFs in the monocyte trajectory was PU1 (Fig. 2H, Supplementary Table S1), which also showed strong association between the DCA embedding (Fig. 2J) and target gene expression (Fig. 2I) with pseudotime. Both GATA and PU1 are well-known lineage-determining regulators in blood development, with GATA and PU1 driving erythrocyte and monocyte differentiation, respectively [33]. However, such conclusions cannot be drawn on the basis of the expression of *Gata1* and *Pu1* itself. Although *Gata1* and *Pu1* showed increased expression along their developmental trajectories, many other TFs exhibited a similar pattern, making it difficult to pinpoint the driving regulator (Supplementary Fig. S3). Taken together, our findings demonstrate that DrivAER robustly explains the molecular mechanisms underlying complex biological processes.

### Benchmarking DrivAER

**Figure 3:**
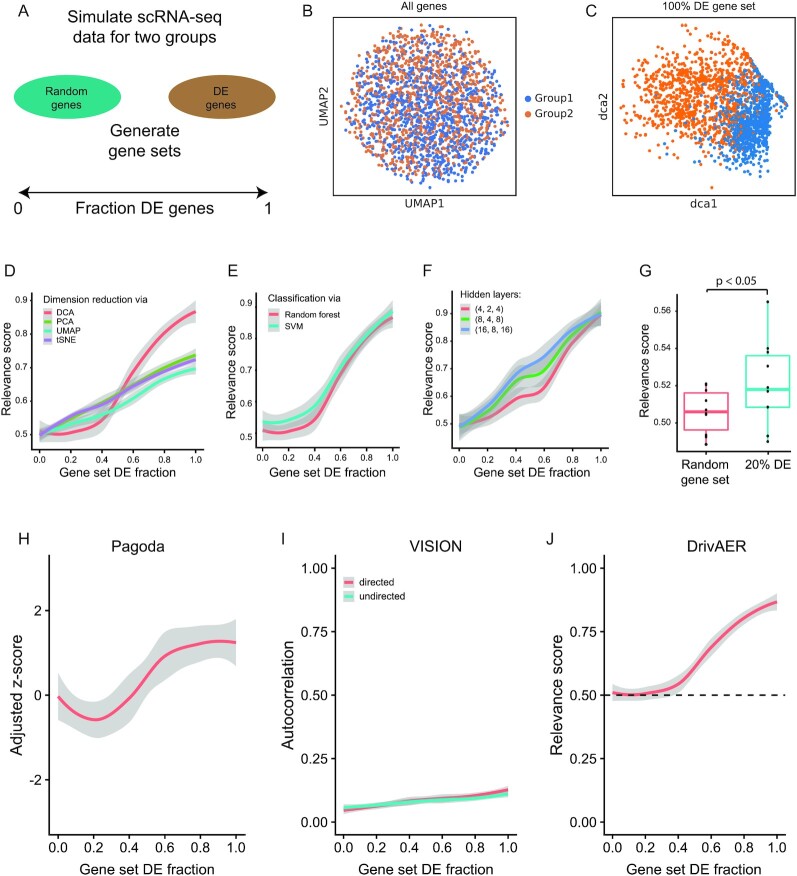
DrivAER identifies drivers underlying subtle transcriptional changes. (A) Two groups of single cells were simulated and gene sets were created by sampling a mixture of truly differentially expressed (DE) genes and random genes. (B) The global embedding using all genes is visualized using UMAP. (C) The DCA embedding for a gene set consisting of all truly DE genes is depicted. For both (B) and (C), cells are colored by group. (D) Relevance scores (y-axis) for gene sets ranging in the fraction of truly DE genes (x-axis) are displayed across implementations of DrivAER differing in the underlying dimension reduction methods. (E) Relevance scores (y-axis) for gene sets ranging in the fraction of truly DE genes (x-axis) are displayed using random forest (red) and support vector machine (SVM; blue) classification models. (F) Relevance scores (y-axis) for gene sets ranging in the fraction of truly DE genes (x-axis) are displayed across various configurations of the hidden layer. (G) Box plot shows significantly different relevance scores between 10 bootstrap runs of completely random gene sets (red) and gene sets consisting of 20% truly DE genes (blue) (1-sided *t*-test, *P* = 0.0467). The boxes represent the interquartile range, the horizontal line in the box is the median, and the whiskers represent 1.5 times the interquartile range. (H) PAGODA's adjusted z-scores (y-axis) are displayed for gene sets ranging in the fraction of truly DE genes (x-axis). (I) VISION's autocorrelation statistic is displayed for gene sets ranging in the fraction of truly DE genes (x-axis). (J) DrivAER (default parameters) relevance scores (y-axis) are displayed for gene sets ranging in the fraction of truly DE genes (x-axis). The horizontal dashed line indicates 0.5, the accuracy of random guesses for a binary outcome. For (D), (E), (F), (H), (I), and (J) lines represent the smoothed values and gray shading represents the 95% confidence interval derived from the smoothing fit.

To further assess and thoroughly benchmark our method in a controlled setting, we performed extensive simulation analysis. We used the Splatter [34] framework to simulate scRNA-seq data consisting of 2 groups of cells with subtle transcriptional differences where only 10% of genes were DE between the 2 groups. Subsequently, we generated different gene sets that varied in the number of truly DE genes (Fig. 3A). The gene sets ranged from sets without any truly DE genes (DE fraction = 0) to sets consisting of all truly DE genes (DE fraction = 1). Visualization of all genes in reduced dimensions using UMAP showed no clear separation between the 2 cell groups (Fig. 3B). However, dimension reduction restricted to truly DE genes using DCA showed separation between the cells (Fig. 3C), indicating that while the signal may be weak across all genes, targeted dimension reduction of specific genes successfully recovered the underlying cellular manifold.

To evaluate methodological aspects underlying DrivAER, we performed the following analyses. With respect to the dimension reduction task, we compared DCA with principal component analysis (PCA), Uniform Manifold Approximation and Projection (UMAP), and t-distributed stochastic neighbor embedding (tSNE). Across the gene sets that vary in the fraction of truly DE genes, DCA overall achieved the highest relevance scores (Fig. 3D). At low fractions of DE genes, the alternative dimension reduction methods slightly outperformed DCA (Supplementary Fig. S5). Therefore, we implemented PCA, UMAP, and tSNE-based dimension reduction into the DrivAER framework. Users have the option to select any of these 4 dimension reduction methods for their DrivAER analysis.

Next, we compared random forest and support vector machines (SVM) for the classification task. We did not observe any significant differences in performance between these 2 methods, indicating that random forest models represent an appropriate choice for this task (Fig. 3E). Moreover, we evaluated the impact of various hidden layer configurations during the DCA dimension reduction underlying DrivAER. We applied DrivAER using varying bottleneck layer sizes to the collection of simulated gene sets. The performance did not differ substantially across the 3 configurations, indicating that DrivAER is robust to various hidden layer configurations (Fig. 3F). Even when the gene set contained only 20% of truly DE genes, the relevance score was significantly higher than that of random gene sets over 10 bootstraps (1-sided *t*-test, *P* < 0.05, Fig. 3G), demonstrating DrivAER's ability to capture subtle transcriptional signals.

Additionally, we evaluated the different bottleneck configurations in a more complex simulation scenario consisting of 4 unbalanced groups of cells. All 3 configurations successfully recovered the varying degree of signal in the gene sets. The 4D and 8D bottleneck layers outperformed the 2D bottleneck layer slightly (Supplementary Fig. S6A). Visualization of the cellular manifold derived from the 2D, 4D, and 8D bottleneck layers showed improved separation of the 4 cell groups, suggesting that higher dimensional bottleneck layers may be needed to resolve more complex data manifolds (Fig. S6C and D).

Next, we compared DrivAER to VISION [22] and PAGODA [15], 2 existing tools for the functional interpretation of scRNA-seq data. Unlike DrivAER, VISION does not iteratively subject gene sets to dimension reduction; instead it operates directly on the global cellular manifold. VISION uses a local autocorrelation statistic to infer the relevance of various gene sets. PAGODA, on the other hand, calculates the adjusted z-score for each gene set and assesses the variance explained for significance. Both the autocorrelation and adjusted z-scores are analogous to the DrivAER relevance score. High values indicate relevant gene sets.

We applied VISION in directed and undirected mode, as well as PAGODA, to the simulated gene sets. As expected, for all 3 methods, the respective scores increased with the fraction of truly DE genes (Fig. 3H–J). However, it is important to note the following differences. For PAGODA, the gene sets with 20% truly DE genes achieved a lower adjusted z-score compared with the completely random gene sets. Moreover, the fact that none of the absolute adjusted z-scores passed 1.96 implied that none of the gene sets achieved statistical significance. For VISION, we observed a similar pattern. While the autocorrelation statistic increased with the fraction of truly DE genes, it never passed 0.1 and 100% truly DE gene sets never reached a high autocorrelation. Because VISION operates on the global manifold (i.e., Fig. 3B) instead of gene set–specific manifolds (i.e., Fig. 3C), it is less likely to capture subtle transcriptional differences. When using DrivAER, on the other hand, random gene sets achieved a relevance score ∼0.5. This corresponds to the likelihood of taking a random guess with 2 classes. Correspondingly, gene sets consisting of all truly DE genes approached relevance scores close to 1. Therefore, relevance scores for categorical phenotype can be readily interpreted.

For additional comparison, we applied VISION and PAGODA to the interferon stimulation and blood development datasets (Supplementary Fig. S4). All 3 methods clearly identified the correct TPs involved in interferon stimulation. In the erythrocyte trajectory, the GATA_C gene set achieved high scores using DrivAER and VISION but not PAGODA. For the monocyte trajectory, only DrivAER generated high relevance scores for PU1-related gene sets.

## Discussion and Conclusions

While autoencoders have been applied for unsupervised dimension reduction in bulk [35–37] and scRNA-seq data [38, 39], DrivAER makes use of autoencoders with a different goal. By iterative application, DrivAER scores gene sets on the basis of their relevance instead of trying to identify potential signatures that may not be captured in databases. Thus, while using autoencoders for unsupervised dimension reduction intrinsically, our method aims to rank gene sets in a supervised fashion.

Unlike VISION, DrivAER does not require a predefined distinction between the sign of regulation (repression or activation) of genes in a given gene set. The unsupervised nature of the DCA embedding captures any form of non-random, coordinated expression pattern. Therefore, DrivAER captures complex, non-linear expression patterns commonly observed in scRNA-seq data. An additional benefit of DrivAER is its ability to visualize the gene set–specific data manifold. These visualizations promote discovery of transcriptional regulation that may otherwise be hidden in the summary statistics generated by other methods including gene set enrichment analysis or VISION and PAGODA. Moreover, as demonstrated in the simulation analysis, DrivAER's relevance score is readily interpretable.

As illustrated in the blood development example, we divided the manifold into independent trajectories for interpretation. However, DrivAER provides the flexibility to be applied to the entire manifold or any subset of it. The user can make this choice and arbitrarily define regions of the manifold, which are expected to be regulated by a TP.

Additionally, as demonstrated in the blood development example, DrivAER enables users to make inferences about regulators that were not measured or where measurements are noisy. We envision that users will apply DrivAER to infer activity of regulators not generally detected in scRNA-seq data such as microRNAs and long noncoding RNAs.

In the present approach DCA needs to be retrained for each gene set because the input genes and thus the network architecture changes between gene sets. Therefore, the running time of DrivAER depends on the number of gene sets included in the analysis. In the interferon stimulation analysis, the running time per gene set averages between 20 and 30 seconds depending on the number of genes and convergence of the model. To improve speed, we plan to extend DrivAER by developing a “hot-start” approach in future work.

In summary, specialized methods facilitating the functional interpretation of scRNA-seq data are needed to fuel the rapid progress in the field. DrivAER is a novel machine learning approach that is effective for manifold interpretation in scRNA-seq data. Our results demonstrate that relevance scores represent a useful measure to extract driving transcriptional regulators from complex scRNA-seq datasets. DrivAER, including interactive use tutorial, is freely available from Github [40] and we anticipate broad use by the community.

## Methods

### Transcriptional program annotations

The Molecular Signatures Database (MolSigDB, v7.0) was used to define transcriptional programs [11]. The hallmark gene set contained 50 gene sets corresponding to specific well-defined biological processes [26]. The C3 TF targets collection contains 610 genes sets in total, where genes share the same *cis*-regulatory motifs from known TF binding sites in the TRANSFAC (v7.4) [41] database around their transcription start sites [31]. The gene sets with motifs not included in the TRANSFAC database were removed. A total of 495 gene sets were utilized in the blood development study. For mouse scRNA-seq datasets, the gene symbols were converted to mouse homologs before running DrivAER.

### DrivAER

DrivAER was written in Python and designed to integrate downstream of Scanpy [23]. Given a collection of annotated gene sets, DrivAER uses the DCA [9] to calculate a 2D data manifold for each gene set. Autoencoders are neural networks that learn an efficient compression of high-dimensional data [42]. One important characteristic that distinguishes DCA from other dimension reduction methods is a scRNA-seq–specific noise model. The bottleneck layer captures the compression and represents the data manifold. As default for DrivAER, we set the bottleneck dimension to 2 neurons. DCA takes a raw count matrix as input and outputs the data manifold coordinates using the parameter mode = “latent.” To account for differences in library size, size factors derived from the transcriptome-wide, instead of gene set–specific, expression matrix are fed into DCA.

The relevance scores are derived using random forest models as implemented in the Python module sklearn (v0.21.2). Once DCA has reduced the dimensions, the 2D data manifold coordinates are used as input features and the variable of interest as outcome in the random forest model. For categorical outcomes, “sklearn.ensemble.RandomForestClassifier” is used. For continuous outcomes, such as pseudotemporal trajectories, “sklearn.ensemble.RandomForestRegressor” is used. The number of trees was set to 500. The out-of-bag accuracy score of the TP-specific random forest model represents the relevance score.

For benchmarking purposes only, we applied SVM classification as implemented in the R package e1071 with default parameters. Additionally, we implemented 3 alternative dimension reduction methods into the DrivAER framework. PCA, tSNE, and UMAP were implemented using the Scanpy functions “pp.pca,” “tl.tsne,” and “tl.umap,” respectively. All functions use Scanpy's default parameters.

### Simulation analysis

scRNA-seq data were simulated using the splatter R package [34]. Specifically, the splatSimulate() function was used to simulate scRNA-seq data with 2 equally sized groups, consisting of 500 genes and 2,000 cells. The default gene expression and library size parameters were used. To simulate subtle transcriptional differences, the proportion of DE genes was set to 0.1 and the differential expression factor was set to 0.01. To include specific noise commonly encountered in scRNA-seq data, the dropout type was set to “experiment.” The “dropout.mid” parameter was set to 5 and “dropout.shape” was set to −1. The splatSimulate() function was also used to simulate scRNA-seq data with 4 unbalanced groups, containing 1,000 genes and 4,000 cells. The proportion of cell numbers in these 4 groups was set to 0.1, 0.2, 0.3, and 0.4, respectively. The “dropout.mid” parameter was set to 2 and “dropout.shape” was set to −1.

To simulate gene sets from a continuous spectrum of relevance the following approach was used. Gene sets were created by combining truly DE genes with genes showing no expression difference between the 2 groups. We generated gene sets containing 6 different fractions of truly DE gene sets (0, 0.2, 0.4, 0.6, 0.8, 1). Ten bootstrap samples were generated at each fraction. These 60 simulated gene sets were used for DrivAER evaluation.

For the evaluation of DrivAER using different configurations of hidden layers 5 bootstrap samples were generated at each fraction of truly DE gene sets. These 30 simulated gene sets were subjected to 3 different configurations of hidden layers, (4, 2, 4), (8, 4, 8), and (16, 8, 16), in an independent analysis.

### Interferon stimulation analysis

The scRNA-seq dataset of 29,065 peripheral blood mononuclear cells (PBMCs) from patients with lupus with and without interferon stimulation were obtained from the Gene Expression Omnibus database (GSE96583). The tSNE coordinates as well as cell type and state (stimulated or unstimulated) information displayed in Fig. 1E and F were taken from the supplemental materials of the original publication [25]. CD4 T cells were isolated on the basis of the cell type annotation file from the paper and DBSCAN clustering algorithm [43] was used to remove outlier cells (epsilon = 0.1, min_cells = 20). Before applying DrivAER, genes with low expression with <3 counts across all cells were filtered out.

### Blood development analysis

Expression data for the Paul et al. [30] data were obtained from Scanpy's (version 1.4.6) [23] built-in datasets using the “scanpy.datasets.paul15()” function. Expression data consist of 2,730 hematopoietic stem cells and 3,451 genes. The preprocessing of the data was performed following the Scanpy tutorial using the “scanpy.pp.recipe_zheng17()” function. Specifically, the 1,000 most highly variable genes were selected for downstream analysis. Louvain clustering (version 0.6.1) was conducted with resolution of 1, which resulted in 25 clusters. Clusters were annotated on the basis of the expression of canonical cell type marker genes. Two major developmental trajectories were identified, namely, the differentiation of hematopoietic stem cells to erythrocytes and monocytes. Pseudotemporal ordering was independently calculated for these 2 trajectories using the “scanpy.tl.dpt*”* function. DrivAER was applied to the raw counts and pseudotemporal ordering of each trajectory independently to infer relevant TPs.

Expression data for the Nestorowa dataset were obtained from the “Gene and protein expression in adult hematopoiesis” website [44]. On the basis of the provided annotation, cells were divided into the erythrocyte and monocyte trajectory. Pseudotemporal ordering was calculated as described above. DrivAER was applied as described above.

### PAGODA

PAGODA facilitates biological interpretation by testing gene sets for coordinated variability among cells. Briefly, PAGODA first estimates measurement properties, such as sequencing depth, drop-out rate, and amplification noise, for each cell. Next, PAGODA renormalizes the expression variance of each gene accounting for the measurement properties. Next, PAGODA tests whether a panel of gene sets shows statistically significant excess of coordinated variability using weighted PCA. A high dispersion or adjusted z-score indicates statistical significance and transcriptional heterogeneity of the gene set. The underlying idea is that overdispersed gene sets separate cells along a certain principal component. The separation of cells along this gene set–specific principal component implies relevance of the gene set.

The SCDE (version 1.99.1) R package including PAGODA was downloaded from github [45]. The gene set overdispersion analysis was conducted following the PAGODA tutorial with default parameters. The minimum number of reads for a gene was set to 2. The pagoda.varnorm() function was used to normalize the variance. The custom gene set environment file was created using the 60 simulated gene sets described above. The pagoda.pathway.wPCA() and pagoda.top.aspects() function were used to estimate the overdispersion of each gene set. The adjusted z-score was compared to DrivAER's relevance scores.

### VISION

VISION annotates sources of variation in scRNA-seq data by directly operating on the global cellular manifold. For each cell, VISION first identifies its closest k-nearest neighbor graph. By default, VISION uses PCA to create this low-dimensional space, but the user can provide more advanced latent space models. Next, VISION calculates a signature score for each annotated gene set and subsequently assesses whether the signature score is randomly distributed throughout the cellular manifold using a local autocorrelation statistic, the Geary C [46]. High values of VISION's autocorrelation indicate non-random pattern, and this score can be compared to DrivAER's relevance score. The input of VISION is the normalized count matrix and the signature files or objects containing various gene sets. The output is a VISION object containing the local autocorrelation scores for each gene set and corresponding embedding plot colored by scores.

VISION (version 2.0.0) was downloaded from github [47]. We applied VISION to the simulation analysis, interferon stimulation, and blood development experiments using default parameters. For the simulation analysis, VISION was run in both directed and undirected mode. The signature object was created using the 60 simulated gene sets described above. For the undirected mode, 1 was used for the value of every gene. For the directed mode, the values for the up- and down-regulated genes were set to 1 and −1, respectively. For the blood development experiments, VISION was run in trajectory mode following the pipeline of the VISION tutorial. After filtering and normalization, slingshot from the Dynverse package (version 0.1.1) [48, 49] was used to infer the trajectory. The VISION scores for each gene set were compared to DrivAER's relevance scores.

## Additional Files

**Supplementary Figure S1**. Heat map shows gene expression of PROTEIN_SECRETION gene set and cells in rows and columns, respectively. Columns are ordered first by stimulation status and second by DCA coordinates. Bars on top of heat map represent stimulation status and DCA coordinates 1 and 2. Red and blue colors correspond to high and low relative expression values.

**Supplementary Figure S2**. Plots show *Fli1* (top) and *Gata1* (bottom) expression along the erythrocyte trajectory. Grey points indicate cells. The green and blue lines represent smoothed expression estimates.

**Supplementary Figure S3**. Expression of *Gata1* (A) and *Gfi1b* (B) along the erythrocyte trajectory shows similar pattern. However, DCA embedding derived from “GFI1_01” gene set shows poor association with pseudotime. Expression of *Pu1* (A) and *Cebpa* (B) along the monocyte trajectory shows similar pattern. However, DCA embedding derived from “CEBPA_01” gene set shows poor association with pseudotime.

**Supplementary Figure S4**. (A) Scatter plots depict VISION autocorrelation statistic (x-axis) and DrivAER relevance scores (y-axis) for the Interferon stimulation (left), erythrocyte (middle), and monocyte (right) trajectories. (B) Scatter plots depict PAGODA adjusted z-scores (x-axis) and DrivAER relevance scores (y-axis) for the interferon stimulation (left), erythrocyte (middle), and monocyte (right) trajectories. For all panels, points represent gene sets and exemplary gene sets are highlighted.

**Supplementary Figure S5**. Box plot shows relevance scores between completely random gene sets (red) and gene sets containing 20% truly DE genes (blue) differing in the underlying dimension reduction methods. From left to right, dimension reduction was based on DCA, PCA, UMAP, and tSNE.

**Supplementary Figure S6**. Complex 4-group simulation analysis. (A) Relevance scores (y-axis) for gene sets ranging in the fraction of truly DE genes (x-axis) are displayed across various configurations of the hidden layer. The horizontal dashed line indicates 0.25, the accuracy of random guesses for an outcome with 4 categories. (B) The DCA embedding derived from a 2D bottleneck layer for a gene set consisting of all truly DE genes is depicted. Embedding derived from 4D (C) and 8D (D) bottleneck layers is visualized in 2 dimensions using UMAP. For (B–D), cells are colored by group.

**Supplementary Table S1**. Table contains the DrivAER relevance scores for the interferon stimulation and blood development experiments.

## Data Availability

An archival copy of the code and supporting data is available via the GigaScience repository, GigaDB [50].

## Availability of Supporting Source Code and Requirements

Project name: DrivAER

Project home page: https://github.com/lkmklsmn/DrivAER

Operating system(s): Platform independent

Programming language: Python

License: MIT license

bio.tools ID: drivaer

RRID: SCR_019076

## Abbreviations

DCA: deep count autoencoder; DE: differentially expressed; MolSigDB: Molecular Signatures Database; PCA: principal component analysis; PBMC: peripheral blood mononuclear cell; scRNA-seq: single-cell RNA sequencing; SVM: support vector machines; TF: transcription factor; TP: transcriptional program; tSNE: t-distributed stochastic neighbor embedding.

## Competing Interests

The authors declare that they have no competing interests.

## Funding

Z.Z. was partially supported by the National Institutes of Health [R01LM012806], Cancer Prevention and Research Institute of Texas [CPRIT RP180734], and The Chair Professorship for Precision Medicine Funds from the University of Texas Health Science Center at Houston. The funders had no role in the study design, data collection and analysis, decision to publish, or preparation of the manuscript.

## Authors' Contributions

L.M.S. conceived the idea and designed the project. L.M.S. and F.Y. analyzed the data. Z.Z. participated and supervised the project. All authors wrote the manuscript and read and approved the final manuscript.

## Supplementary Material

giaa122_GIGA-D-20-00038_Original_Submission

giaa122_GIGA-D-20-00038_Revision_1

giaa122_GIGA-D-20-00038_Revision_2

giaa122_GIGA-D-20-00038_Revision_3

giaa122_Response_to_Reviewer_Comments_Original_Submission

giaa122_Response_to_Reviewer_Comments_Revision_1

giaa122_Response_to_Reviewer_Comments_Revision_2

giaa122_Reviewer_1_Report_Original_Submission Christoph Ziegenhain -- 2/23/2020 Reviewed

giaa122_Reviewer_1_Report_Revision_1 Christoph Ziegenhain -- 6/4/2020 Reviewed

giaa122_Reviewer_2_Report_Original_Submission Casey S. Greene -- 2/25/2020 Reviewed

giaa122_Reviewer_3_Report_Original_Submission Vladimir V. Galatenko -- 2/28/2020 Reviewed

giaa122_Reviewer_3_Report_Revision_1 Vladimir V. Galatenko -- 6/8/2020 Reviewed

giaa122_Reviewer_3_Report_Revision_2 Vladimir V. Galatenko -- 9/21/2020 Reviewed

giaa122_Reviewer_4_Report_Original_Submission Andrew McDavid -- 3/5/2020 Reviewed

giaa122_Reviewer_4_Report_Revision_1 Andrew McDavid -- 6/5/2020 Reviewed

giaa122_Reviewer_4_Report_Revision_2 Andrew McDavid -- 9/12/2020 Reviewed

giaa122_Supplemental_File
