# Supplementary material for: DrivAER: Identification of driving transcriptional programs in single-cell RNA sequencing data
Source: Gigascience. 2020 Dec 10;9(12):giaa122. doi: 10.1093/gigascience/giaa122 (PMC7727875; doi:10.1093/gigascience/giaa122)
Supplement: giaa122_GIGA-D-20-00038_Original_Submission [file giaa122_giga-d-20-00038_original_submission.pdf]

GigaScience

DrivAER: Identification of driving transcriptional programs in single-cell RNA sequencing data

--Manuscript Draft--

**Manuscript Number:**	GIGA-D-20-00038
**Full Title:**	DrivAER: Identification of driving transcriptional programs in single-cell RNA sequencing data
**Article Type:**	Technical Note
**Funding Information:**	Cancer Prevention and Research Institute of Texas (RP180734) — Prof Lukas Simon, Prof Zhongming Zhao
**Abstract:**	Background: Single cell RNA sequencing unfolds complex transcriptomic data sets into detailed cellular maps. Despite recent success, there is a pressing need for specialized methods tailored towards the functional interpretation of these cellular maps.<br><br>Findings: Here, we present DrivAER, a machine learning approach that scores annotated gene sets based on their relevance to user-specified outcomes such as pseudotemporal ordering or disease status. DrivAER iteratively evaluates the information content of each gene set with respect to the outcome variable using autoencoders. We demonstrate that DrivAER extracts key pathways and transcription factors that regulate complex biological processes from scRNA-seq data.<br><br>Conclusions: By quantifying the relevance of annotated gene sets with respect to specified outcome variables, DrivAER greatly enhances our ability to understand the underlying molecular mechanisms.
**Corresponding Author:**	Lukas Simon, Ph.D.<br>University of Texas Health Science Center at Houston<br>Houston, UNITED STATES
**Corresponding Author Secondary Information:**	
**Corresponding Author's Institution:**	University of Texas Health Science Center at Houston
**Corresponding Author's Secondary Institution:**	
**First Author:**	Lukas Simon
**First Author Secondary Information:**	
**Order of Authors:**	Lukas Simon
	Fangfang Yan
	Zhongming Zhao
**Order of Authors Secondary Information:**	
**Additional Information:**	
**Question**	**Response**
Are you submitting this manuscript to a special series or article collection?	No
**Experimental design and statistics**	Yes

Powered by Editorial Manager® and ProduXion Manager® from Aries Systems Corporation

Full details of the experimental design and statistical methods used should be given in the Methods section, as detailed in our Minimum Standards Reporting Checklist. Information essential to interpreting the data presented should be made available in the figure legends.<br><br>Have you included all the information requested in your manuscript?	
**Resources**<br><br>A description of all resources used, including antibodies, cell lines, animals and software tools, with enough information to allow them to be uniquely identified, should be included in the Methods section. Authors are strongly encouraged to cite Research Resource Identifiers (RRIDs) for antibodies, model organisms and tools, where possible.<br><br>Have you included the information requested as detailed in our Minimum Standards Reporting Checklist?	Yes
**Availability of data and materials**<br><br>All datasets and code on which the conclusions of the paper rely must be either included in your submission or deposited in publicly available repositories (where available and ethically appropriate), referencing such data using a unique identifier in the references and in the "Availability of Data and Materials" section of your manuscript.<br><br>Have you have met the above requirement as detailed in our Minimum Standards Reporting Checklist?	Yes

Manuscript Click here to download Manuscript DrivAER- Gigascience.pdf

DrivAER: Identification of driving transcriptional programs in single-cell RNA sequencing data

Lukas M. Simon[1*+], Fangfang Yan[1], Zhongming Zhao[1,2,3,4*]

1. Center for Precision Health, School of Biomedical Informatics, The University of Texas Health Science Center at Houston, Houston, TX 77030, USA
2. Human Genetics Center, School of Public Health, The University of Texas Health Science Center at Houston, Houston, TX 77030, USA
3. MD Anderson Cancer Center UTHealth Graduate School of Biomedical Sciences, Houston, TX 77030, USA
4. Department of Biomedical Informatics, Vanderbilt University Medical Center, Nashville, TN 37203, USA

* To whom correspondence should be addressed: Lukas M. Simon (lukas.simon@uth.tmc.edu) and Zhongming Zhao (zhongming.zhao@uth.tmc.edu)

[+] Contact Lead

Abstract

Background: Single cell RNA sequencing unfolds complex transcriptomic data sets into detailed cellular maps. Despite recent success, there is a pressing need for specialized methods tailored towards the functional interpretation of these cellular maps.

Findings: Here, we present DrivAER, a machine learning approach that scores annotated gene sets based on their relevance to user-specified outcomes such as pseudotemporal ordering or disease status. DrivAER iteratively evaluates the information content of each gene set with respect to the outcome variable using autoencoders. We demonstrate that DrivAER extracts key pathways and transcription factors that regulate complex biological processes from scRNA-seq data.

Conclusions: By quantifying the relevance of annotated gene sets with respect to specified outcome variables, DrivAER greatly enhances our ability to understand the underlying molecular mechanisms.

Keywords

Autoencoder, machine learning, manifold interpretation, single-cell RNA sequencing, transcription factor

Findings

Background

Single cell RNA sequencing (scRNA-seq) experiments dissect biological processes or complex tissues at the cellular and molecular level [1,2]. Due to the high complexity and large number of observations, one critical step in scRNA-seq analysis is dimension reduction [3]. Dimension reduction projects the high dimensional expression matrix into a low dimensional space, also called data manifold or cellular map, which captures the underlying biological processes [4]. A number of methods have been used for manifold learning in scRNA-seq data [5–11].

Biological meaning can be extracted from the data manifold following in-depth analysis. After cells are stratified into separate groups or along a continuum, differential expression analysis is performed. The results can then be examined using tools for biological interpretation [12–14]. However, choosing the best parameters to identify differentially expressed genes across diverse scRNA-seq datasets is still an open challenge [15]. Therefore, there is a need for methods that facilitate biological interpretation without performing differential expression analysis.

Here, we present DrivAER, a method for the identification of Driving transcriptional programs based on AutoEncoder derived Relevance scores. Transcriptional programs (TPs) are sets of genes sharing biological properties [16], which have been annotated extensively, such as of genes sharing transcription factor binding motifs or genes involved in the same biological pathway[17,18]. DrivAER infers relevance scores for each TP with respect to specified outcomes of interest. These outcomes can represent extrinsic phenotypes, such as disease

status, or intrinsic phenotypes derived from the data itself such as pseudotemporal trajectories. Relevance scores allow researchers to rank TPs and help explain the underlying molecular mechanisms.

We evaluated DrivAER by application to two publicly available scRNA-seq datasets and comparison to a competing method called VISION [19]. Our results demonstrate that DrivAER correctly extracts well-known regulators from complex scRNA-seq datasets profiling interferon stimulation and blood development. Our user-friendly tool integrates smoothly downstream of the popular scRNA-seq analysis framework Scanpy [20].

Results

DrivAER correctly identifies interferon response

DrivAER is based on one assumption: the data manifold of relevant TPs shares information with the outcome of interest. Irrelevant TPs, on the other hand, will generate data manifolds where the cells fall randomly with respect to the outcome of interest. DrivAER builds upon our Deep Count Autoencoder (DCA) method [10] and iteratively applies DCA to the raw counts of each TP-specific gene set to generate a two-dimensional data manifold in an unsupervised manner (Fig. 1ab). Next, we associate the resulting manifold coordinates with the outcome of interest using random forest models (Fig. 1c). We interpret the random forest accuracy as relevance score, which quantifies the amount of information that is shared between the TP-specific data manifold and the outcome of interest (Fig. 1d).

To demonstrate the ability of DrivAER to perform correct manifold interpretation, we reanalyzed three publicly available scRNA-seq data sets. The first data set by Kang et al [21] described a transcriptional response to interferon stimulation (Fig. 1e). described a transcriptional response to interferon stimulation (Fig. 1e). As a proof of principle, we asked if

DrivAER could recapitulate this biology and extract the interferon signature as the driving transcriptional program defining the T cell data manifold (Fig. 1f). We applied DrivAER to the subset of T cells and evaluated 50 hallmark gene sets from MolSigDB [22] with respect to interferon stimulation (Table S1). Indeed, the “INTERFERON_GAMMA_RESPONE” gene set received the highest relevance score (Fig. 1g). Visualization of the T cell DCA embedding derived from the “INTERFERON_GAMMA_RESPONE” gene set showed clear separation by condition (Fig. 1h), implicating that this gene set is the main driving force separating the stimulated and unstimulated T cells. As a negative control, we show the DCA embedding for one of the lowest scoring gene sets “PROTEIN_SECRETION” (Fig. 1i). For this gene set, the cells cluster randomly with respect to the stimulation status. The heatmap in Figure 1j shows the expression levels of T cells for the “INTERFERON_GAMMA_RESPONSE” gene set. It is important to note that the cells (columns) are ordered by stimulation status and that the DCA coordinates are strongly associated with the stimulation status. Most “INTERFERON_GAMMA_RESPONSE” genes are upregulated in stimulated compared to unstimulated cells. Expression of genes in the “PROTEIN_SECRETION” gene set shows a random pattern (Fig. S1).

To further manifest the biological meaning of the DCA embedding, we visualized the expression of interferon marker IFIT2 in the “INTERFERON_GAMMA_RESPONSE” (Fig. 1k) and “PROTEIN_SECRETION” (Fig. 1l) embeddings. Expression levels of IFIT2 increase along the DCA coordinates in the “INTERFERON_GAMMA_RESPONSE” derived embedding. In contrast, IFIT2 expression is distributed randomly in the “PROTEIN_SECRETION” derived embedding.

DrivAER unveils key transcription factors in blood development

Next, we tested whether DrivAER is capable of extracting key transcription factors (TF) involved in differentiation trajectories. DrivAER is particularly well suited to infer the relevance of TFs for the following reasons. Firstly, TF mediated regulation is regarded as a combinatorial process which requires the coordination of multiple TFs and co-activators [23]. Secondly, due to the low RNA capture rate in scRNA-seq, generally lowly expressed TFs are not detected reliably [24]. Therefore, the expression levels of the target genes represent a better proxy of TF activity compared to the expression level of the TF itself [25].

To demonstrate the usage of DrivAER, we use a collection of TF-target annotations to infer TF activity and reanalyzed a hematopoietic differentiation dataset by Paul et al [26]. The authors identified and described the main blood development trajectories including differentiation from stem cells towards erythrocytes and monocytes (Fig. 2ab). Next, we calculated two independent pseudotemporal trajectories for erythrocyte and monocyte differentiation (Fig. 2cd). We then applied DrivAER to identify TFs that are relevant for erythrocyte and monocyte differentiation using the MolSigDB motif gene set [27]. DrivAER identified the GATA TF family as the most relevant in the erythrocyte trajectory (Fig. 2e, Table S1). The DCA embedding derived from the “GATA_C” gene set showed strong clustering by pseudotime, demonstrating that GATA target gene expression is highly coordinated along this trajectory (Fig. 2f). Indeed, expression levels of GATA targets show strong association with both pseudotime and DCA coordinates (Fig. 2g).

Of note, targets show both up and down-regulation. A fraction of targets increases in expression along the trajectory while a smaller fraction decreases. When integrating annotation from the TTRUST database [28] with the “GATAAGR_GATA_C” gene set, *Fli1* expression was

predicted to be repressed by TF GATA1 and correspondingly we observed a negative correlation along the trajectory between these two genes (Fig. S2).

Among the most relevant TFs in the monocyte trajectory was PU1 (Fig. 2h, Table S1), which also showed strong association between the DCA embedding (Fig. 2j) and target gene expression (Fig. 2i) with pseudotime. Both GATA and PU1 are well-known lineage determining regulators in blood development, with GATA and PU1 driving erythrocyte and monocyte differentiation, respectively [29]. However, such conclusions cannot be drawn based on the expression of *Gata1* and *Pu1* itself. Although *Gata1* and *Pu1* showed increased expression along their developmental trajectories, many other TFs exhibited a similar pattern, making it difficult to pinpoint the driving regulator (Fig. S3).

Furthermore, to validate our findings we repeated the analysis in a second, independent blood development scRNA-seq data set [30]. In this second data set, DrivAER correctly identified the gene sets “GATA_C” and “PU1_Q6” as the most relevant TFs for erythrocyte and monocyte differentiation, respectively. Taken together, our findings demonstrate that DrivAER robustly explains the molecular mechanisms underlying complex biological processes.

DrivAER comparison with VISION

We compared DrivAER to VISION [19] for all three analyses (Fig. S4). We observed strong correlation between the relevance scores derived from DivAER and the signature scores derived from VISION for the interferon stimulation experiment, confirming the validity of both methods (Pearson’s correlation, Rho 0.86, p <1e-10). While no significant correlation was observed in the blood development experiments, both methods correctly identified GATA and PU1 related gene sets among the highest scoring TFs for the erythrocyte and monocyte trajectories, respectively. However, VISION scored GATA and PU1 highly in both trajectories,

whereas high DrivAER relevance scores were specific to the corresponding trajectories, indicating increased specificity.

Discussion and conclusions

Unlike VISION, DrivAER does not require a pre-defined distinction between the sign of regulation (repression or activation) of genes in a given gene set. The unsupervised nature of the DCA embedding captures any form of non-random, coordinated expression pattern. Therefore, DrivAER captures complex, non-linear expression patterns commonly observed in scRNA-seq data. In principle, the DCA step can be replaced with any other dimension reduction method and future work will focus on potential extensions of DrivAER by integrating different dimension reduction approaches. As illustrated in the blood development example we divided the manifold into independent trajectories for interpretation. However, DrivAER provides the flexibility to be applied to the entire manifold or any subset of it. The user can make this choice and arbitrarily define regions of the manifold, which are expected to be regulated by a TP.

Additionally, as demonstrated in the blood development example, DrivAER enables users to make inferences about regulators that were not measured or where measurements are noisy. We envision that users will apply DrivAER to infer activity of regulatory species not generally detected in scRNAseq data such as microRNAs and long noncoding RNAs.

In summary, specialized methods facilitating the functional interpretation of scRNA-seq data are needed to fuel progress in the field. DrivAER is a novel machine learning approach that is effective for manifold interpretation in scRNA-seq data. Our results demonstrate that relevance scores represent a useful measure to extract driving transcriptional regulators from complex scRNA-seq data sets. DrivAER, including interactive usage tutorial, is freely available

from Github (https://github.com/lkmklsmn/DrivAER) and we anticipate broad usage by the community.

Methods

Transcriptional program annotations

The Molecular Signatures Database (MolSigDB , v7.0) was used to define transcriptional programs [18]. The Hallmark gene set contained 50 gene sets corresponding to specific well-defined biological processes [22]. The C3 transcription factor targets collection contains 610 genes sets in total, where genes share the same *cis*-regulatory motifs from known TF binding sites in the TRANSFAC (v7.4) [31] database around their transcription start sites [27]. The gene sets with motifs not included in the TRANSFAC database were removed. A total of 495 gene sets were utilized in the blood development study. For mouse scRNA-seq datasets, the gene symbols were converted to mouse homologs before running DrivAER.

DrivAER

DrivAER was written in Python and designed to integrate downstream of Scanpy[20]. Given a collection of gene sets, DrivAER uses the Deep Count Autoencoder (DCA) [10] to calculate a two-dimensional data manifold for each gene set. Autoencoders are neural networks that learn an efficient compression of high dimensional data [32]. One important characteristic that distinguishes DCA from other dimension reduction methods is a scRNA-seq specific noise model. The bottleneck layer captures the compression and represents the data manifold. As default for DrivAER, we set the bottleneck dimension to two neurons. DCA takes raw count matrix as input and outputs the data manifold coordinates using the parameter mode = “latent”.

To account for differences in library size, size factors derived from the transcriptome-wide, instead of gene set specific, expression matrix are being fed into DCA.

The relevance scores are derived using random forest models as implemented in the Python module sklearn (v0.21.2). Once DCA has reduced the dimensions, the two-dimensional data manifold coordinates are used as input features and the variable of interest as outcome in the random forest model. The number of trees was set to 500. The Out-of-Bag accuracy score of the TP-specific random forest model represents the relevance score.

Interferon stimulation analysis

The scRNA-seq data set of 29,065 PBMCs from lupus patients with and without interferon stimulation were obtained from the GEO database (GSE96583). The t-Distributed Stochastic Neighbor Embedding (tSNE) coordinates in the dataset were used to create the embedding plot, as well as cell type and cell state (stimulated or unstimulated). CD4 T cells were isolated and DBSCAN clustering algorithm [33] was utilized to remove outlier cells (epsilon = 0.1, min_cells = 20). Before applying DrivAER method, lowly expressed genes and cells with less than 3 counts were filtered out.

Blood development analysis

Expression data for the Paul et al data set was obtained from Scanpy [20] builtin datasets using the "*scanpy.datasets.paul15()"* function. Expression data consists of 2730 hematopoietic stem cells and 3451 genes. The 1000 most highly variable genes were selected for downstream analysis. Louvain clustering, cell type annotation, and diffusion pseudotime calculation was performed following the Scanpy tutorial. Two major developmental trajectories were identified, namely the differentiation of hematopoietic stem cells to erythrocytes and

monocytes. Pseudotemporal ordering was independently calculated for these two trajectories using the "*scanpy.tl.dpt"* function. DrivAER was applied to the raw counts and pseudotemporal ordering of each trajectory independently to infer relevant TPs.

Expression data for the Nestorowa data set was obtained from the "Gene and protein expression in adult hematopoiesis" website (http://blood.stemcells.cam.ac.uk/single_cell_atlas). Based on the provided annotation, cells were divided into the erythrocyte and monocyte trajectory. Pseudotemporal ordering was calculated as described above. DrivAER was applied as described above.

VISION

VISION (version 2.0.0) was downloaded from github (https://github.com/yoseflab/VISION). VISION incorporating a trajectory mode was run on the blood development data following the pipeline of the VISION tutorial. After filtering and normalization, slingshot from the Dynverse package [34] (https://github.com/dynverse/dyno) was used to infer the trajectory and the milestone network. The gene signatures selected by VISION were compared to our results.

Abbreviations

DCA: deep count autoencoder; MolSigDB: the Molecular Signatures Database; scRNA-seq: single cell RNA sequencing; TF: transcription factor; TP: transcriptional program; tSNE: t-distributed Stochastic Neighbor Embedding.

Declarations

Ethics approval and consent to participate

Not applicable

Consent for publication

Not applicable

Availability of data and materials

DrivAER is available via Github (https://github.com/lkmklsmn/DrivAER).

Competing interests

The authors declare that they have no competing interests.

Funding

The work was supported by the National Institutes of Health [R01LM012806, R03DE027393, R03DE028103], Cancer Prevention and Research Institute of Texas [CPRIT RP180734], and The Chair Professorship for Precision Medicine Funds from the University of Texas Health Science Center at Houston. The funders had no role in the study design, data collection and analysis, decision to publish, or preparation of the manuscript.

Authors' contributions

LS conceived the idea and designed the project. LS and FY analyzed the data. ZZ supervised the project. LS, FY, ZZ wrote the manuscript. All authors read and approved the final manuscript.

Acknowledgements

The authors would like to thank the members of the Bioinformatics and Systems Medicine Laboratory at the University of Texas Health Science Center at Houston for stimulating discussion.

References

1. Trapnell C. Defining cell types and states with single-cell genomics. Genome Res. 2015;25:1491–8.

2. Hwang B, Lee JH, Bang D. Single-cell RNA sequencing technologies and bioinformatics pipelines [Internet]. Experimental & Molecular Medicine. 2018. Available from: http://dx.doi.org/10.1038/s12276-018-0071-8

3. Luecken MD, Theis FJ. Current best practices in single-cell RNA-seq analysis: a tutorial. Mol Syst Biol. 2019;15:e8746.

4. Moon KR, Stanley JS, Burkhardt D, van Dijk D, Wolf G, Krishnaswamy S. Manifold learning-based methods for analyzing single-cell RNA-sequencing data [Internet]. Current Opinion in Systems Biology. 2018. p. 36–46. Available from: http://dx.doi.org/10.1016/j.coisb.2017.12.008

5. Coifman RR, Lafon S, Lee AB, Maggioni M, Nadler B, Warner F, et al. Geometric diffusions as a tool for harmonic analysis and structure definition of data: diffusion maps. Proc Natl Acad

Sci U S A. 2005;102:7426–31.

6. Haghverdi L, Buettner F, Theis FJ. Diffusion maps for high-dimensional single-cell analysis of differentiation data. Bioinformatics. 2015;31:2989–98.

7. Pearson K. LIII. On lines and planes of closest fit to systems of points in space [Internet]. The London, Edinburgh, and Dublin Philosophical Magazine and Journal of Science. 1901. p. 559–72. Available from: http://dx.doi.org/10.1080/14786440109462720

8. van der Maaten L, Hinton G. Visualizing data using t-SNE. J Mach Learn Res. 2008;2579–605.

9. Lopez R, Regier J, Cole MB, Jordan MI, Yosef N. Deep generative modeling for single-cell transcriptomics. Nat Methods. 2018;15:1053–8.

10. Eraslan G, Simon LM, Mircea M, Mueller NS, Theis FJ. Single-cell RNA-seq denoising using a deep count autoencoder. Nat Commun. 2019;10:390.

11. McInnes L, Healy J, Saul N, Großberger L. UMAP: Uniform Manifold Approximation and Projection [Internet]. Journal of Open Source Software. 2018. p. 861. Available from: http://dx.doi.org/10.21105/joss.00861

12. Fan J, Salathia N, Liu R, Kaeser GE, Yung YC, Herman JL, et al. Characterizing transcriptional heterogeneity through pathway and gene set overdispersion analysis. Nat Methods. 2016;13:241–4.

13. Buettner F, Pratanwanich N, McCarthy DJ, Marioni JC, Stegle O. f-scLVM: scalable and versatile factor analysis for single-cell RNA-seq. Genome Biol. 2017;18:212.

14. Martignetti L, Calzone L, Bonnet E, Barillot E, Zinovyev A. ROMA: Representation and Quantification of Module Activity from Target Expression Data. Front Genet. 2016;7:18.

15. Wang T, Li B, Nelson CE, Nabavi S. Comparative analysis of differential gene expression analysis tools for single-cell RNA sequencing data. BMC Bioinformatics. 2019;20:40.

16. Heimberg G, Bhatnagar R, El-Samad H, Thomson M. Low Dimensionality in Gene Expression Data Enables the Accurate Extraction of Transcriptional Programs from Shallow Sequencing. Cell Syst. 2016;2:239–50.

17. Kanehisa M. KEGG: Kyoto Encyclopedia of Genes and Genomes [Internet]. Nucleic Acids Research. 2000. p. 27–30. Available from: http://dx.doi.org/10.1093/nar/28.1.27

18. Subramanian A, Tamayo P, Mootha VK, Mukherjee S, Ebert BL, Gillette MA, et al. Gene set enrichment analysis: a knowledge-based approach for interpreting genome-wide expression profiles. Proc Natl Acad Sci U S A. 2005;102:15545–50.

19. DeTomaso D, Jones MG, Subramaniam M, Ashuach T, Ye CJ, Yosef N. Functional interpretation of single cell similarity maps. Nat Commun. 2019;10:4376.

20. Wolf FA, Angerer P, Theis FJ. SCANPY: large-scale single-cell gene expression data

analysis. Genome Biol. 2018;19:15.

21. Kang HM, Subramaniam M, Targ S, Nguyen M, Maliskova L, McCarthy E, et al. Multiplexed droplet single-cell RNA-sequencing using natural genetic variation. Nat Biotechnol. 2018;36:89–94.

22. Liberzon A, Birger C, Thorvaldsdóttir H, Ghandi M, Mesirov JP, Tamayo P. The Molecular Signatures Database Hallmark Gene Set Collection [Internet]. Cell Systems. 2015. p. 417–25. Available from: http://dx.doi.org/10.1016/j.cels.2015.12.004

23. Zhu Z, Shendure J, Church GM. Discovering functional transcription-factor combinations in the human cell cycle. Genome Res. 2005;15:848–55.

24. Hicks SC, Townes FW, Teng M, Irizarry RA. Missing data and technical variability in single-cell RNA-sequencing experiments. Biostatistics. 2018;19:562–78.

25. Schacht T, Oswald M, Eils R, Eichmüller SB, König R. Estimating the activity of transcription factors by the effect on their target genes. Bioinformatics. 2014;30:i401–7.

26. Paul F, Arkin Y 'ara, Giladi A, Jaitin DA, Kenigsberg E, Keren-Shaul H, et al. Transcriptional Heterogeneity and Lineage Commitment in Myeloid Progenitors. Cell. 2016;164:325.

27. Xie X, Lu J, Kulbokas EJ, Golub TR, Mootha V, Lindblad-Toh K, et al. Systematic discovery of regulatory motifs in human promoters and 3′ UTRs by comparison of several mammals [Internet]. Nature. 2005. p. 338–45. Available from: http://dx.doi.org/10.1038/nature03441

28. Han H, Cho J-W, Lee S, Yun A, Kim H, Bae D, et al. TRRUST v2: an expanded reference database of human and mouse transcriptional regulatory interactions. Nucleic Acids Res. 2018;46:D380–6.

29. Monteiro R, Pouget C, Patient R. The gata1/pu.1 lineage fate paradigm varies between blood populations and is modulated by tif1γ. EMBO J. 2011;30:1093–103.

30. Nestorowa S, Hamey FK, Sala BP, Diamanti E, Shepherd M, Laurenti E, et al. A single-cell resolution map of mouse hematopoietic stem and progenitor cell differentiation [Internet]. Blood. 2016. p. e20–31. Available from: http://dx.doi.org/10.1182/blood-2016-05-716480

31. Matys V, Kel-Margoulis OV, Fricke E, Liebich I, Land S, Barre-Dirrie A, et al. TRANSFAC and its module TRANSCompel: transcriptional gene regulation in eukaryotes. Nucleic Acids Res. 2006;34:D108–10.

32. Hinton GE, Salakhutdinov RR. Reducing the dimensionality of data with neural networks. Science. 2006;313:504–7.

33. Louhichi S, Gzara M, Ben Abdallah H. A density based algorithm for discovering clusters with varied density [Internet]. 2014 World Congress on Computer Applications and Information Systems (WCCAIS). 2014. Available from: http://dx.doi.org/10.1109/wccais.2014.6916622

34. Saelens W, Cannoodt R, Todorov H, Saeys Y. A comparison of single-cell trajectory inference methods. Nat Biotechnol. 2019;37:547–54.

Figure Legends

Figure 1. DrivAER correctly identifies interferon response. (a) DrivAER iteratively subjects annotated gene sets to unsupervised dimension reduction via Deep Count Autoencoder (DCA). (b) For each gene set, the generated two-dimensional data manifold coordinates are used as input features in a random forest model to predict the outcome of interest (i.e. pseudotemporal ordering) (c). (d) The random forest prediction accuracy represents the relevance score. (e) t-Distributed Stochastic Neighbor Embedding (tSNE) visualization displays all PBMC cells colored by cell type. (f) Cellular map (tSNE) of T cell subset clusters by stimulation status. (g) Barplot indicates relevance scores of the top and bottom five transcription programs. DCA embedding calculated based on “INTERFERON_GAMMA_RESPONSE” (h) and “PROTEIN_SECRETION” (i) (negative control) gene sets. Cells are colored by stimulation status. (j) Heatmap shows gene expression of “INTERFERON_GAMMA_RESPONSE” target genes and cells in rows and columns, respectively. Columns are ordered first by stimulation status and second by DCA coordinates. Bars on top of heatmap represent stimulation status and DCA coordinates one and two. Red and blue colors correspond to high and low relative expression values. Relative expression of interferon gene IFIT2 is overlaid on top of the DCA embeddings derived from “INTERFERON_GAMMA_RESPONSE” (k) and “PROTEIN_SECRETION” (l) gene sets. Dark colors indicate higher expression.

Figure 2. DrivAER unveils key transcription factors in blood development. PAGA (a) and cell-level graph (b) visualization of the Paul et al data set. Cells are colored by Louvain cluster

as provided by Scanpy. Two independent trajectories were calculated for erythrocyte (c) and monocyte (d) development. Cells are colored by pseudotime. (e) Barplot displays relevance scores for the 5 most and least relevant transcription factors in the erythrocyte development trajectory. (f) DCA embedding plot was derived from the “GATA_C” gene set and is colored by pseudotime. (g) Heatmap showing gene expression of cells and “GATA_C” target genes for the erythrocyte trajectory in columns and rows, respectively. (h) Barplot displays relevance scores for the 5 most and least relevant transcription factors in the monocyte development trajectory. (i) DCA embedding plot was derived from the “PU1_Q6” gene set and is colored by pseudotime. (j) Heatmap shows scaled gene expression of cells and “PU1_Q6” target genes for the monocyte trajectory in columns and rows, respectively. For both heatmaps, columns are ordered by pseudotime. Bars on top of heatmap indicate pseudotime, DCA coordinates one and two. Red and blue colors reflect high and low expression values.

Supplemental table Click here to download Table Table_S1.xlsx

Gene set	Relevance score
INTERFERON_GAMMA_RESPONSE	0.978803539
INTERFERON_ALPHA_RESPONSE	0.962336866
KRAS_SIGNALING_DN	0.897871595
INFLAMMATORY_RESPONSE	0.800648156
ESTROGEN_RESPONSE_LATE	0.762371902
APOPTOSIS	0.734956644
ANDROGEN_RESPONSE	0.720679688
IL2_STAT5_SIGNALING	0.637995971
IL6_JAK_STAT3_SIGNALING	0.62809845
ALLOGRAFT_REJECTION	0.623281072
HYPOXIA	0.621967242
GLYCOLYSIS	0.6160988
CHOLESTEROL_HOMEOSTASIS	0.60077078
COMPLEMENT	0.589209074
XENOBIOTIC_METABOLISM	0.568625734
EPITHELIAL_MESENCHYMAL_TRANSITION	0.554173601
G2M_CHECKPOINT	0.553648069
TNFA_SIGNALING_VIA_NFKB	0.552071472
ESTROGEN_RESPONSE_EARLY	0.546027853
WNT_BETA_CATENIN_SIGNALING	0.544100902
FATTY_ACID_METABOLISM	0.538582815
BILE_ACID_METABOLISM	0.531050188
COAGULATION	0.526758343
TGF_BETA_SIGNALING	0.525006569
REACTIVE_OXYGEN_SPECIES_PATHWAY	0.522466497
UV_RESPONSE_DN	0.519838837
APICAL_JUNCTION	0.517035999
MYC_TARGETS_V2	0.51694841
UNFOLDED_PROTEIN_RESPONSE	0.515371814
ADIPOGENESIS	0.513357274
PEROXISOME	0.513357274
P53_PATHWAY	0.513182097
MTORC1_SIGNALING	0.510817202
DNA_REPAIR	0.509766138
MYC_TARGETS_V1	0.509766138
HEME_METABOLISM	0.50897784
KRAS_SIGNALING_UP	0.507226066
MYOGENESIS	0.505999825
SPERMATOGENESIS	0.505824647
E2F_TARGETS	0.505737059
OXIDATIVE_PHOSPHORYLATION	0.505474293
PI3K_AKT_MTOR_SIGNALING	0.505123938
UV_RESPONSE_UP	0.504598406
MITOTIC_SPINDLE	0.503459753
PROTEIN_SECRETION	0.498905141

Gene set	Relevance score
GATA_C	0.676061042
GATAAGR_GATA_C	0.671994949
PAX_Q6	0.629690979
GGGTGGRR_PAX4_03	0.616527372
TGGAAA_NFAT_Q4_01	0.597276829
TGAYRTCA_ATF3_Q6	0.5686248
GATTGGY_NFY_Q6_01	0.531426105
RCGCANGCGY_NRF1_Q6	0.528429682
ETS_Q4	0.516024485
PEA3_Q6	0.510763526
RYTTCCTG_ETS2_B	0.508509968
GTGACGY_E4F1_Q6	0.49992403
CAGCTG_AP4_Q5	0.493708331
GATA3_01	0.485018113
GCANCTGNY_MYOD_Q6	0.480897011
TGCCAAR_NF1_Q6	0.448316161
EGR1_01	0.434745652
GATA1_05	0.429795408
COREBINDINGFACTOR_Q6	0.42973298
ELF1_Q6	0.428789552
CACGTG_MYC_Q2	0.426055498
AP1_01	0.422997571
CEBP_01	0.386955685
MYCMAX_01	0.378398179
RACCACAR_AML_Q6	0.366473396
CTAWWWATA_RSRFC4_Q2	0.360797139
TGANTCA_AP1_C	0.355824033
IRF_Q6	0.352811591
RTAAACA_FREAC2_01	0.346485949
NMYC_01	0.320041259
TAATTA_CHX10_01	0.319906459
TGTTTGY_HNF3_Q6	0.318758421
CTTTGA_LEF1_Q2	0.316418727
ETS1_B	0.316362778
PXR_Q2	0.311710892
STAT6_02	0.311670412
ZIC1_01	0.308302577
USF_01	0.301699954
NFE2_01	0.298671551
TCANNTGAY_SREBP1_01	0.297022418
STAT4_01	0.296446986
BACH1_01	0.278809715
TGACCTY_ERR1_Q2	0.255904782
AML_Q6	0.255183517
FOXO1_02	0.249533266
ISRE_01	0.248459257

RSRFC4_01	0.241928544
AP1_Q6_01	0.209253089
TFIIA_Q6	0.208237414
MGGAAGTG_GABP_B	0.203925163
STAT5A_03	0.194259193
CATTGTYY_SOX9_B1	0.194086314
NFKB_Q6_01	0.186115225
MYCMAX_03	0.179490579
SP1_Q4_01	0.174298688
NERF_Q2	0.173323913
AP1_Q6	0.168829015
FREAC3_01	0.163954467
HNF4_01	0.163851528
OCT1_04	0.154132745
CAGGTA_AREB6_01	0.153707217
STTTCRNTTT_IRF_Q6	0.149240359
YTATTTTNR_MEF2_02	0.142215952
MYOD_01	0.133072202
TGACAGNY_MEIS1_01	0.13253603
AP4_Q5	0.126144979
SRF_Q5_01	0.12191897
TATAAA_TATA_01	0.121652392
FOXO4_02	0.112154493
RGAGGAARY_PU1_Q6	0.111981958
LYF1_01	0.10850511
LEF1_Q6	0.095790211
SCGGAAGY_ELK1_02	0.095629061
NFKAPPAB_01	0.09078697
CREL_01	0.088061172
AP1_Q4_01	0.071420661
CDC5_01	0.05677993
SOX9_B1	0.04751447
FOXO4_01	0.043265412
IRF1_Q6	0.038900095
ETS2_B	0.034986842
GCCATNTTG_YY1_Q6	0.03336043
GATA6_01	0.022830519
GGATTA_PITX2_Q2	0.022047245
NFKB_C	0.018558804
E47_01	-0.013219445
AML1_01	-0.02350686
AML1_Q6	-0.023637853
NFKB_Q6	-0.024010175
ELK1_01	-0.026496317
ER_Q6	-0.033773939
NKX61_01	-0.042069939
RGAANNTTC_HSF1_01	-0.068157131

PU1_Q6	-0.116171642

Gene set	Relevance score
RYTTCCTG_ETS2_B	0.370270325
PU1_Q6	0.312744375
MGGAAGTG_GABP_B	0.289342855
RGAGGAARY_PU1_Q6	0.280535165
GCANCTGNY_MYOD_Q6	0.26677336
RCGCANGCGY_NRF1_Q6	0.236091809
AML_Q6	0.227749938
CAGCTG_AP4_Q5	0.224238916
STAT4_01	0.221227266
TGGAAA_NFAT_Q4_01	0.213876266
RACCACAR_AML_Q6	0.192545186
AP1_Q6_01	0.173389806
TAATTA_CHX10_01	0.162711397
COREBINDINGFACTOR_Q6	0.148602239
GATTGGY_NFY_Q6_01	0.132232415
AML1_Q6	0.112233987
AML1_01	0.108789572
STAT6_02	0.097143807
TATAAA_TATA_01	0.092828696
RTAAACA_FREAC2_01	0.091584665
NERF_Q2	0.088856449
IRF1_Q6	0.054554057
E47_01	0.051143923
CEBP_01	0.050008329
CACGTG_MYC_Q2	0.044539518
ISRE_01	0.042293206
BACH1_01	0.040158557
NFKB_C	0.036678475
PEA3_Q6	0.030412765
TCANNTGAY_SREBP1_01	0.029216858
GGGTGGRR_PAX4_03	0.012754209
AP1_Q6	0.01018262
TGACCTY_ERR1_Q2	-0.00036459
CTTTGA_LEF1_Q2	-0.004072902
MYOD_01	-0.00605501
ER_Q6	-0.013616799
SCGGAAGY_ELK1_02	-0.016435983
CTAWWWATA_RSRFC4_Q2	-0.016927525
FREAC3_01	-0.019203989
ELF1_Q6	-0.029666136
TGCCAAR_NF1_Q6	-0.045966665
CATTGTYY_SOX9_B1	-0.0506712
TGAYRTCA_ATF3_Q6	-0.050749514
ETS_Q4	-0.054884973
GATA3_01	-0.054914687
GATAAGR_GATA_C	-0.058584169

TGTTTGY_HNF3_Q6	-0.058649911
NKX61_01	-0.059222149
AP1_01	-0.059398517
LEF1_Q6	-0.063168211
SRF_Q5_01	-0.063798844
IRF_Q6	-0.064334499
PXR_Q2	-0.069974965
ETS2_B	-0.070243027
GTGACGY_E4F1_Q6	-0.0736639
GATA1_05	-0.075997473
SP1_Q4_01	-0.078006799
YTATTTTNR_MEF2_02	-0.082815783
NFKB_Q6_01	-0.086414753
MYCMAX_03	-0.087704652
STTTCRNTTT_IRF_Q6	-0.091302723
GATA_C	-0.091530083
ELK1_01	-0.092054224
HNF4_01	-0.096824605
ZIC1_01	-0.100549593
TGACAGNY_MEIS1_01	-0.103357495
NFKAPPAB_01	-0.111300438
OCT1_04	-0.114053039
MYCMAX_01	-0.114062163
TGANTCA_AP1_C	-0.114672035
NFE2_01	-0.127434393
CREL_01	-0.129942472
CAGGTA_AREB6_01	-0.134277338
CDC5_01	-0.135756421
GCCATNTTG_YY1_Q6	-0.142845368
AP1_Q4_01	-0.149670406
NFKB_Q6	-0.152961146
LYF1_01	-0.153175885
STAT5A_03	-0.154672467
GATA1_04	-0.15671949
FOXO1_02	-0.157988658
ETS1_B	-0.160057028
SOX9_B1	-0.164957888
GATA6_01	-0.167653588
RSRFC4_01	-0.172971638
TFIIA_Q6	-0.173194606
GGATTA_PITX2_Q2	-0.173294053
RGAANNTTC_HSF1_01	-0.199029506
FOXO4_02	-0.199854909
FOXO4_01	-0.20208031
EGR1_01	-0.203545637
AP4_Q5	-0.239254801

Figure 1 Click here to download Figure Figure1.pdf

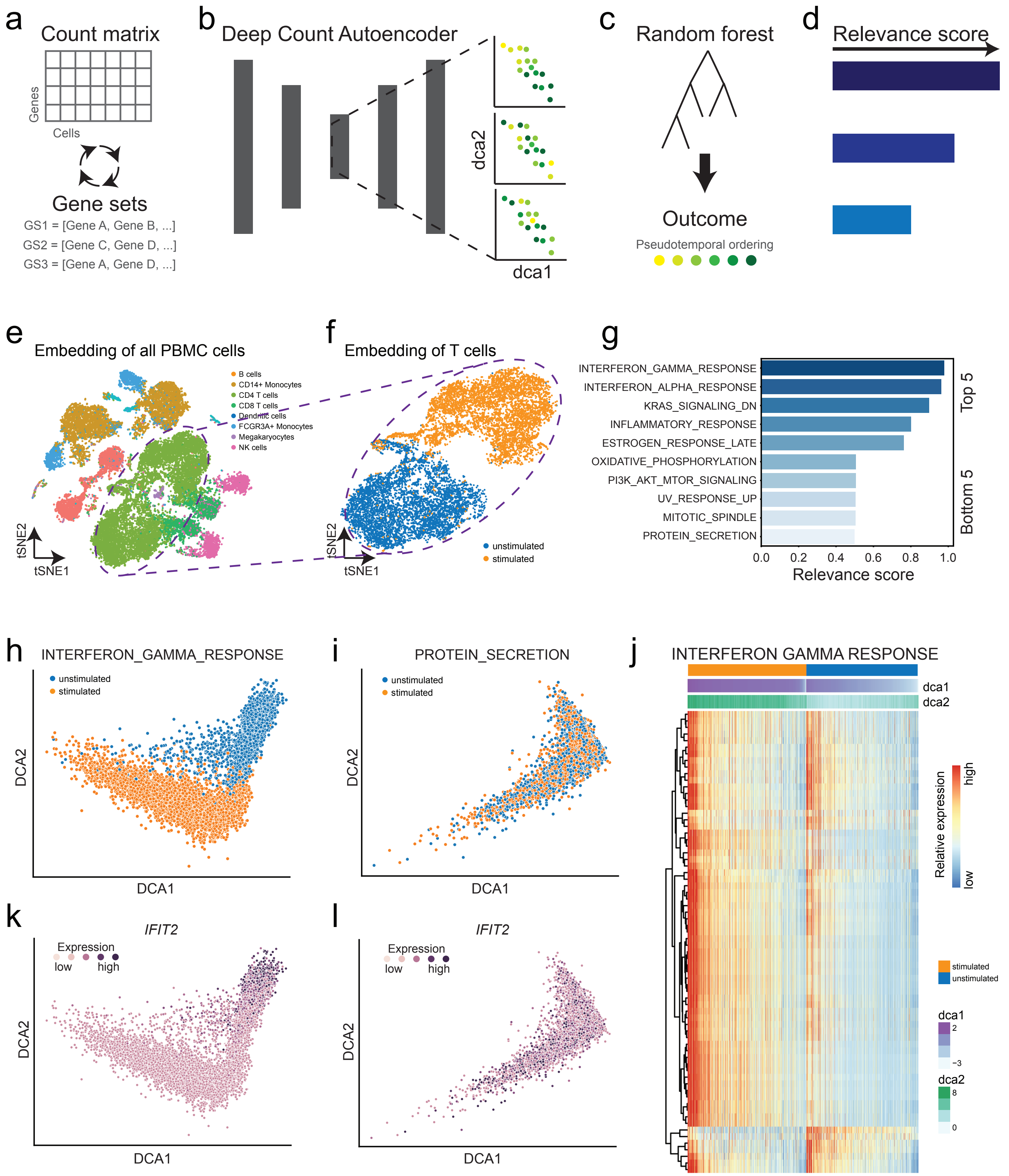

Figure 2 Click here to download Figure Figure2.pdf

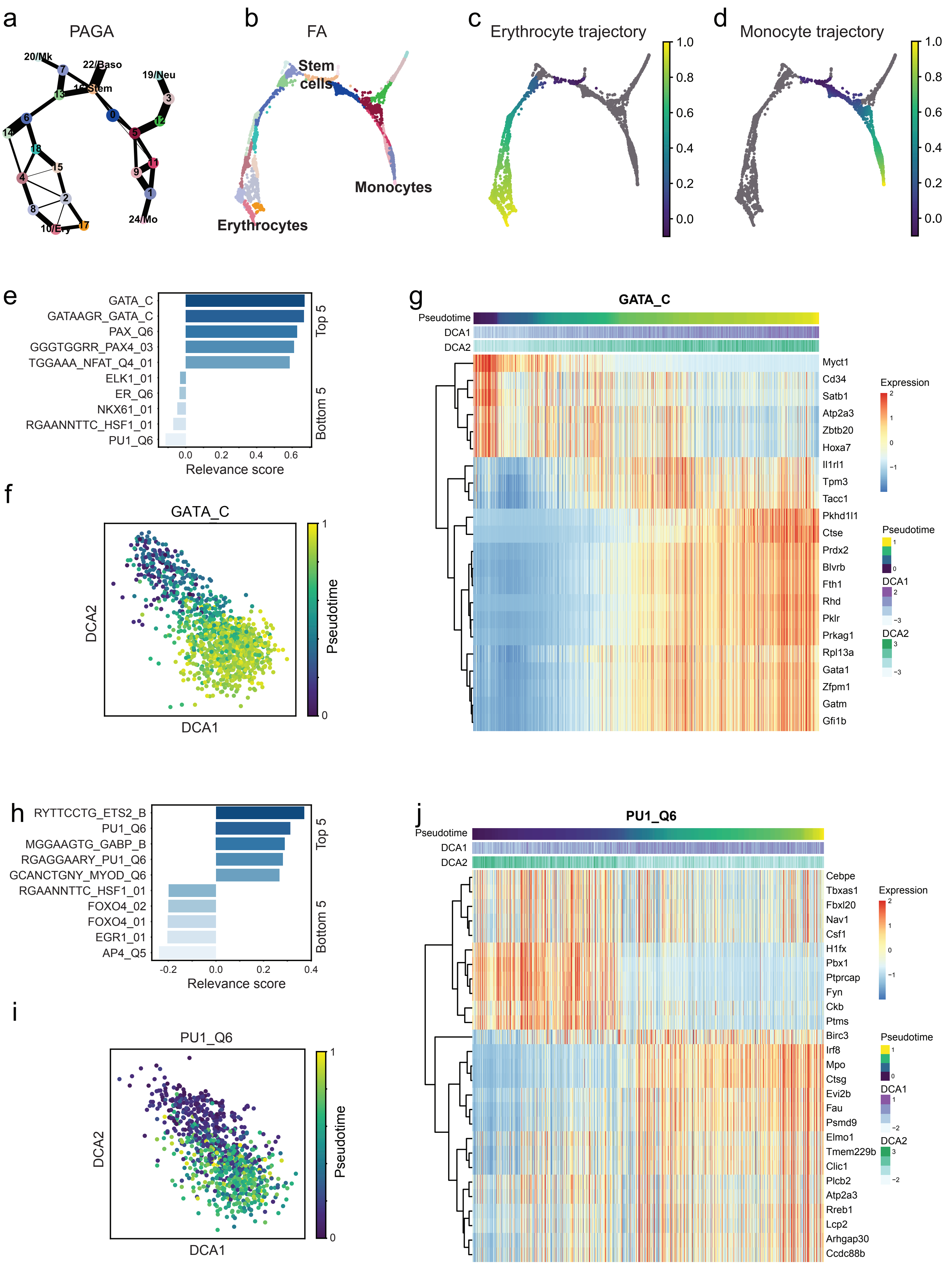

Supplemental material

Click here to access/download
Supplementary Material
Supplemental_material.pdf

 Click here to download Personal Cover DrivAER cover letter - Gigascience.docx

UTHealth
The University of Texas
Health Science Center at Houston

School of Biomedical Informatics

Zhongming Zhao, PhD
Chair Professor for Precision Health
Director, Center for Precision Medicine
Director, Cancer Genomics Core

February 5, 2020

Laura Goodman, PhD
Editor-in-Chief
Gigascience

Dear Dr. Goodman,

We are submitting a manuscript in the category Technical Note entitled "DrivAER: Identification of driving transcriptional programs in single cell RNA sequencing data" for your consideration for publication in *Gigascience*. In presubmission communication, Executive Editor Dr. Scott Edmunds suggested that this manuscript may be appropriate for *Gigascience*.

Our manuscript describes DrivAER, a novel machine learning approach. DrivAER is based on a novel concept which we termed "relevance scores". Similar to gene set enrichment analyses, relevance scores quantify the association between the expression patterns of sets of genes and specified outcomes of interest, such as disease status or pseudotemporal ordering. In our manuscript, we demonstrate that DrivAER correctly extracts known regulators from complex scRNAseq data sets and thereby significantly improves our understanding of the underlying molecular mechanisms.

DrivAER, including interactive usage tutorial, is freely available via Github (github.com/lkmklsmn/DrivAER) and smoothly integrates downstream of the popular scRNA-seq analysis framework Scanpy.

Our manuscript has not been published in any other journal and is not under consideration for publication elsewhere, except on bioRXiv (https://www.biorxiv.org/content/10.1101/864165v1), where we have received numerous tweets and other comments. We have no conflicts of interest to disclose.

We believe that our manuscript is appropriate for publication by *Gigascience* because it aligns well with other scRNA-seq methods published in your journal (IKAP, rCASC, ascend, etc). As scRNA-seq is rapidly evolving into a main technique in biological and biomedical research, we believe that our tool will be widely used by the scRNA-seq community.

We hope you agree that our manuscript can be of high interest to the readers of *Gigascience*. To facilitate the review process, a list of knowledgeable reviewers is attached along with their contact information. If you have any questions about this manuscript, please feel free to call or write.

Thank you for your consideration!

Zhongming & Lukas

Zhongming Zhao, PhD
Chair Professor for Precision Health

7000 Fannin St, Suite 600, Houston, Texas 77030. Tel. 713.500.3631. Email: zhongming.zhao@uth.tmc.edu

Professor of Biomedical Informatics and Human Genetics
School of Biomedical Informatics and School of Public Health
Adjunct Professor, Department of Psychiatry and Behavioral Sciences
UTHealth McGovern Medical School
Founding Director, Center for Precision Health
Director, UTHealth Cancer Genomics Core
University of Texas Health Science Center at Houston (UTHealth)

Lukas Simon, PhD
Assistant Professor
Center for Precision Health, School of Biomedical Informatics
University of Texas Health Science Center at Houston (UTHealth)

Suggested referees:

Wei Chen, PhD
Associate Professor of Pediatrics, Department of Pediatrics, School of Medicine
Associate Professor, Biostatistics, Human Genetics
University of Pittsburgh
Phone: 412-692-6241
E-mail: wec47@pitt.edu

Jiang Qian, PhD
Professor
The Wilmer Eye Institute
The Johns Hopkins University, School of Medicine
Phone: (443) 287-3882
Email: jiang.qian@jhmi.edu

Steve Horvath, PhD
Professor of Human Genetics & Biostatistics
Department of Biostatistics
UCLA School of Public Health
Phone: (310) 825-9299
E-mail: SHorvath@mednet.ucla.edu

Zhaolei Zhang, PhD
Professor
The Donnelly Centre
University of Toronto
Phone: 416-946-0924
Email: zhaolei.zhang@utoronto.ca

Barbara Treutlein, PhD
Professor
Department of Biosystems Science and Engineering
ETH Zurich
Phone: + 41 61 387 40 43
Email: barbara.treutlein@bsse.ethz.ch
