# Supplementary material for: DrivAER: Identification of driving transcriptional programs in single-cell RNA sequencing data
Source: Gigascience. 2020 Dec 10;9(12):giaa122. doi: 10.1093/gigascience/giaa122 (PMC7727875; doi:10.1093/gigascience/giaa122)
Supplement: giaa122_GIGA-D-20-00038_Revision_3 [file giaa122_giga-d-20-00038_revision_3.pdf]

# GigaScience


## DrivAER: Identification of driving transcriptional programs in single-cell RNA sequencing data

--Manuscript Draft--

| | |
|---|---|
| **Manuscript Number:** | GIGA-D-20-00038R3 |
| **Full Title:** | DrivAER: Identification of driving transcriptional programs in single-cell RNA sequencing data |
| **Article Type:** | Technical Note |
| **Funding Information:** | Cancer Prevention and Research Institute of Texas (RP180734) — Prof Lukas Simon, Prof Zhongming Zhao |
| **Abstract:** | Background<br><br>Single-cell RNA sequencing (scRNA-seq) unfolds complex transcriptomic data sets into detailed cellular maps. Despite recent success, there is a pressing need for specialized methods tailored towards the functional interpretation of these cellular maps.<br><br>Findings<br><br>Here, we present DrivAER, a machine learning approach for the identification of Driving transcriptional programs using AutoEncoder based Relevance scores. DrivAER scores annotated gene sets based on their relevance to user-specified outcomes such as pseudotemporal ordering or disease status. DrivAER iteratively evaluates the information content of each gene set with respect to the outcome variable using autoencoders. We benchmark our method using extensive simulation analysis as well as comparison to existing methods for functional interpretation of scRNA-seq data. Furthermore, we demonstrate that DrivAER extracts key pathways and transcription factors that regulate complex biological processes from scRNA-seq data.<br><br>Conclusions<br><br>By quantifying the relevance of annotated gene sets with respect to specified outcome variables, DrivAER greatly enhances our ability to understand the underlying molecular mechanisms. |
| **Corresponding Author:** | Lukas Simon<br>University of Texas Health Science Center at Houston<br>Houston, UNITED STATES |
| **Corresponding Author Secondary Information:** | |
| **Corresponding Author's Institution:** | University of Texas Health Science Center at Houston |
| **Corresponding Author's Secondary Institution:** | |
| **First Author:** | Lukas Simon |
| **First Author Secondary Information:** | |
| **Order of Authors:** | Lukas Simon |
| | Fangfang Yan |
| | Zhongming Zhao |
| **Order of Authors Secondary Information:** | |
| **Response to Reviewers:** | We would like to thank the editor and reviewers for their input. |
| **Additional Information:** | |


*Powered by Editorial Manager® and ProduXion Manager® from Aries Systems Corporation*

| Question | Response |
| --- | --- |
| Are you submitting this manuscript to a special series or article collection? | No |
| **Experimental design and statistics**<br><br>Full details of the experimental design and statistical methods used should be given in the Methods section, as detailed in our Minimum Standards Reporting Checklist. Information essential to interpreting the data presented should be made available in the figure legends.<br><br>Have you included all the information requested in your manuscript? | Yes |
| **Resources**<br><br>A description of all resources used, including antibodies, cell lines, animals and software tools, with enough information to allow them to be uniquely identified, should be included in the Methods section. Authors are strongly encouraged to cite Research Resource Identifiers (RRIDs) for antibodies, model organisms and tools, where possible.<br><br>Have you included the information requested as detailed in our Minimum Standards Reporting Checklist? | Yes |
| **Availability of data and materials**<br><br>All datasets and code on which the conclusions of the paper rely must be either included in your submission or deposited in publicly available repositories (where available and ethically appropriate), referencing such data using a unique identifier in the references and in the "Availability of Data and Materials" section of your manuscript. | Yes |

| | |
|---|---|
| Have you have met the above requirement as detailed in our Minimum Standards Reporting Checklist? | |

Manuscript

Click here to access/download;Manuscript;DrivAER- revision 3.pdf

# DrivAER: Identification of driving transcriptional programs in single-cell RNA sequencing data

Lukas M. Simon[1*+], Fangfang Yan[1], Zhongming Zhao[1,2,3,4*]

1. Center for Precision Health, School of Biomedical Informatics, The University of Texas Health Science Center at Houston, Houston, TX 77030, USA
2. Human Genetics Center, School of Public Health, The University of Texas Health Science Center at Houston, Houston, TX 77030, USA
3. MD Anderson Cancer Center UTHealth Graduate School of Biomedical Sciences, Houston, TX 77030, USA
4. Department of Biomedical Informatics, Vanderbilt University Medical Center, Nashville, TN 37203, USA

ORCID IDs: LMS: 0000-0001-6148-8861, FY: 0000-0001-6231-5225, ZZ: 0000-0002-3477-0914

* To whom correspondence should be addressed: Lukas M. Simon (lukas.simon@bcm.edu) and Zhongming Zhao (zhongming.zhao@uth.tmc.edu)

+ Contact Lead

# Abstract

Background: Single-cell RNA sequencing (scRNA-seq) unfolds complex transcriptomic data sets into detailed cellular maps. Despite recent success, there is a pressing need for specialized methods tailored towards the functional interpretation of these cellular maps.

Findings: Here, we present DrivAER, a machine learning approach for the identification of Driving transcriptional programs using AutoEncoder based Relevance scores. DrivAER scores annotated gene sets based on their relevance to user-specified outcomes such as pseudotemporal ordering or disease status. DrivAER iteratively evaluates the information content of each gene set with respect to the outcome variable using autoencoders. We benchmark our method using extensive simulation analysis as well as comparison to existing methods for functional interpretation of scRNA-seq data. Furthermore, we demonstrate that DrivAER extracts key pathways and transcription factors that regulate complex biological processes from scRNA-seq data.

Conclusions: By quantifying the relevance of annotated gene sets with respect to specified outcome variables, DrivAER greatly enhances our ability to understand the underlying molecular mechanisms.

## Keywords

Autoencoder, machine learning, manifold interpretation, single-cell RNA sequencing, transcription factor

# Findings

## Background

Single cell RNA sequencing (scRNA-seq) experiments dissect biological processes or complex tissues at the cellular and molecular levels [1,2]. Due to the high complexity and large number of observations, one critical step in scRNA-seq analysis is dimension reduction [3]. Dimension reduction projects the high dimensional expression matrix into a low dimensional space, also called data manifold or cellular map, which captures the underlying biological processes [4]. A number of methods have been used for manifold learning in scRNA-seq data [5–10].

Biological meaning can be extracted from the data manifold following in-depth analysis. After cells are stratified into separate groups or along a continuum, differential expression analysis is performed. Gene set enrichment analysis represents one of the most popular approaches to interpreting lists of differentially expressed genes and has been frequently used on bulk RNA-seq data [11–13]. More recent work has adapted this approach to scRNA-seq data [14]. Additional tools for biological interpretation of scRNA-seq data focus on the identification of latent variation that is aligned with gene sets [15–18].

However, choosing the best parameters to identify differentially expressed genes across diverse scRNA-seq datasets is still an open challenge [19]. Moreover, subtle transcriptional signals driven by a specific set of genes may not be sufficiently reflected in the global data manifold. Therefore, there is a need for methods that facilitate biological interpretation without performing differential expression analysis that capture subtle transcriptional signals driven by knowledge-based annotated gene sets.

Here, we present DrivAER, a method for the identification of Driving transcriptional programs based on AutoEncoder derived Relevance scores. Transcriptional programs (TPs) are sets of genes sharing biological properties [20] such as genes sharing transcription factor binding motifs or genes involved in the same biological pathway [11,21]. TPs have been annotated extensively and DrivAER infers TP relevance scores for existing gene set annotations with respect to specified outcomes of interest. These outcomes can represent extrinsic phenotypes, such as disease status, or intrinsic phenotypes derived from the data itself, such as pseudotemporal trajectories. Relevance scores allow researchers to rank TPs and help explain the underlying molecular mechanisms.

We evaluated DrivAER by application to two publicly available scRNA-seq datasets and comparison to two competing methods called VISION [22] and PAGODA [15]. Our results demonstrate that DrivAER correctly extracts well-known regulators from complex scRNA-seq datasets profiling interferon stimulation and blood development. Moreover, DrivAER outperforms existing methods when subtle transcriptional signals are present. Our user-friendly tool integrates smoothly downstream of the popular scRNA-seq analysis framework Scanpy [23].

# Results

## DrivAER correctly identifies interferon response

DrivAER is based on one assumption: the data manifold of relevant TPs shares information with the outcome of interest. Irrelevant TPs, on the other hand, will generate data manifolds where the cells fall randomly with respect to the outcome of interest. DrivAER builds upon our Deep Count Autoencoder (DCA) method [9], which has been shown to achieve high scalability for large scRNA-seq data [24]. DrivAER iteratively applies DCA to the raw counts of

each annotated TP-specific gene set to generate a two-dimensional data manifold in an unsupervised manner (Fig. 1ab). Next, we associate the resulting manifold coordinates with the outcome of interest using random forest models (Fig. 1c). We interpret the random forest accuracy as relevance score, which quantifies the amount of information that is shared between the TP-specific data manifold and the outcome of interest (Fig. 1d).

To demonstrate the ability of DrivAER to perform correct manifold interpretation, we reanalyzed two publicly available scRNA-seq data sets. The first data set by Kang et al [25] described a transcriptional response to interferon stimulation (Fig. 1e). As a proof of principle, we asked if DrivAER could recapitulate this biology and extract the interferon signature as the driving transcriptional program defining the T cell data manifold (Fig. 1f). We applied DrivAER to the subset of T cells and evaluated all 50 Hallmark gene sets from MolSigDB [26] with respect to interferon stimulation (Table S1). Indeed, the “INTERFERON_GAMMA_RESPONSE” gene set received the highest relevance score (Fig. 1g) out of all 50 gene sets included in the analysis. Visualization of the T cell DCA embedding derived from the “INTERFERON_GAMMA_RESPONSE” gene set showed clear separation by condition (Fig. 1h), implicating that this gene set is the main driving force separating the stimulated and unstimulated T cells. As a negative control, we show the DCA embedding for one of the lowest scoring gene sets “PROTEIN_SECRETION” (Fig. 1i). For this gene set, the cells cluster randomly with respect to the stimulation status. The heatmap in Figure 1j shows the expression levels of T cells for the “INTERFERON_GAMMA_RESPONSE” gene set. It is important to note that the cells (columns) are ordered by stimulation status and that the DCA coordinates are strongly associated with the stimulation status. Most “INTERFERON_GAMMA_RESPONSE” genes are upregulated in stimulated compared to unstimulated cells. Expression of genes in the “PROTEIN_SECRETION” gene set shows a random pattern (Fig. S1).

To further manifest the biological meaning of the DCA embedding, we visualized the expression of interferon marker *IFIT2* in the “INTERFERON_GAMMA_RESPONSE” (Fig. 1k) and “PROTEIN_SECRETION” (Fig. 1l) embeddings. Expression levels of *IFIT2* increase along the DCA coordinates in the “INTERFERON_GAMMA_RESPONSE” derived embedding. In contrast, *IFIT2* expression is distributed randomly in the “PROTEIN_SECRETION” derived embedding. Therefore, DrivAER correctly identified the TPs driving interferon stimulation out of the entire collection of Hallmark gene sets.

## DrivAER unveils key transcription factors in blood development

Next, we tested whether DrivAER is capable of extracting key transcription factors (TF) involved in differentiation trajectories. DrivAER is particularly well suited to infer the relevance of TFs for the following reasons. TF mediated regulation is regarded as a combinatorial process which requires the coordination of multiple TFs and co-activators [27]. Moreover, there are vast differences in sensitivity and typical sequencing depth across various scRNA-seq technologies. Due to the low RNA capture rate in some scRNA-seq technologies, generally lowly expressed TFs may not be detected reliably [28]. Therefore, the expression levels of the target genes represent a better proxy of TF activity compared to the expression level of the TF itself [29].

To demonstrate the utility of DrivAER, we use a collection of TF-target annotations to infer TF activity and reanalyzed a hematopoietic differentiation dataset by Paul et al [30]. The authors identified and described the main blood development trajectories including differentiation from stem cells towards erythrocytes and monocytes (Fig. 2ab). Next, we calculated two independent pseudotemporal trajectories for erythrocyte and monocyte differentiation (Fig. 2cd). We then applied DrivAER to identify TFs that are relevant for erythrocyte and monocyte differentiation using the entire collection of motif gene sets contained in MolSigDB [31]. Among all 495 gene sets included in the analysis, DrivAER identified the

GATA TF family as the most relevant in the erythrocyte trajectory (Fig. 2e, Table S1). The DCA embedding derived from the "GATA_C" gene set showed strong clustering by pseudotime, demonstrating that GATA target gene expression is highly coordinated along this trajectory (Fig. 2f). Indeed, expression levels of GATA targets showed strong association with both pseudotime and DCA coordinates (Fig. 2g).

Of note, targets showed both up and down-regulation. A fraction of targets increased in expression along the trajectory while a smaller fraction decreased. When integrating annotation from the TTRUST database [32] with the "GATAAGR_GATA_C" gene set, *Fli1* expression was predicted to be repressed by TF GATA1 and, correspondingly, we observed a negative correlation along the trajectory between these two genes (Fig. S2).

Among the most relevant TFs in the monocyte trajectory was PU1 (Fig. 2h, Table S1), which also showed strong association between the DCA embedding (Fig. 2j) and target gene expression (Fig. 2i) with pseudotime. Both GATA and PU1 are well-known lineage determining regulators in blood development, with GATA and PU1 driving erythrocyte and monocyte differentiation, respectively [33]. However, such conclusions cannot be drawn based on the expression of *Gata1* and *Pu1* itself. Although *Gata1* and *Pu1* showed increased expression along their developmental trajectories, many other TFs exhibited a similar pattern, making it difficult to pinpoint the driving regulator (Fig. S3). Taken together, our findings demonstrate that DrivAER robustly explains the molecular mechanisms underlying complex biological processes.

## Benchmarking DrivAER

To further assess and thoroughly benchmark our method in a controlled setting, we performed extensive simulation analysis. We used the Splatter [34] framework to simulate scRNA-seq data consisting of two groups of cells with subtle transcriptional differences where only 10% of genes were differentially expressed between the two groups. Subsequently, we

generated different gene sets which varied in the number of truly differentially expressed (DE) genes (Fig. 3a). The gene sets ranged from sets without any truly DE genes (DE fraction = 0) to sets consisting of all truly DE genes (DE fraction = 1). Visualization of all genes in reduced dimensions using UMAP showed no clear separation between the two cell groups (Fig. 3b). However, dimension reduction restricted to truly DE genes using DCA showed separation between the cells (Fig. 3c), indicating that while the signal may be weak across all genes, targeted dimension reduction of specific genes successfully recovered the underlying cellular manifold.

To evaluate methodological aspects underlying DrivAER, we performed the following analyses. With respect to the dimension reduction task, we compared DCA with principal component analysis (PCA), Uniform Manifold Approximation and Projection (UMAP) and t-distributed stochastic neighbor embedding (tSNE). Across the gene sets that vary in the fraction of truly DE genes, DCA overall achieved the highest relevance scores (Fig. 3d). At low fractions of DE genes, the alternative dimension reduction methods slightly outperformed DCA (Fig. S5). Therefore, we implemented PCA, UMAP and tSNE based dimension reduction into the DrivAER framework. Users have the option to select any of these four dimension reduction methods for their DrivAER analysis.

Next, we compared random forest and support vector machines for the classification task. We did not observe any significant differences in performance between these two methods, indicating that random forest models represent an appropriate choice for this task (Fig. 3e). Moreover, we evaluated the impact of various hidden layer configurations during the DCA dimension reduction underlying DrivAER. We applied DrivAER using varying bottleneck layer sizes to the collection of simulated gene sets. The performance did not differ substantially across the three configurations, indicating that DrivAER is robust to various hidden layer

configurations (Fig. 3f). Even when the gene set contained only 20% of truly DE genes, the relevance score was significantly higher than that of random gene sets over 10 bootstraps (One-sided t-test, $P < 0.05$, Fig. 3g), demonstrating DrivAER's ability to capture subtle transcriptional signals.

Additionally, we evaluated the different bottleneck configurations in a more complex simulation scenario consisting of four unbalanced groups of cells. All three configurations successfully recovered the varying degree of signal in the gene sets. The four- and eight-dimensional bottleneck layers outperformed the two-dimensional bottleneck layer slightly (Fig. S6a). Visualization of the cellular manifold derived from the two-, four- and eight-dimensional bottleneck layers, showed improved separation of the four cell groups, suggesting that higher dimensional bottleneck layers may be needed to resolve more complex data manifolds (Fig. S6c-d).

Next, we compared DrivAER to VISION [22] and PAGODA [15], two existing tools for the functional interpretation of scRNA-seq data. Unlike DrivAER, VISION does not iteratively subject gene sets to dimension reduction; instead it operates directly on the global cellular manifold. VISION uses a local autocorrelation statistic to infer the relevance of various gene sets. PAGODA, on the other hand, calculates the adjusted z score for each gene set and assesses the variance explained for significance. Both the autocorrelation and adjusted z scores are analogous to the DrivAER relevance score. High values indicate relevant gene sets.

We applied VISION in directed and undirected mode as well as PAGODA to the simulated gene sets. As expected, for all three methods, the respective scores increased with the fraction of truly DE genes (Fig. 3h-j). However, it is important to note the following differences. For PAGODA, the gene sets with 20% truly DE genes achieved a lower adjusted z score compared to the completely random gene sets. Moreover, the fact that none of the

absolute adjusted z scores passed 1.96 implied that none of the gene sets achieved statistical significance. For VISION, we observed a similar pattern. While the autocorrelation statistic increased with the fraction of truly DE genes, it never passed 0.1 and 100% truly DE gene sets never reached a high Autocorrelation. Since VISION operates on the global manifold (i.e. Figure 3b) instead of gene set specific manifolds (i.e. Figure 3c), it is less likely to capture subtle transcriptional differences. When using DrivAER, on the other hand, random gene sets achieved a relevance score around 0.5. This corresponds to the likelihood of taking a random guess with two classes. Correspondingly, gene sets consisting of all truly DE genes approached relevance scores close to 1. Therefore, relevance scores for categorical phenotype can be readily interpreted.

For additional comparison, we applied VISION and PAGODA to the interferon stimulation and blood development data sets (Fig. S4). All three methods clearly identified the correct TPs involved in interferon stimulation. In the erythrocyte trajectory, the GATA_C gene set achieved high scores using DrivAER and VISION but not PAGODA. For the monocyte trajectory, only DrivAER generated high relevance scores for PU1 related gene sets.

## Discussion and conclusions

While autoencoders have been applied for unsupervised dimension reduction in bulk [35–37] and scRNA-seq data [38,39], DrivAER makes use of autoencoders with a different goal. By iterative application, DrivAER scores gene sets based on their relevance instead of trying to identify potential signatures that may not be captured in databases. Thus, while using autoencoders for unsupervised dimension reduction intrinsically, our method aims to rank gene sets in a supervised fashion.

Unlike VISION, DrivAER does not require a pre-defined distinction between the sign of regulation (repression or activation) of genes in a given gene set. The unsupervised nature of the DCA embedding captures any form of non-random, coordinated expression pattern. Therefore, DrivAER captures complex, non-linear expression patterns commonly observed in scRNA-seq data. An additional benefit of DrivAER is its ability to visualize the gene set specific data manifold. These visualizations promote discovery of transcriptional regulation that may otherwise be hidden in the summary statistics generated by other methods including gene set enrichment analysis or VISION and PAGODA. Moreover, as demonstrated in the simulation analysis, DrivAER's relevance score is readily interpretable.

As illustrated in the blood development example, we divided the manifold into independent trajectories for interpretation. However, DrivAER provides the flexibility to be applied to the entire manifold or any subset of it. The user can make this choice and arbitrarily define regions of the manifold, which are expected to be regulated by a TP.

Additionally, as demonstrated in the blood development example, DrivAER enables users to make inferences about regulators that were not measured or where measurements are noisy. We envision that users will apply DrivAER to infer activity of regulators not generally detected in scRNA-seq data such as microRNAs and long noncoding RNAs.

In the current approach DCA needs to be retrained for each gene set because the input genes and thus the network architecture changes between gene sets. Therefore, the running time of DrivAER depends on the number of gene sets included in the analysis. In the interferon stimulation analysis, the running time per gene sets averages between 20-30 seconds depending on the number of genes and convergence of the model. To improve speed, we plan to extend DrivAER by developing a “hot-start” approach in future work.

In summary, specialized methods facilitating the functional interpretation of scRNA-seq data are needed to fuel the rapid progress in the field. DrivAER is a novel machine learning approach that is effective for manifold interpretation in scRNA-seq data. Our results demonstrate that relevance scores represent a useful measure to extract driving transcriptional regulators from complex scRNA-seq data sets. DrivAER, including interactive usage tutorial, is freely available from Github (https://github.com/lkmklsmn/DrivAER) and we anticipate broad usage by the community.

# Methods

## Transcriptional program annotations

The Molecular Signatures Database (MolSigDB , v7.0) was used to define transcriptional programs [11]. The Hallmark gene set contained 50 gene sets corresponding to specific well-defined biological processes [26]. The C3 transcription factor targets collection contains 610 genes sets in total, where genes share the same *cis*-regulatory motifs from known TF binding sites in the TRANSFAC (v7.4) [40] database around their transcription start sites [31]. The gene sets with motifs not included in the TRANSFAC database were removed. A total of 495 gene sets were utilized in the blood development study. For mouse scRNA-seq datasets, the gene symbols were converted to mouse homologs before running DrivAER.

## DrivAER

DrivAER was written in Python and designed to integrate downstream of Scanpy [23]. Given a collection of annotated gene sets, DrivAER uses the Deep Count Autoencoder (DCA) [9] to calculate a two-dimensional data manifold for each gene set. Autoencoders are neural

networks that learn an efficient compression of high dimensional data [41]. One important characteristic that distinguishes DCA from other dimension reduction methods is a scRNA-seq specific noise model. The bottleneck layer captures the compression and represents the data manifold. As default for DrivAER, we set the bottleneck dimension to two neurons. DCA takes a raw count matrix as input and outputs the data manifold coordinates using the parameter mode = “latent”. To account for differences in library size, size factors derived from the transcriptome-wide, instead of gene set specific, expression matrix are being fed into DCA.

The relevance scores are derived using random forest models as implemented in the Python module sklearn (v0.21.2). Once DCA has reduced the dimensions, the two-dimensional data manifold coordinates are used as input features and the variable of interest as outcome in the random forest model. For categorical outcomes, “*sklearn.ensemble.RandomForestClassifier*” is used. For continuous outcomes, such as pseudotemporal trajectories, “*sklearn.ensemble.RandomForestRegressor*” is used. The number of trees was set to 500. The Out-of-Bag accuracy score of the TP-specific random forest model represents the relevance score.

For the benchmarking purposes only, we applied support vector machine classification as implemented in the R package e1071 with default parameters. Additionally, we implemented three alternative dimension reduction methods into the DrivAER framework. PCA, tSNE and UMAP were implemented using the Scanpy functions “*pp.pca*”, “*tl.tsne*” and “*tl.umap*”, respectively. All functions use Scanpy’s default parameters.

## Simulation analysis

scRNA-seq data was simulated using the splatter R package [34]. Specifically, the splatSimulate() function was used to simulate scRNA-seq data with two equally sized groups,

consisting of 500 genes and 2000 cells. The default gene expression and library size parameters were used. To simulate subtle transcriptional differences, the proportion of differentially expressed genes was set to 0.1 and the differential expression factor was set to 0.01. To include specific noise commonly encountered in scRNA-seq data, the dropout type was set to “experiment”. The “dropout.mid” parameter was set to 5 and “dropout.shape” was set to -1. The splatSimulate() function was also used to simulate scRNA-seq data with four unbalanced groups, containing 1000 genes and 4000 cells. The proportion of cell numbers in these four groups was set to 0.1, 0.2, 0.3, and 0.4, respectively. The “dropout.mid” parameter was set to 2 and “dropout.shape” was set to -1.

To simulate gene sets from a continuous spectrum of relevance the following approach was used. Gene sets were created by combining truly differentially expressed genes with genes showing no expression difference between the two groups. We generated gene sets containing six different fractions of truly DE gene sets (0, 0.2, 0.4, 0.6, 0.8, 1). Ten bootstrap samples were generated at each fraction. These 60 simulated gene sets were used for DrivAER evaluation.

For the evaluation of DrivAER using different configurations of hidden layers five bootstrap samples were generated at each fraction of truly DE gene sets. These 30 simulated gene sets were subjected to three different configurations of hidden layers (4, 2, 4), (8, 4, 8) and (16, 8, 16), in an independent analysis.

## Interferon stimulation analysis

The scRNA-seq data set of 29,065 PBMCs from lupus patients with and without interferon stimulation were obtained from the GEO database (GSE96583). The tSNE coordinates as well as cell type and state (stimulated or unstimulated) information displayed in Figure 1e and f were taken from the Supplemental materials of the original publication. CD4 T

cells were isolated based on the cell type annotation file from the paper and DBSCAN clustering algorithm [42] was utilized to remove outlier cells (epsilon = 0.1, min_cells = 20). Before applying DrivAER, lowly expressed genes with less than 3 counts across all cells were filtered out.

## Blood development analysis

Expression data for the Paul et al data was obtained from Scanpy's (version 1.4.6) [23] built-in datasets using the "*scanpy.datasets.paul15()"* function. Expression data consists of 2730 hematopoietic stem cells and 3451 genes. The preprocessing of the data was performed following the Scanpy tutorial using "*scanpy.pp.recipe_zheng17()*" function. Specifically, the 1000 most highly variable genes were selected for downstream analysis. Louvain clustering (version 0.6.1) was conducted with resolution of 1, which resulted in 25 clusters. Clusters were annotated based on the expression of canonical cell type marker genes. Two major developmental trajectories were identified, namely the differentiation of hematopoietic stem cells to erythrocytes and monocytes. Pseudotemporal ordering was independently calculated for these two trajectories using the "*scanpy.tl.dpt"* function. DrivAER was applied to the raw counts and pseudotemporal ordering of each trajectory independently to infer relevant TPs.

Expression data for the Nestorowa data set was obtained from the "Gene and protein expression in adult hematopoiesis" website (http://blood.stemcells.cam.ac.uk/single_cell_atlas). Based on the provided annotation, cells were divided into the erythrocyte and monocyte trajectory. Pseudotemporal ordering was calculated as described above. DrivAER was applied as described above.

## PAGODA

PAGODA facilitates biological interpretation by testing gene sets for coordinated variability amongst cells. Briefly, PAGODA first estimates measurement properties, such as sequencing depth, drop-out rate and amplification noise for each cell. Next, PAGODA renormalizes the expression variance of each gene accounting for the measurement properties. Next, PAGODA tests whether a panel of genes sets shows statistically significant excess of coordinated variability using weighted principal components analysis. A high dispersion or adjusted z-score indicates statistically significance and transcriptional heterogeneity of the gene set. The underlying idea is that overdispersed gene sets separate cells along a certain principal component. The separation of cells along this gene set specific principal component implies relevance of the gene set.

The SCDE (version 1.99.1) R package including PAGODA was downloaded from github (http://hms-dbmi.github.io/scde/package.html). The geneset overdispersion analysis was conducted following the PAGODA tutorial with default parameters. The minimum number of reads for a gene was set to 2. The pagoda.varnorm() function was used to normalize the variance. The custom gene set environment file was created using the 60 simulated gene sets described above. The pagoda.pathway.wPCA() and pagoda.top.aspects() function were used to estimate the overdispersion of each gene set. The adjusted z-score was compared to DrivAER's relevance scores.

## VISION

VISION annotates sources of variation in scRNA-seq data by directly operating on the global cellular manifold. For each cell, VISION first identifies its closest K -nearest neighbor

(KNN) graph. By default, VISION uses PCA to create this low dimensional space, but the users can provide more advanced latent space models. Next, VISION calculates a signature score for each annotated gene set and subsequently assesses whether the signature score is randomly distributed throughout the cellular manifold using a local autocorrelation statistic, the Geary's C [43]. High values of VISION's autocorrelation indicate non-random pattern and this score can be compared to DrivAER's relevance score. The input of VISION is the normalized count matrix and the signature files or objects containing various gene sets. The output is a VISION object containing the local autocorrelation scores for each gene set and corresponding embedding plot colored by scores.

VISION (version 2.0.0) was downloaded from github (https://github.com/yoseflab/VISION). We applied VISION to the simulation analysis, interferon stimulation and blood development experiments using default parameters. For the simulation analysis, VISION was run in both directed and undirected mode. The signature object was created using the 60 simulated gene sets described above. For the undirected mode, 1 was used for the value of every gene. For the directed mode, the values for the up and down-regulated genes were set to 1 and -1, respectively. For the blood development experiments, VISION was run in trajectory mode following the pipeline of the VISION tutorial. After filtering and normalization, slingshot from the Dynverse package (version 0.1.1) [44] https://github.com/dynverse/dyno) was used to infer the trajectory. The VISION scores for each gene set were compared to DrivAER's relevance scores.

## Abbreviations

DCA: deep count autoencoder; DE: differentially expressed; MolSigDB: the Molecular Signatures Database; PCA: principal component analysis; scRNA-seq: single cell RNA

sequencing; TF: transcription factor; TP: transcriptional program; tSNE: t-distributed Stochastic Neighbor Embedding.

# Declarations

## Ethics approval and consent to participate

Not applicable

## Consent for publication

Not applicable

## Availability of data and materials

An archival copy of the code and supporting data is available via the GigaScience repository, GigaDB [45].

Availability of supporting source code and requirements

Project name: DrivAER
Project home page: https://github.com/lkmklsmn/DrivAER
Operating system(s): Platform independent
Programming language: Python
License: MIT license
bio.tools ID: drivaer
Research Resource Identification Initiative ID: SCR_019076

Competing interests

The authors declare that they have no competing interests.

## Funding

ZZ was partially supported by the National Institutes of Health [R01LM012806], Cancer Prevention and Research Institute of Texas [CPRIT RP180734], and The Chair Professorship for Precision Medicine Funds from the University of Texas Health Science Center at Houston. The funders had no role in the study design, data collection and analysis, decision to publish, or preparation of the manuscript.

## Authors' contributions

LS conceived the idea and designed the project. LS and FY analyzed the data. ZZ participated and supervised the project. LS, FY, ZZ wrote the manuscript. All authors read and approved the final manuscript.

## Acknowledgements

The authors would like to thank the members of the Bioinformatics and Systems Medicine Laboratory at the University of Texas Health Science Center at Houston for stimulating discussion.

# References


1. Trapnell C. Defining cell types and states with single-cell genomics. Genome Res. 2015;25:1491–8.

2. Hwang B, Lee JH, Bang D. Single-cell RNA sequencing technologies and bioinformatics pipelines [Internet]. Experimental & Molecular Medicine. 2018. Available from: http://dx.doi.org/10.1038/s12276-018-0071-8

3. Luecken MD, Theis FJ. Current best practices in single-cell RNA-seq analysis: a tutorial. Mol Syst Biol. 2019;15:e8746.

4. Moon KR, Stanley JS, Burkhardt D, van Dijk D, Wolf G, Krishnaswamy S. Manifold learning-based methods for analyzing single-cell RNA-sequencing data [Internet]. Current Opinion in Systems Biology. 2018. p. 36–46. Available from: http://dx.doi.org/10.1016/j.coisb.2017.12.008

5. Coifman RR, Lafon S, Lee AB, Maggioni M, Nadler B, Warner F, et al. Geometric diffusions as a tool for harmonic analysis and structure definition of data: diffusion maps. Proc Natl Acad Sci U S A. 2005;102:7426–31.

6. Haghverdi L, Buettner F, Theis FJ. Diffusion maps for high-dimensional single-cell analysis of differentiation data. Bioinformatics. 2015;31:2989–98.

7. van der Maaten L, Hinton G. Visualizing data using t-SNE. J Mach Learn Res. 2008;2579–605.

8. Lopez R, Regier J, Cole MB, Jordan MI, Yosef N. Deep generative modeling for single-cell transcriptomics. Nat Methods. 2018;15:1053–8.

9. Eraslan G, Simon LM, Mircea M, Mueller NS, Theis FJ. Single-cell RNA-seq denoising using a deep count autoencoder. Nat Commun. 2019;10:390.

10. McInnes L, Healy J, Saul N, Großberger L. UMAP: Uniform Manifold Approximation and Projection [Internet]. Journal of Open Source Software. 2018. p. 861. Available from: http://dx.doi.org/10.21105/joss.00861

11. Subramanian A, Tamayo P, Mootha VK, Mukherjee S, Ebert BL, Gillette MA, et al. Gene set enrichment analysis: a knowledge-based approach for interpreting genome-wide expression profiles. Proc Natl Acad Sci U S A. 2005;102:15545–50.

12. Wu D, Smyth GK. Camera: a competitive gene set test accounting for inter-gene correlation [Internet]. Nucleic Acids Research. 2012. p. e133–e133. Available from:

http://dx.doi.org/10.1093/nar/gks461

13. Goeman JJ, Bühlmann P. Analyzing gene expression data in terms of gene sets: methodological issues. Bioinformatics. 2007;23:980–7.

14. Finak G, McDavid A, Yajima M, Deng J, Gersuk V, Shalek AK, et al. MAST: a flexible statistical framework for assessing transcriptional changes and characterizing heterogeneity in single-cell RNA sequencing data. Genome Biol. 2015;16:278.

15. Fan J, Salathia N, Liu R, Kaeser GE, Yung YC, Herman JL, et al. Characterizing transcriptional heterogeneity through pathway and gene set overdispersion analysis. Nat Methods. 2016;13:241–4.

16. Buettner F, Pratanwanich N, McCarthy DJ, Marioni JC, Stegle O. f-scLVM: scalable and versatile factor analysis for single-cell RNA-seq. Genome Biol. 2017;18:212.

17. Martignetti L, Calzone L, Bonnet E, Barillot E, Zinovyev A. ROMA: Representation and Quantification of Module Activity from Target Expression Data. Front Genet. 2016;7:18.

18. Risso D, Perraudeau F, Gribkova S, Dudoit S, Vert J-P. A general and flexible method for signal extraction from single-cell RNA-seq data. Nat Commun. 2018;9:284.

19. Wang T, Li B, Nelson CE, Nabavi S. Comparative analysis of differential gene expression analysis tools for single-cell RNA sequencing data. BMC Bioinformatics. 2019;20:40.

20. Heimberg G, Bhatnagar R, El-Samad H, Thomson M. Low Dimensionality in Gene Expression Data Enables the Accurate Extraction of Transcriptional Programs from Shallow Sequencing. Cell Syst. 2016;2:239–50.

21. Kanehisa M. KEGG: Kyoto Encyclopedia of Genes and Genomes [Internet]. Nucleic Acids Research. 2000. p. 27–30. Available from: http://dx.doi.org/10.1093/nar/28.1.27

22. DeTomaso D, Jones MG, Subramaniam M, Ashuach T, Ye CJ, Yosef N. Functional interpretation of single cell similarity maps. Nat Commun. 2019;10:4376.

23. Wolf FA, Angerer P, Theis FJ. SCANPY: large-scale single-cell gene expression data analysis. Genome Biol. 2018;19:15.

24. Sun S, Zhu J, Ma Y, Zhou X. Accuracy, robustness and scalability of dimensionality reduction methods for single-cell RNA-seq analysis. Genome Biol. 2019;20:269.

25. Kang HM, Subramaniam M, Targ S, Nguyen M, Maliskova L, McCarthy E, et al. Multiplexed droplet single-cell RNA-sequencing using natural genetic variation. Nat Biotechnol. 2018;36:89–94.

26. Liberzon A, Birger C, Thorvaldsdóttir H, Ghandi M, Mesirov JP, Tamayo P. The Molecular Signatures Database Hallmark Gene Set Collection [Internet]. Cell Systems. 2015. p. 417–25. Available from: http://dx.doi.org/10.1016/j.cels.2015.12.004

27. Zhu Z, Shendure J, Church GM. Discovering functional transcription-factor combinations in

the human cell cycle. Genome Res. 2005;15:848–55.

28. Hicks SC, Townes FW, Teng M, Irizarry RA. Missing data and technical variability in single-cell RNA-sequencing experiments. Biostatistics. 2018;19:562–78.

29. Schacht T, Oswald M, Eils R, Eichmüller SB, König R. Estimating the activity of transcription factors by the effect on their target genes. Bioinformatics. 2014;30:i401–7.

30. Paul F, Arkin Y ’ara, Giladi A, Jaitin DA, Kenigsberg E, Keren-Shaul H, et al. Transcriptional Heterogeneity and Lineage Commitment in Myeloid Progenitors. Cell. 2016;164:325.

31. Xie X, Lu J, Kulbokas EJ, Golub TR, Mootha V, Lindblad-Toh K, et al. Systematic discovery of regulatory motifs in human promoters and 3′ UTRs by comparison of several mammals [Internet]. Nature. 2005. p. 338–45. Available from: http://dx.doi.org/10.1038/nature03441

32. Han H, Cho J-W, Lee S, Yun A, Kim H, Bae D, et al. TRRUST v2: an expanded reference database of human and mouse transcriptional regulatory interactions. Nucleic Acids Res. 2018;46:D380–6.

33. Monteiro R, Pouget C, Patient R. The gata1/pu.1 lineage fate paradigm varies between blood populations and is modulated by tif1γ. EMBO J. 2011;30:1093–103.

34. Zappia L, Phipson B, Oshlack A. Splatter: simulation of single-cell RNA sequencing data. Genome Biol. 2017;18:174.

35. Tan J, Ung M, Cheng C, Greene CS. Unsupervised feature construction and knowledge extraction from genome-wide assays of breast cancer with denoising autoencoders. Pac Symp Biocomput. 2015;132–43.

36. Chen L, Cai C, Chen V, Lu X. Learning a hierarchical representation of the yeast transcriptomic machinery using an autoencoder model. BMC Bioinformatics. 2016;17 Suppl 1:9.

37. Tan J, Doing G, Lewis KA, Price CE, Chen KM, Cady KC, et al. Unsupervised Extraction of Stable Expression Signatures from Public Compendia with an Ensemble of Neural Networks. Cell Syst. 2017;5:63–71.e6.

38. Geddes TA, Kim T, Nan L, Burchfield JG, Yang JYH, Tao D, et al. Autoencoder-based cluster ensembles for single-cell RNA-seq data analysis. BMC Bioinformatics. 2019;20:660.

39. Lin E, Mukherjee S, Kannan S. A deep adversarial variational autoencoder model for dimensionality reduction in single-cell RNA sequencing analysis. BMC Bioinformatics. 2020;21:64.

40. Matys V, Kel-Margoulis OV, Fricke E, Liebich I, Land S, Barre-Dirrie A, et al. TRANSFAC and its module TRANSCompel: transcriptional gene regulation in eukaryotes. Nucleic Acids Res. 2006;34:D108–10.

41. Hinton GE, Salakhutdinov RR. Reducing the dimensionality of data with neural networks. Science. 2006;313:504–7.

42. Louhichi S, Gzara M, Ben Abdallah H. A density based algorithm for discovering clusters

with varied density [Internet]. 2014 World Congress on Computer Applications and Information Systems (WCCAIS). 2014. Available from: http://dx.doi.org/10.1109/wccais.2014.6916622

43. Geary RC. The Contiguity Ratio and Statistical Mapping [Internet]. The Incorporated Statistician. 1954. p. 115. Available from: http://dx.doi.org/10.2307/2986645

44. Saelens W, Cannoodt R, Todorov H, Saeys Y. A comparison of single-cell trajectory inference methods. Nat Biotechnol. 2019;37:547–54.

45. Simon LM, Yan F, Zhao Z. Supporting data for “DrivAER: Identification of driving transcriptional programs in single-cell RNA sequencing data”. GigaScience Database. 2020. http://dx.doi.org/10.5524/100809

# Figure Legends

**Figure 1. DrivAER correctly identifies interferon response.** (a) DrivAER iteratively subjects annotated gene sets to unsupervised dimension reduction via Deep Count Autoencoder (DCA). (b) For each gene set, the two-dimensional data manifold coordinates are calculated and (c) subsequently used as input features in a random forest model to predict the outcome of interest (i.e. pseudotemporal ordering). (d) The random forest prediction accuracy represents the relevance score. (e) t-Distributed Stochastic Neighbor Embedding (tSNE) visualization displays all PBMC cells colored by cell type. (f) Cellular map (tSNE) of T cell subset clusters by stimulation status. (g) Barplot indicates relevance scores of the five most and least relevant transcription programs. DCA embeddings calculated based on “INTERFERON_GAMMA_RESPONSE” (h) and “PROTEIN_SECRETION” (i) (negative control) gene sets are depicted. Cells are colored by stimulation status. (j) Heatmap shows gene expression of “INTERFERON_GAMMA_RESPONSE” target genes and cells in rows and columns, respectively. Columns are ordered first by stimulation status and second by DCA coordinates. Bars on top of the heatmap represent stimulation status and DCA coordinates one

and two. Red and blue colors correspond to high and low relative expression values. Relative expression of interferon gene *IFIT2* is overlaid on top of the DCA embeddings derived from “INTERFERON_GAMMA_RESPONSE” (k) and “PROTEIN_SECRETION” (l) gene sets. Dark colors indicate higher expression.

**Figure 2. DrivAER unveils key transcription factors in blood development.** PAGA (a) and cell-level graph (b) visualization of the Paul et al data set. Cells are colored by Louvain clustering as provided by Scanpy. Two independent trajectories were calculated for erythrocyte (c) and monocyte (d) development. Cells are colored by pseudotime. (e) Barplot displays relevance scores for the five most and least relevant transcription factors in the erythrocyte development trajectory. (f) DCA embedding plot was derived from the “GATA_C” gene set and is colored by pseudotime. (g) Heatmap showing gene expression of cells and “GATA_C” target genes for the erythrocyte trajectory in columns and rows, respectively. (h) Barplot displays relevance scores for the five most and least relevant transcription factors in the monocyte development trajectory. (i) DCA embedding plot was derived from the “PU1_Q6” gene set and is colored by pseudotime. (j) Heatmap shows scaled gene expression of cells and “PU1_Q6” target genes for the monocyte trajectory in columns and rows, respectively. For both heatmaps, columns are ordered by pseudotime. Bars on top of heatmap indicate pseudotime, DCA coordinates one and two. Red and blue colors reflect high and low expression values.

**Figure 3. DrivAER identifies drivers underlying subtle transcriptional changes.** (a) Two groups of single cells were simulated and gene sets were created by sampling a mixture of truly differentially expressed (DE) genes and random genes. (b) The global embedding using all genes is visualized using UMAP. (c) The DCA embedding for a gene set consisting of all truly

DE genes is depicted. For both (b) and (c), cells are colored by group. (d) Relevance scores (y-axis) for gene sets ranging in the fraction of truly DE genes (x-axis) are displayed across implementations of DrivAER differing in the underlying dimension reduction methods. (e) Relevance scores (y-axis) for gene sets ranging in the fraction of truly DE genes (x-axis) are displayed using random forest (red) and support vector machine (blue) classification models. (f) Relevance scores (y-axis) for gene sets ranging in the fraction of truly DE genes (x-axis) are displayed across various configurations of the hidden layer. (g) Boxplot shows significantly different relevance scores between ten bootstrap runs of completely random gene sets (red) and gene sets consisting of 20% truly DE genes (blue) (One-sided t-test, P = 0.0467). The boxes represent the interquartile range, the horizontal line in the box is the median, and the whiskers represent 1.5 times the interquartile range. (h) PAGODA's adjusted z-scores (y-axis) are displayed for gene sets ranging in the fraction of truly DE genes (x-axis). (i) VISION's autocorrelation statistic is displayed for gene sets ranging in the fraction of truly DE genes (x-axis). (j) DrivAER (default parameters) relevance scores (y-axis) are displayed for gene sets ranging in the fraction of truly DE genes (x-axis). The horizontal dashed line indicates 0.5, the accuracy of random guesses for a binary outcome. For (d), (e), (f), (h), (i) and (j) lines represent the smoothed values and gray colors represent the 95% confidence interval derived from the smoothing fit.

Figure 1

Click here to access/download;Figure;Figure1.pdf

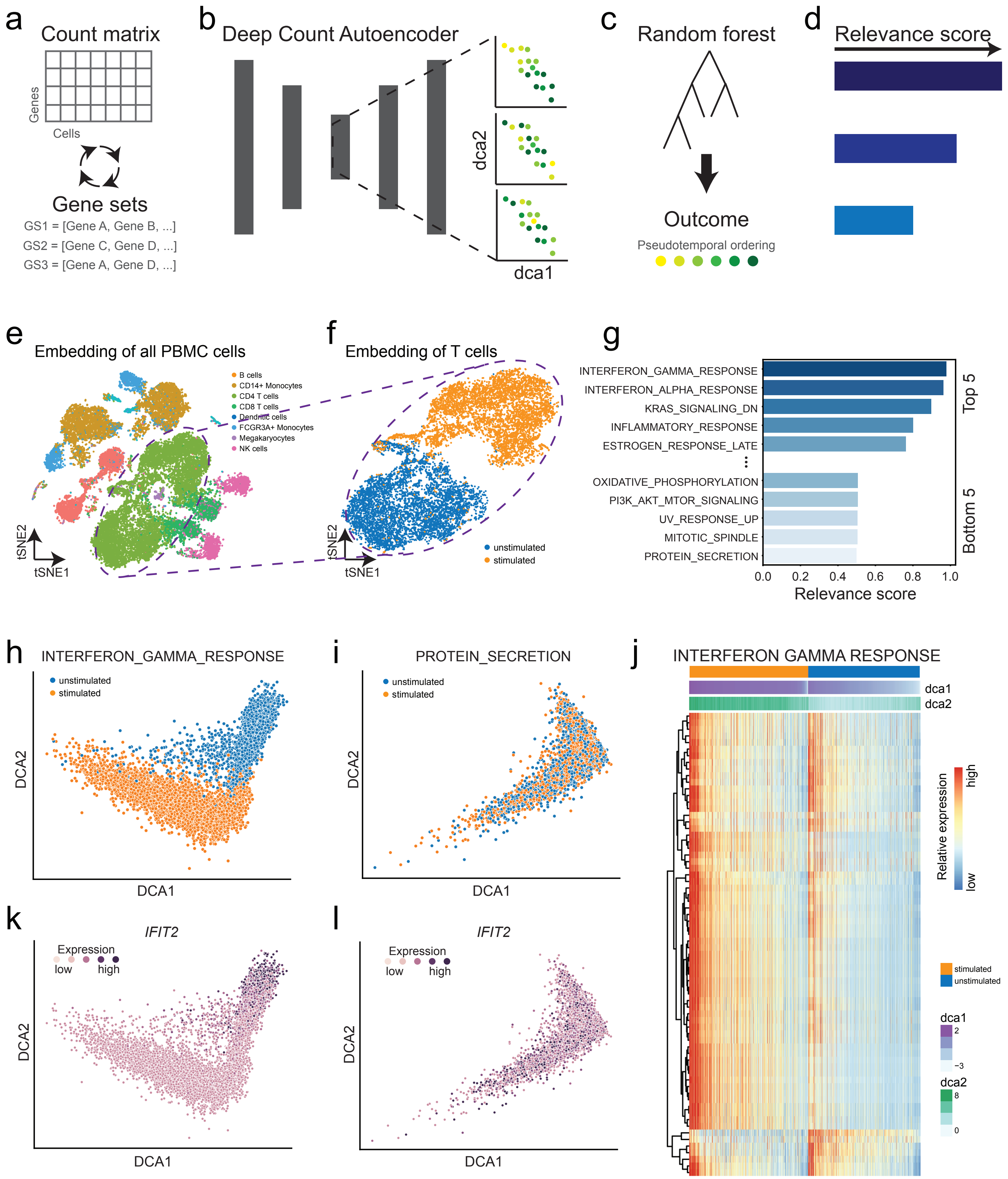

Figure 2 Click here to access/download;Figure;Figure2.pdf

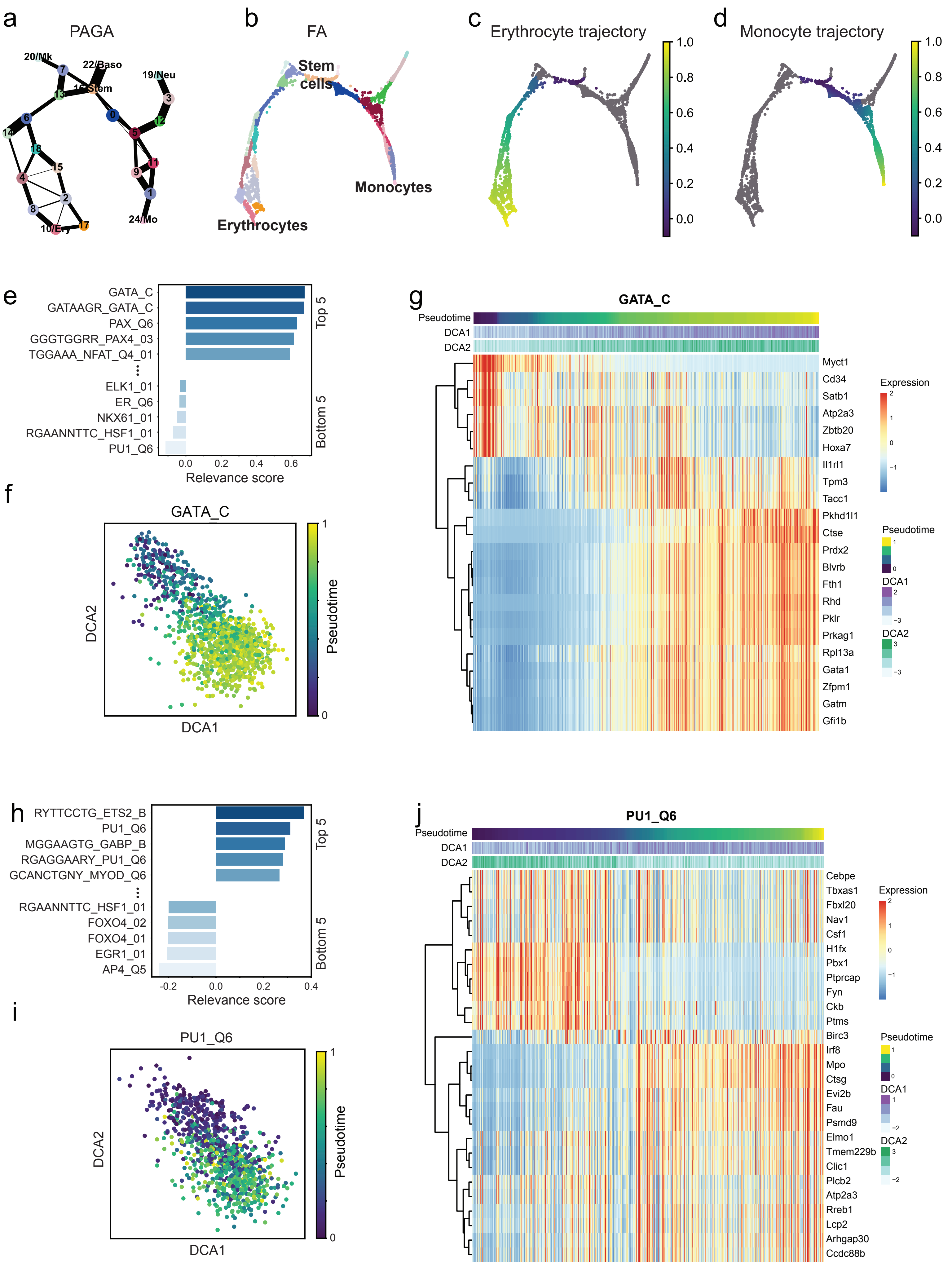

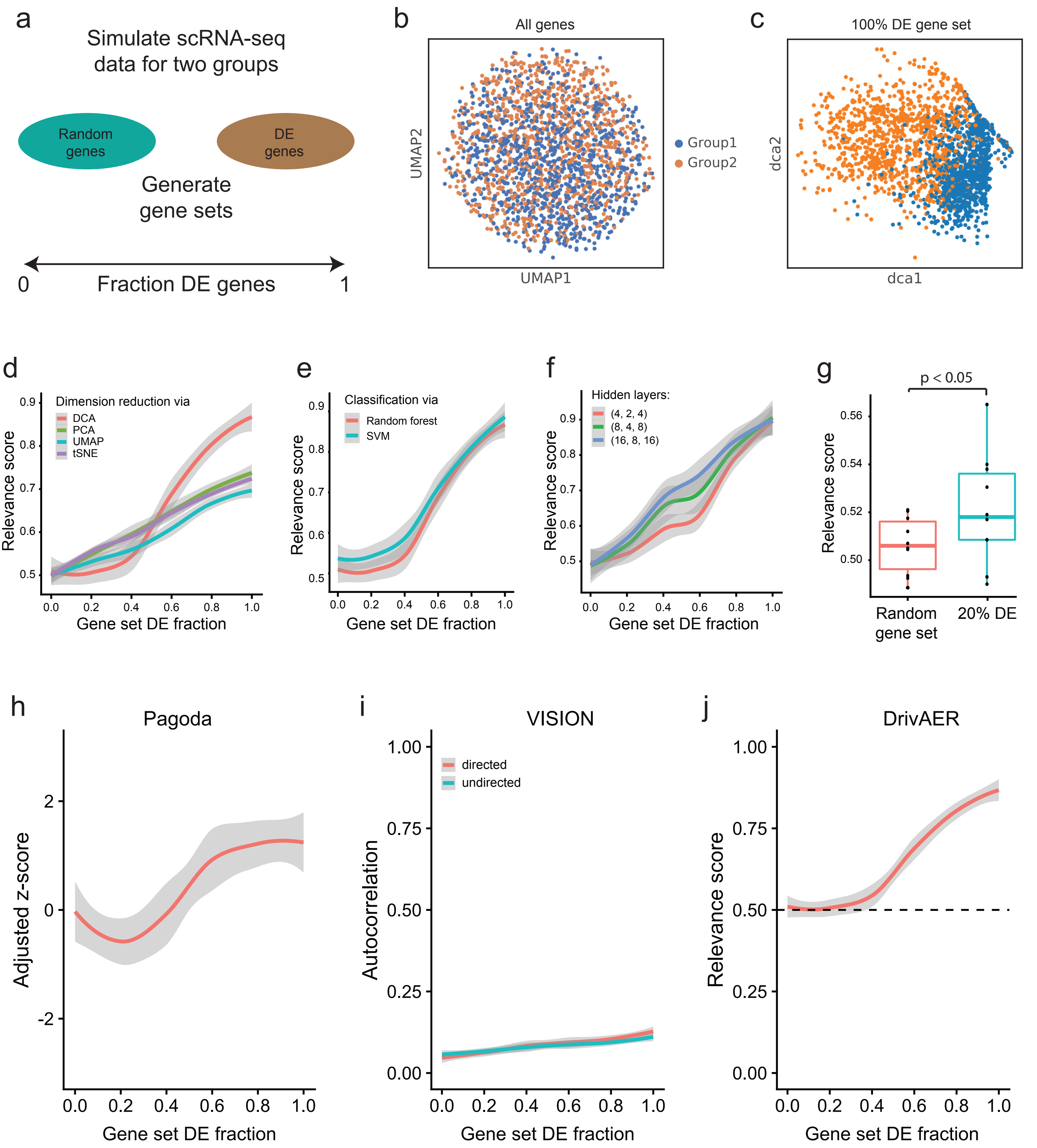
a
Simulate scRNA-seq data for two groups
Random genes
DE genes
Generate gene sets
0 Fraction DE genes 1
b
All genes
UMAP1
UMAP2
Group1
Group2
c
100% DE gene set
dca1
dca2
d
Dimension reduction via
DCA
PCA
UMAP
tSNE
Relevance score
Gene set DE fraction
e
Classification via
Random forest
SVM
Relevance score
Gene set DE fraction
f
Hidden layers:
(4, 2, 4)
(8, 4, 8)
(16, 8, 16)
Relevance score
Gene set DE fraction
g
p < 0.05
Relevance score
Random gene set
20% DE
h
Pagoda
Adjusted z-score
Gene set DE fraction
i
VISION
directed
undirected
Autocorrelation
Gene set DE fraction
j
DrivAER
Relevance score
Gene set DE fraction

Response to reviewer comments

Click here to access/download
**Supplementary Material**
Response to reviewers - 2.docx

Click here to access/download
**Supplementary Material**
Supplemental_material_revision_2.pdf

 Click here to access/download;Personal Cover;Editors requests.pdf

Dear Hans,

We are delighted to hear your decision on accepting our manuscript for publication at Gigascience! We have completed your editorial requests (please find our point-by-point response below).

Before we proceed to the production stage, I have a few editorial requests:

1) Our data curators will contact you shortly to prepare an archival snapshot of your code and test data for our repository GigaDB. Please include a citation to this upcoming GigaDB dataset (including the DOI link) to your reference list, and cite this in the data availability section.

Please follow this example format for the reference:

[xx] Author1 N, Author2 N, AuthorX N. Supporting data for "Title of your manuscript". GigaScience Database. 2020. http://dx.doi.orgxxxxxxxxxxxx

You will get the exact citation details from our curators.

In the data availability section, please write something along the lines, "An archival copy of the code and supporting data is available via the GigaScience repository, GigaDB [xx]".

*After interaction with your data curator Chris Armit, we have added the sentence including citation to the Data availability section.*

2) Please make sure the manuscript is formatted according to our guidelines (in particular, references should be numbered in the order of appearance, not in alphabetical order).

*We have changed the reference format.*

3) Thanks for adding the bio.tools identifier, please also register the tool with SciCrunch.org to receive an RRID (Research Resource Identification Initiative ID), and include this in the "code availability" section in your manuscript.

*We have registered DrivAER at SciCrunch.org and added the ID to the Code Availability section.*

4) At this stage, you can remove all highlighting/tracking of changes in colour that was made for the purpose of peer review.

*We have removed all highlighting/tracking of changes in colour.*

5) On the title page, please add ORCID IDs for all authors, if available.

*We have added the ORCID IDs for all authors to the title page.*

Once you have made the additions, please submit a revised manuscript online at:

https://www.editorialmanager.com/giga/

If you have forgotten your username or password please use the "Send Login Details" link to get your login information. For security reasons, your password will be reset.

Please also ensure that your revised manuscript conforms to the journal style, which can be found in the Instructions for Authors on the journal homepage. If the data and code has been modified in the revision process please be sure to update the public versions of this too.
