# Supplementary material for: DrivAER: Identification of driving transcriptional programs in single-cell RNA sequencing data
Source: Gigascience. 2020 Dec 10;9(12):giaa122. doi: 10.1093/gigascience/giaa122 (PMC7727875; doi:10.1093/gigascience/giaa122)
Supplement: giaa122_Supplemental_File [file giaa122_supplemental_file.pdf]

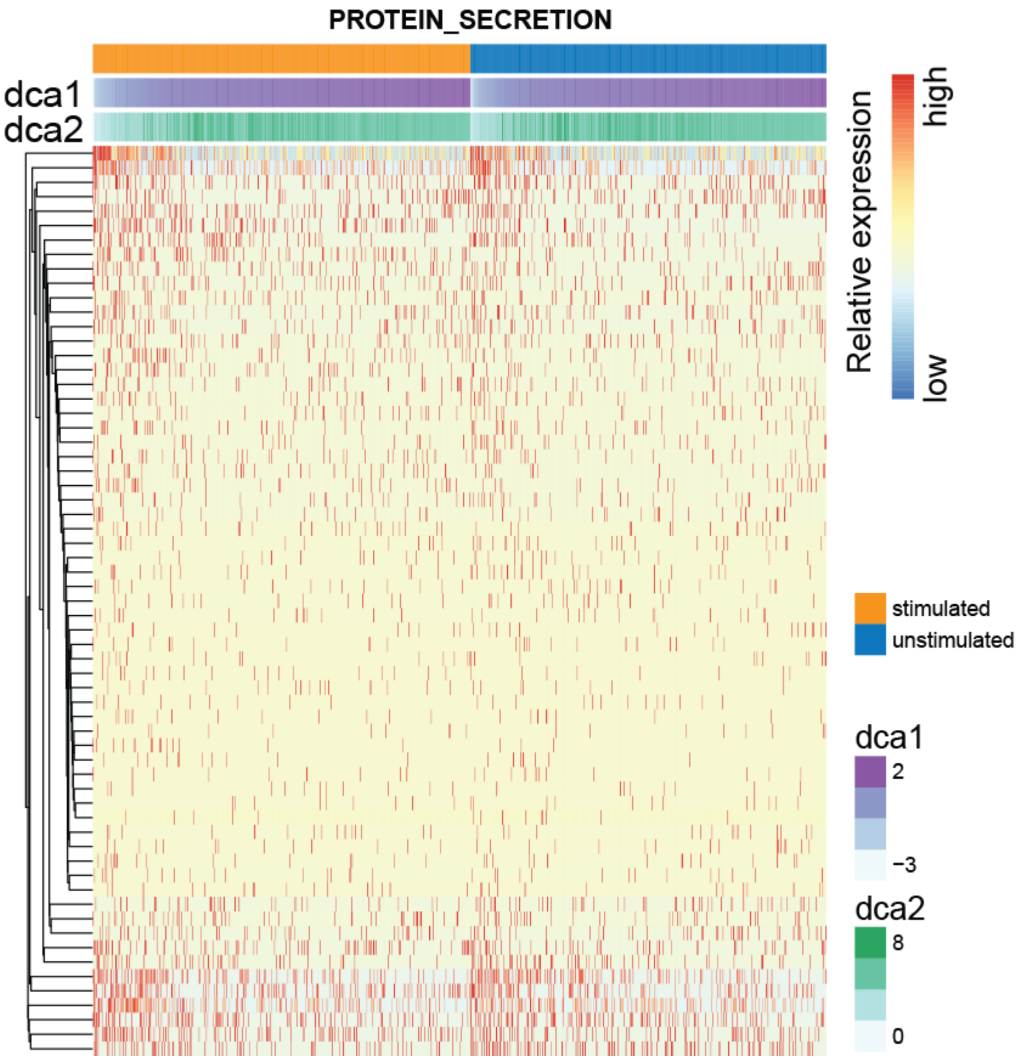

Figure S1. Heatmap shows gene expression of PROTEIN_SECRETION gene set and cells in rows and columns, respectively. Columns are ordered first by stimulation status and second by DCA coordinates. Bars on top of heatmap represent stimulation status and DCA coordinates one and two. Red and blue colors correspond to high and low relative expression values.

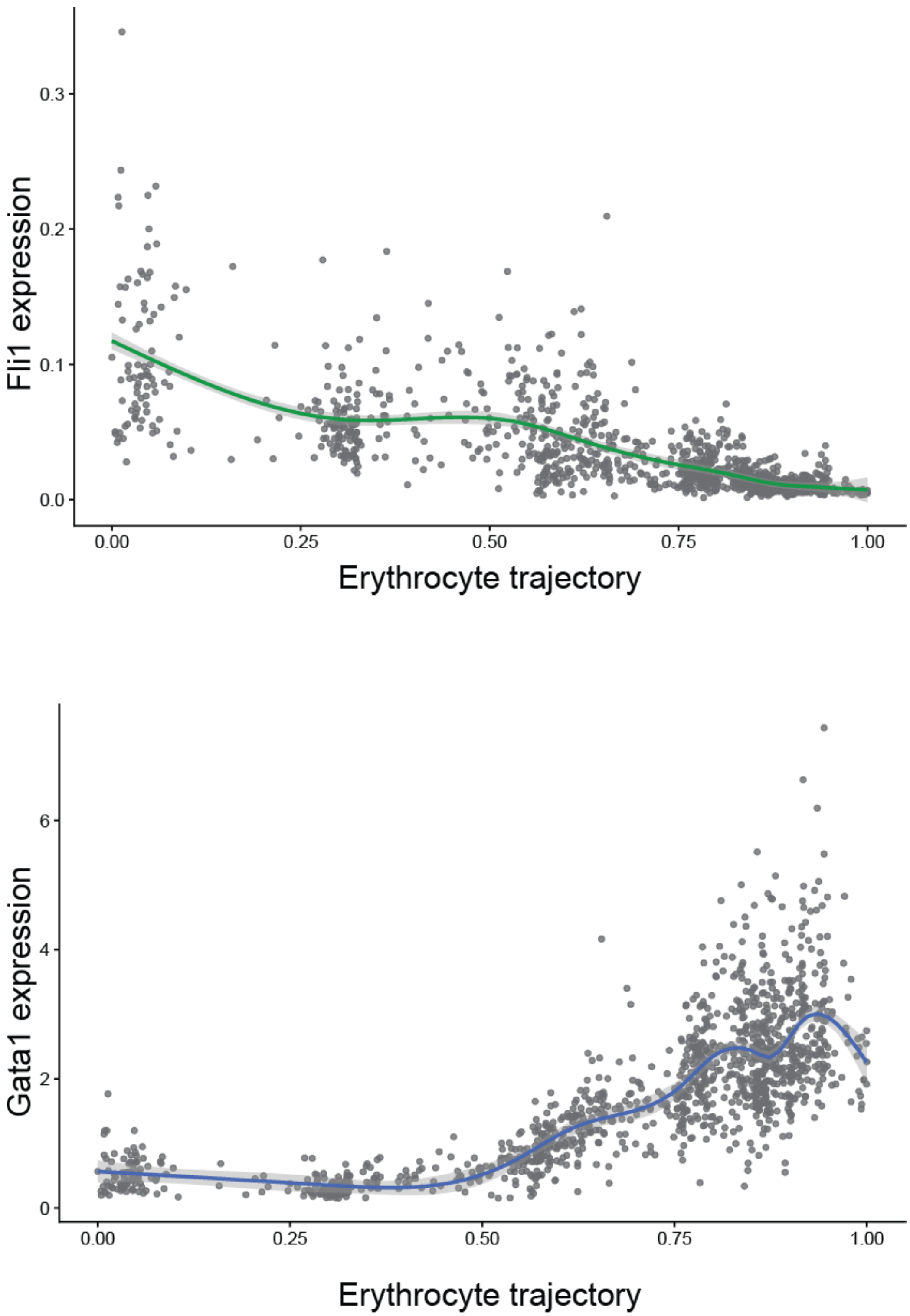

Figure S2. Plots show *Fli1* (top) and *Gata1* (bottom) expression along the Erythrocyte trajectory. Grey points indicate cells. The green and blue lines represent smoothed expression estimates.

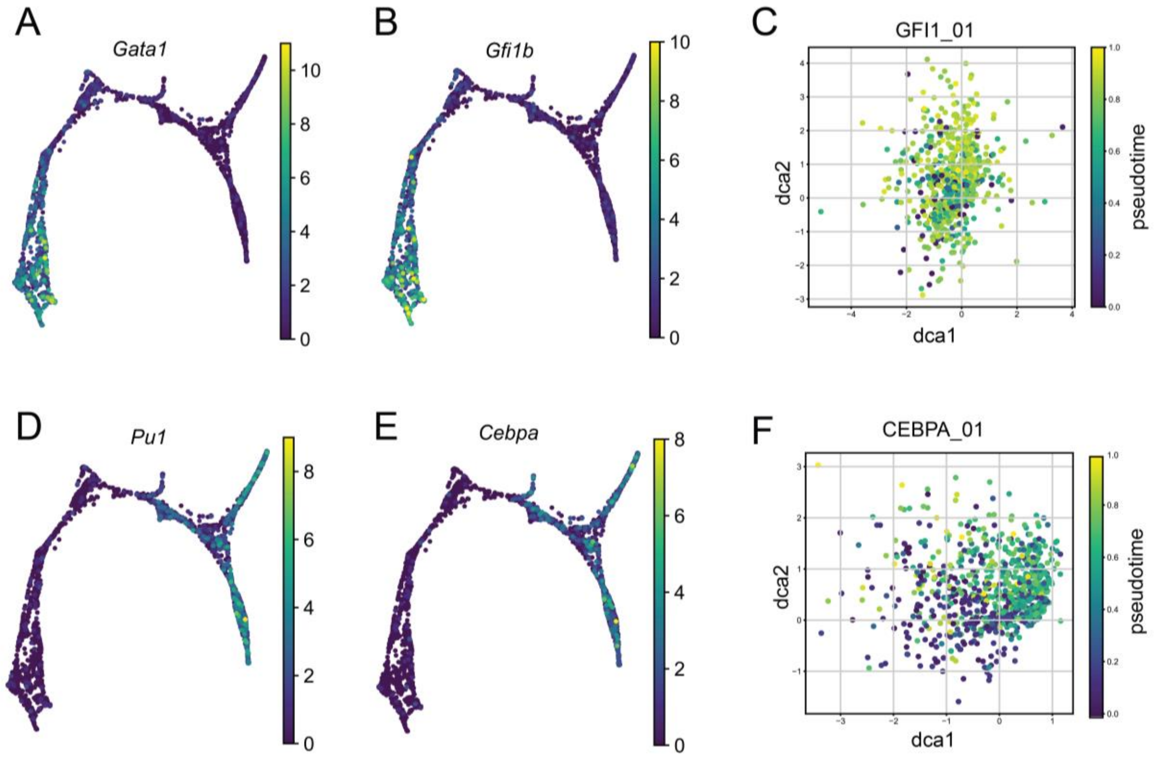

Figure S3. Expression of *Gata1* (A) and *Gfi1b* (B) along the erythrocyte trajectory shows similar pattern. However, DCA embedding derived from "GFI1_01" gene set shows poor association with pseudotime (C). Expression of *Pu1* (D) and *Cebpa* (E) along the monocyte trajectory shows similar pattern. However, DCA embedding derived from "CEBPA_01" gene set shows poor association with pseudotime (F).

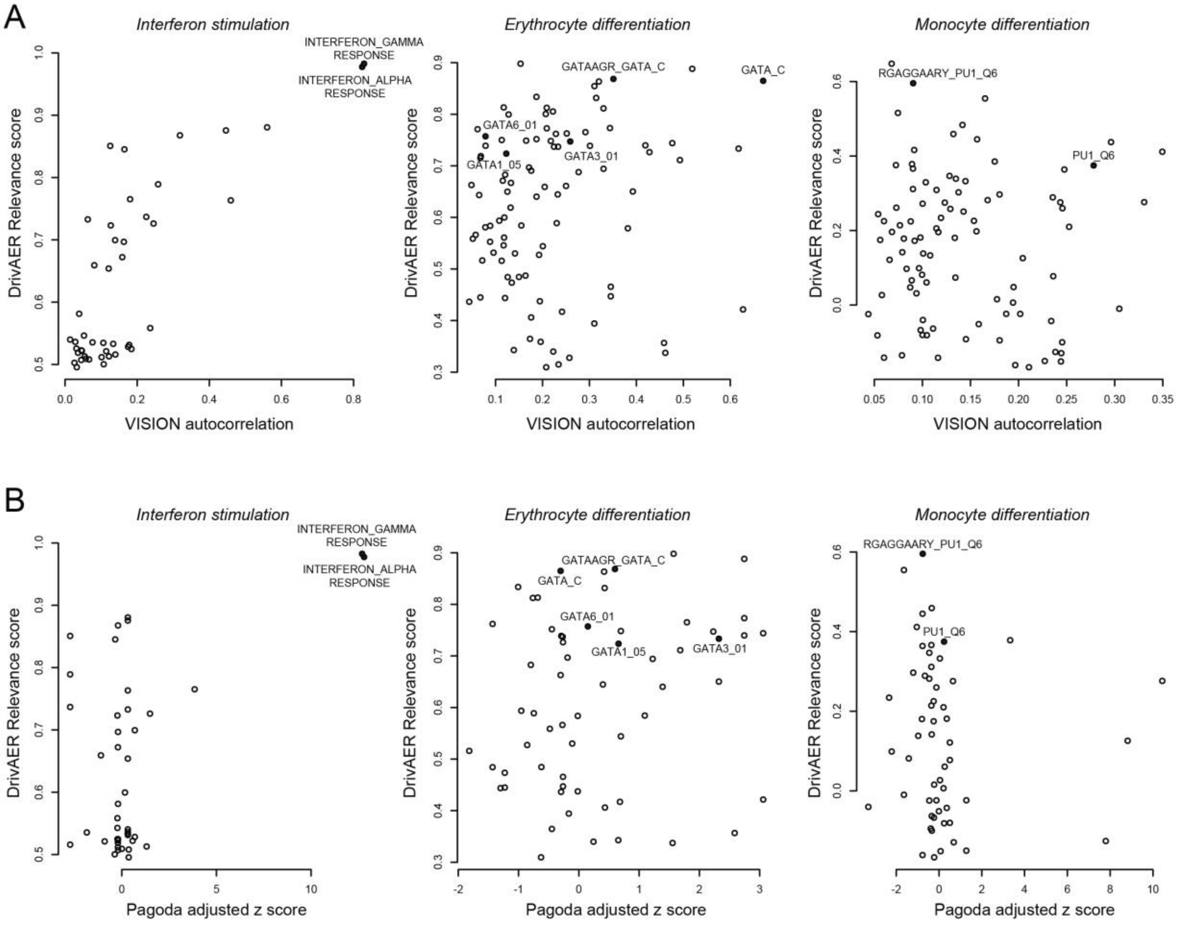

Figure S4. (A) Scatter plots depict VISION autocorrelation statistic (x-axis) and DrivAER relevance scores (y-axis) for the Interferon stimulation (left), erythrocyte (middle) and monocyte (right) trajectories. (B) Scatter plots depict PAGODA adjusted z-scores (x-axis) and DrivAER relevance scores (y-axis) for the Interferon stimulation (left), erythrocyte (middle) and monocyte (right) trajectories. For all panels, points represent gene sets and exemplary gene sets are highlighted.

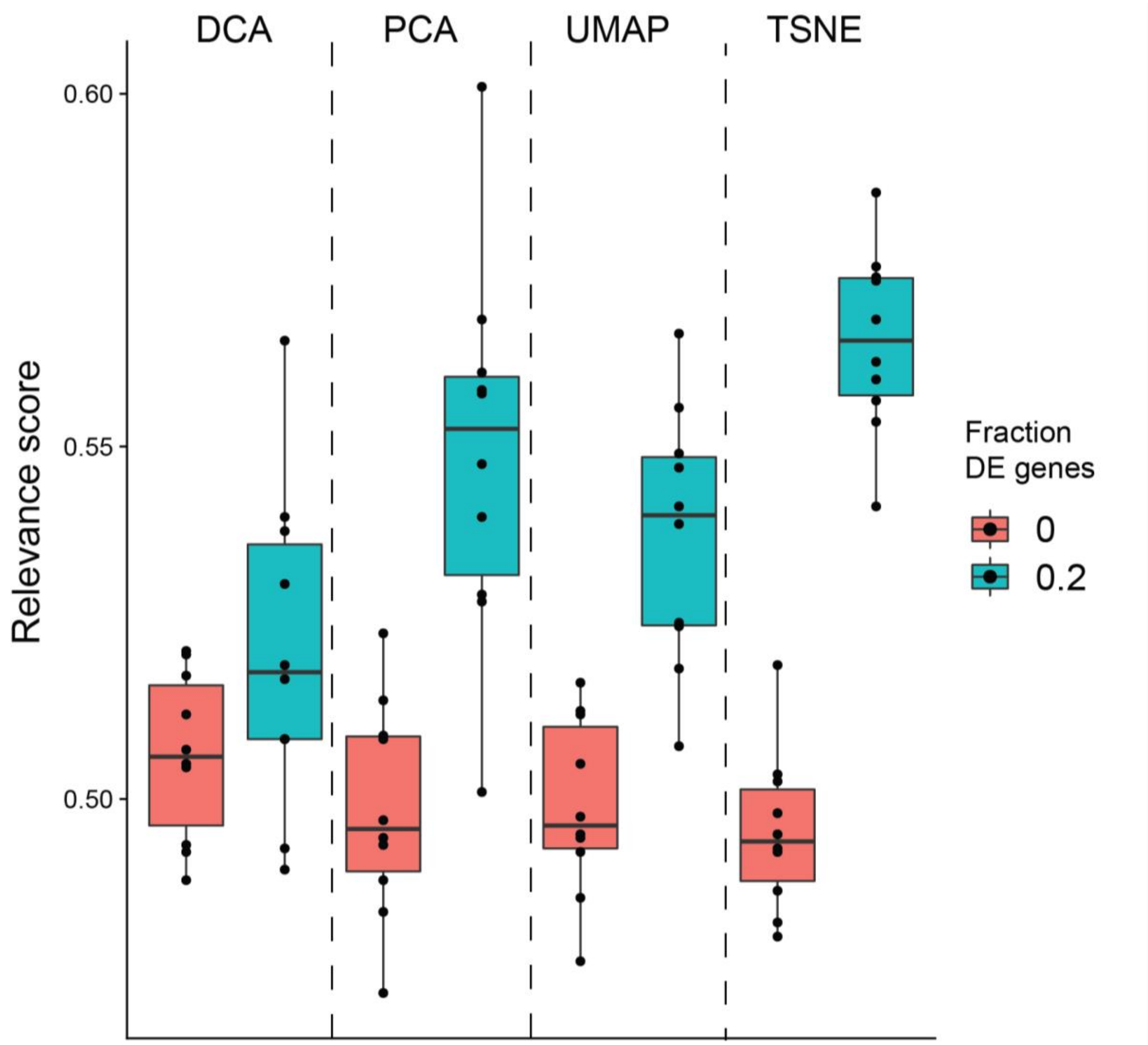

Figure S5. Boxplot shows relevance scores between completely random gene sets (red) and gene sets containing 20% truly DE genes (blue) differing in the underlying dimension reduction methods. From left to right, dimension reduction was based on DCA, PCA, UMAP and tSNE.

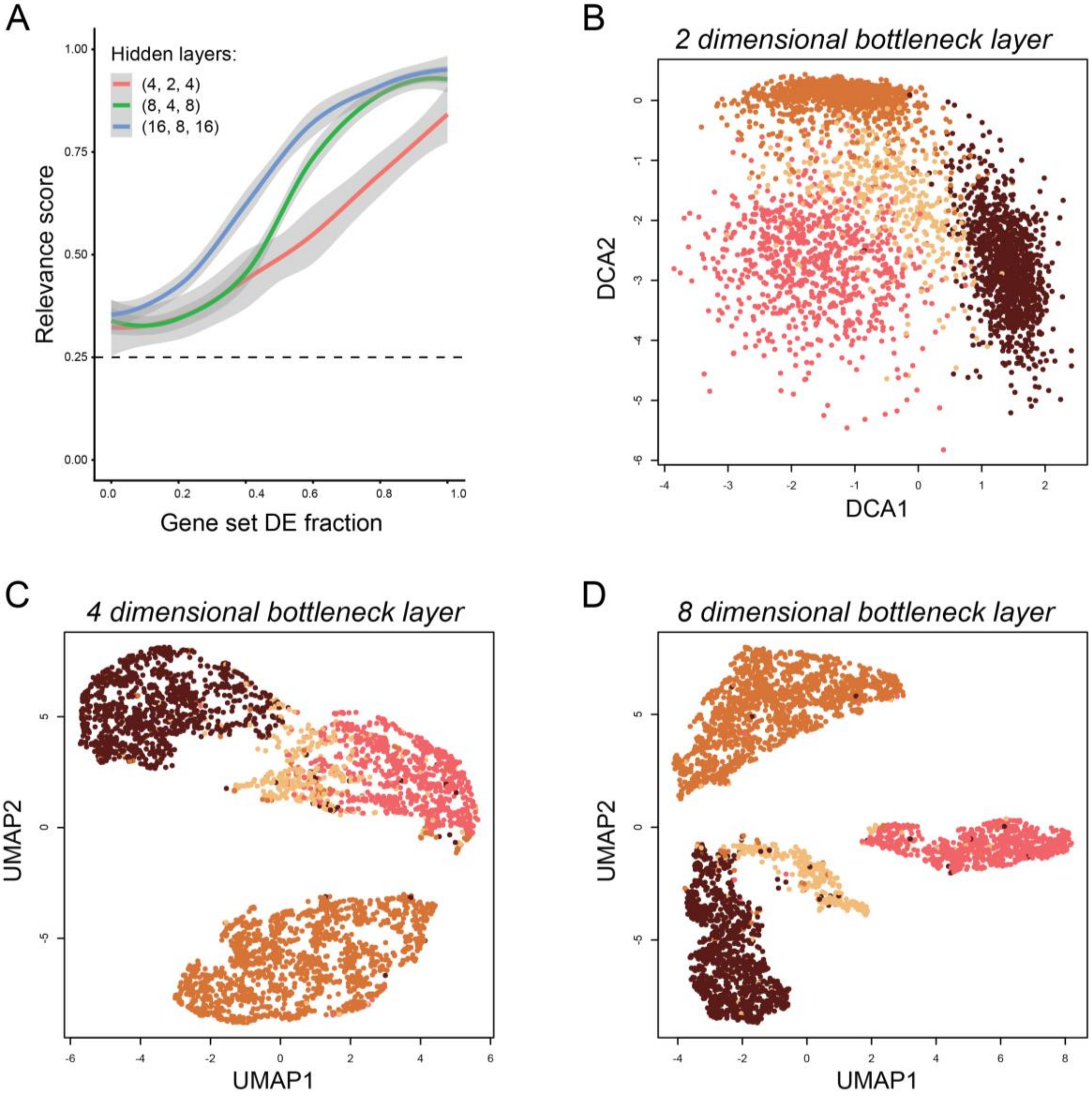

Figure S6. Complex four group simulation analysis. (A) Relevance scores (y-axis) for gene sets ranging in the fraction of truly DE genes (x-axis) are displayed across various configurations of the hidden layer. The horizontal dashed line indicates 0.25, the accuracy of random guesses for an outcome with four categories. (B) The DCA embedding derived from a two dimensional bottleneck layer for a gene set consisting of all truly DE genes is depicted. Embedding derived from four (C) and eight (D) dimensional bottleneck layers are visualized in two dimensions using UMAP. For B), C) and D), cells are colored by group.
