# Supplementary material for: DrivAER: Identification of driving transcriptional programs in single-cell RNA sequencing data
Source: Gigascience. 2020 Dec 10;9(12):giaa122. doi: 10.1093/gigascience/giaa122 (PMC7727875; doi:10.1093/gigascience/giaa122)
Supplement: giaa122_Response_to_Reviewer_Comments_Original_Submission [file giaa122_response_to_reviewer_comments_original_submission.pdf]

Author's Response To Reviewer Comments

Close

Response to Reviewers:
Manuscript ID: GIGA-D-20-00038
Title: DrivAER: Identification of driving transcriptional programs in single-cell RNA sequencing data

We thank the four reviewers for their valuable comments on our manuscript. In the revision, we have addressed each comment and revised the manuscript accordingly. Our point-by-point responses to reviewers are provided in the Supplementary Material.

Close
