# Supplementary material for: DrivAER: Identification of driving transcriptional programs in single-cell RNA sequencing data
Source: Gigascience. 2020 Dec 10;9(12):giaa122. doi: 10.1093/gigascience/giaa122 (PMC7727875; doi:10.1093/gigascience/giaa122)
Supplement: giaa122_Response_to_Reviewer_Comments_Revision_2 [file giaa122_response_to_reviewer_comments_revision_2.pdf]

Author's Response To Reviewer Comments

Close

We would like to thank the editor and reviewers for their input.

Close
