# Supplementary material for: DrivAER: Identification of driving transcriptional programs in single-cell RNA sequencing data
Source: Gigascience. 2020 Dec 10;9(12):giaa122. doi: 10.1093/gigascience/giaa122 (PMC7727875; doi:10.1093/gigascience/giaa122)
Supplement: giaa122_Reviewer_1_Report_Original_Submission — Christoph Ziegenhain -- 2/23/2020 Reviewed [file giaa122_reviewer_1_report_original_submission.pdf]

Reviewer Report

Title: DrivAER: Identification of driving transcriptional programs in single-cell RNA sequencing data

Version: Original Submission **Date:** 2/23/2020

Reviewer name: Christoph Ziegenhain

Reviewer Comments to Author:

In this manuscript, Simon and colleagues describe a novel algorithm to link scRNA-seq data to gene expression signatures.
While the concept seems like a timely contribution to help relate biological signals to concrete transcriptional programs of interest from the ever-increasing amounts of sequenced cells, the manuscript in the present form lacks rigor and thoroughness.
Major points:
- In both major application examples of interferon stimulation and hematopoietic lineage specification, the authors extensively use the known ground truth to guide their analysis. In the example of interferon stimulation, the authors preselect the MolSigDB annotations that play a role in this specific perturbation. In practice however, many researchers will not already know the system at hand well. How does DriveAER perform when removing this circular logic and for example all MolSigDB pathways are used as input into this dataset? In the same vein, Figure 1j would be interesting to see when not sorted according to the known perturbation but purely on the observed DCA scoring?
The same argument holds true for the application to the erythrocyte vs monocytes distinction, where specifically relevant TF modules were preselected.
- Both biological systems used as an example represent rather huge biological signals. How does DriveAER fare when more nuanced transcriptional changes are present in the data at hand?
- The comparison to VISION is a good idea, but the execution is haphazard. For the general reader, a minimal description of the VISION algorithm is necessary. Also the lack of correlation in outcomes (apart from the GATA and PU1 key TFs) in the blood development examples needs to be investigated further.
- As the authors state themselves in the discussion, the use of the deep count auto-encoder for dimensionality reduction is not a requirement for applying DrivAERs random forest classification. I would like to see a comparison of performance with other common dimensionality reduction methods like tSNE and UMAP. It seems that for example, a transcriptional response as strong as interferon stimulation would surely separate populations clearly in either method.
Minor points:
- On p. 6, the authors write: "Secondly, due to the low RNA capture rate in scRNA-seq, generally lowly expressed TFs are not detected reliably". This statement is simply not true in light of the vast differences in sensitivity and typical sequencing depth of various scRNA-seq methods and the authors should discuss this correctly.
- In a technical manuscript, the method section should contain more details and precision throughout. A few examples: a) How were PBMCs clustered to extract the T cells from the interferon

stimulation dataset? b) "lowly expressed genes and cells with less than 3 counts were filtered out". Surely this cutoff refers to genes with less than x counts over all cells and the cell filtering cutoff must have been different? c) In several places the authors refer to vignettes or tutorials for tools like Scanpy and VISION. Since those tools and their documentation will typically be developed further the exact versions and settings for all relevant steps should be listed as performed in this specific work.

- In the last paragraph on p. 4, a sentence partially duplicated:

"described a transcriptional response to interferon stimulation"

- Figure legend 1c appears to be missing.
- At the end of the introduction on p. 4 the authors say that they applied their tool to two datasets vs in the next paragraph they mentioned that they actually looked at three datasets.

Level of Interest

Please indicate how interesting you found the manuscript: Choose an item.

Quality of Written English

Please indicate the quality of language in the manuscript: Choose an item.

Declaration of Competing Interests

Please complete a declaration of competing interests, considering the following questions:

- Have you in the past five years received reimbursements, fees, funding, or salary from an organisation that may in any way gain or lose financially from the publication of this manuscript, either now or in the future?
- Do you hold any stocks or shares in an organisation that may in any way gain or lose financially from the publication of this manuscript, either now or in the future?
- Do you hold or are you currently applying for any patents relating to the content of the manuscript?
- Have you received reimbursements, fees, funding, or salary from an organization that holds or has applied for patents relating to the content of the manuscript?
- Do you have any other financial competing interests?
- Do you have any non-financial competing interests in relation to this paper?

If you can answer no to all of the above, write 'I declare that I have no competing interests' below. If your reply is yes to any, please give details below.

I declare that I have no competing interests

I agree to the open peer review policy of the journal. I understand that my name will be included on my report to the authors and, if the manuscript is accepted for publication, my named report including any attachments I upload will be posted on the website along with the authors' responses. I agree for my report to be made available under an Open Access Creative Commons CC-BY license (http://creativecommons.org/licenses/by/4.0/). I understand that any comments which I do not wish to be included in my named report can be included as confidential comments to the editors, which will not be published.

Choose an item.

To further support our reviewers, we have joined with Publons, where you can gain additional credit to further highlight your hard work (see: https://publons.com/journal/530/gigascience). On publication of this paper, your review will be automatically added to Publons, you can then choose whether or not to claim your Publons credit. I understand this statement.

Yes Choose an item.
