# Supplementary material for: DrivAER: Identification of driving transcriptional programs in single-cell RNA sequencing data
Source: Gigascience. 2020 Dec 10;9(12):giaa122. doi: 10.1093/gigascience/giaa122 (PMC7727875; doi:10.1093/gigascience/giaa122)
Supplement: giaa122_Reviewer_2_Report_Original_Submission — Casey S. Greene -- 2/25/2020 Reviewed [file giaa122_reviewer_2_report_original_submission.pdf]

Reviewer Report

Title: DrivAER: Identification of driving transcriptional programs in single-cell RNA sequencing data

Version: Original Submission **Date:** 2/25/2020

Reviewer name: Casey S. Greene

Reviewer Comments to Author:

The authors describe DrivAER, an auto-encoder method for identifying transcriptional programs in single-cell RNA-seq data. The approach uses the Deep Count Autoencoder, so the primary difference here is restricting the training set to genes from a specific curated gene set. The representation learned from that set is then evaluated for concordance with external information to evaluate the extent to which the set is relevant. A major strength of this work is that it is described as plugging into well-established and existing software for single-cell analysis (scanpy) which is much appreciated.
There are some oddities around the representation being learned from only genes in a curated pathway that may pose significant limitations for the use of the method in practice. It was not what I expected from the use of autoencoders and the abstract, so fewer of the problems that our group thinks about would map well to this framework. Often when we want to use an unsupervised learning approach, our goal is to identify potential signatures that may be important but that may not already be captured in databases. We and others have looked into this in some detail over a few years with auto-encoder approaches for bulk RNA profiling (https://www.ncbi.nlm.nih.gov/pubmed/25592575, https://www.ncbi.nlm.nih.gov/pubmed/26818848, https://www.ncbi.nlm.nih.gov/pubmed/27822512, https://www.ncbi.nlm.nih.gov/pubmed/28711280, https://www.biorxiv.org/content/10.1101/278739v2). Recently, a penalized latent variable approach (PLIER, https://www.ncbi.nlm.nih.gov/pubmed/31249421) presented a method to learn a low-dimensional representation that is somewhat guided by prior knowledge, but it can also learn latent variables that are not associated with pathways. It may be helpful to discuss these limitations or considerations in the context of a method that uses such a low-dimensional auto-encoder.
Performance comparisons, particularly in this domain, can be fraught with parameter selection-driven differences in performance (https://www.ncbi.nlm.nih.gov/pubmed/30963075). It's not clear that parameter tuning here is equal between both compared methods, so it may be the case that differences are driven by the Continental Breakfast Included Effect described in the linked paper. It would be helpful to describe the extent to which steps were taken to mitigate this risk.
Since this uses curated pathways, it would help to examine even simpler gene set based methods. I imagine that a comparison with something like a comparison with regressing against the average expression of genes in the curated pathway would be worth at least testing as an ultra-simple baseline.
One thing the authors might consider would be to try to predict _all other_ genes using just the pathway genes as input. Genes that are predictable from the pathway genes alone might suggest new players in (or downstream of) the pathway. Of course, it might also totally break the ability of the auto-encoder to train, but I think there is some non-zero chance this works. I can imagine a number of settings where

we'd find this type of approach valuable in our own work.
Minor:
* The repository is MIT licensed but the specific license is not mentioned in the paper itself. It could be helpful to provide this information.
* There are ipynb checkpoints in the repo. Is this intended?

Level of Interest

Please indicate how interesting you found the manuscript: Choose an item.

Quality of Written English

Please indicate the quality of language in the manuscript: Choose an item.

Declaration of Competing Interests

Please complete a declaration of competing interests, considering the following questions:

- Have you in the past five years received reimbursements, fees, funding, or salary from an organisation that may in any way gain or lose financially from the publication of this manuscript, either now or in the future?
- Do you hold any stocks or shares in an organisation that may in any way gain or lose financially from the publication of this manuscript, either now or in the future?
- Do you hold or are you currently applying for any patents relating to the content of the manuscript?
- Have you received reimbursements, fees, funding, or salary from an organization that holds or has applied for patents relating to the content of the manuscript?
- Do you have any other financial competing interests?
- Do you have any non-financial competing interests in relation to this paper?

If you can answer no to all of the above, write 'I declare that I have no competing interests' below. If your reply is yes to any, please give details below.

I declare that I have no competing interests.

I agree to the open peer review policy of the journal. I understand that my name will be included on my report to the authors and, if the manuscript is accepted for publication, my named report including any attachments I upload will be posted on the website along with the authors' responses. I agree for my report to be made available under an Open Access Creative Commons CC-BY license (http://creativecommons.org/licenses/by/4.0/). I understand that any comments which I do not wish to be included in my named report can be included as confidential comments to the editors, which will not be published.

Choose an item.

To further support our reviewers, we have joined with Publons, where you can gain additional credit to further highlight your hard work (see: https://publons.com/journal/530/gigascience). On publication of this paper, your review will be automatically added to Publons, you can then choose whether or not to claim your Publons credit. I understand this statement.

Yes Choose an item.
