# Supplementary material for: DrivAER: Identification of driving transcriptional programs in single-cell RNA sequencing data
Source: Gigascience. 2020 Dec 10;9(12):giaa122. doi: 10.1093/gigascience/giaa122 (PMC7727875; doi:10.1093/gigascience/giaa122)
Supplement: giaa122_Reviewer_3_Report_Original_Submission — Vladimir V. Galatenko -- 2/28/2020 Reviewed [file giaa122_reviewer_3_report_original_submission.pdf]

Reviewer Report

Title: DrivAER: Identification of driving transcriptional programs in single-cell RNA sequencing data

Version: Original Submission **Date:** 2/28/2020

Reviewer name: Vladimir V. Galatenko

Reviewer Comments to Author:

In their manuscript L.M. Simon, F. Yan and Z. Zhao described a method for an analysis of single-cell RNA sequencing data aimed at identification of gene sets from a predefined set collection that are most relevant for a specified outcome variable. Remarkably, the method works at the gene set level, without estimating differential expression characteristics for individual genes.

Formally the method can be describes as follows. Each gene set from a predefined collection is considered individually, relevance of this set to an outcome variable is quantified, and top-scoring sets are claimed to be the most relevant ones. For an individual set, expression profiles for every cell are limited to genes from this set, and dimension reduction is performed (specifically, Deep Count Autoencoder (DCA) method is used, with a dimensionality of an embedding space equal to two). Then a random forest model is applied to test whether resulting 2D coordinates can be used to reliably predict values of a user-specified outcome variable. Random forest accuracy is directly taken as a relevance score.

As a proof of concept, the authors described results obtained by applying their method to several public datasets, covering cases of both categorical and continuous outcome variables.

The manuscript is well structured and well written, it is potentially interesting to a broad readership. Yet, minor editing is still required. For example, a phrase "described a transcriptional response…" is duplicated in the second paragraph of Results section; in the last paragraph of Background section the authors stated that the proposed method had been evaluated by application to two datasets, but then in the second paragraph of Results specified that three datasets had been reanalyzed.

More essential comments and remarks are associated with the method justification, both in relation to method design decisions and to comparison with competing methods. Regarding method design decisions, it is recommended to justify (e.g., by applying to simulated and/or real data) that

- within the proposed method scheme DCA is better or at least is not inferior to other dimension reduction algorithms (e.g., widely used t-SNE and UMAP) - currently it is mentioned in Discussion and conclusions section as a direction for future work,
- dimensionality of embedding space set to two is an optimal selection,
- random forest is a good choice in comparison with other approaches (e.g., with support vector machines / support vector regression).

Regarding a comparison with competing methods, it is recommended to present results of comparison of the proposed method with a standard combination of a differential expression analysis and enrichment analysis (that would be a kind of a comparison with a "base line"). Furthermore, in the comparison with VISION it is desirable to have a more detailed discussion of differences between the

methods and, in particular, between formal problem statements behind the methods (e.g., required input data such as an outcome variable).
In addition, it would desirable to have a discussion of a relevance score interpretability and recommendations on assessing "potential significance" for a given value of relevance score, as well as a discussion of gene sets identified for the reanalyzed datasets not limited to the top hit, but covering at least 3-5 gene sets with highest scores. It would be also interesting to find a short overview of potential sources for getting values of an outcome variable in the revised version of the manuscript (in particular, an answer to the question if there are any sources except single cell RNA sequencing data itself that can be practically used to obtain values for a meaningful outcome variable). Finally, it would be desirable to discuss potential applicability of the proposed method or method concept to a wider spectrum of datasets, not only to single cell RNA sequencing data.
Overall, from my viewpoint the concept of the proposed method is very interesting, but addressing the above comments and remarks is suggested before publication.

Level of Interest

Please indicate how interesting you found the manuscript: Choose an item.

Quality of Written English

Please indicate the quality of language in the manuscript: Choose an item.

Declaration of Competing Interests

Please complete a declaration of competing interests, considering the following questions:

- Have you in the past five years received reimbursements, fees, funding, or salary from an organisation that may in any way gain or lose financially from the publication of this manuscript, either now or in the future?
- Do you hold any stocks or shares in an organisation that may in any way gain or lose financially from the publication of this manuscript, either now or in the future?
- Do you hold or are you currently applying for any patents relating to the content of the manuscript?
- Have you received reimbursements, fees, funding, or salary from an organization that holds or has applied for patents relating to the content of the manuscript?
- Do you have any other financial competing interests?
- Do you have any non-financial competing interests in relation to this paper?

If you can answer no to all of the above, write 'I declare that I have no competing interests' below. If your reply is yes to any, please give details below.

I declare that I have no competing interests.

I agree to the open peer review policy of the journal. I understand that my name will be included on my report to the authors and, if the manuscript is accepted for publication, my named report including any attachments I upload will be posted on the website along with the authors' responses. I agree for my report to be made available under an Open Access Creative Commons CC-BY license (http://creativecommons.org/licenses/by/4.0/). I understand that any comments which I do not wish to be included in my named report can be included as confidential comments to the editors, which will not be published.

Choose an item.

To further support our reviewers, we have joined with Publons, where you can gain additional credit to further highlight your hard work (see: https://publons.com/journal/530/gigascience). On publication of this paper, your review will be automatically added to Publons, you can then choose whether or not to claim your Publons credit. I understand this statement.

Yes Choose an item.
