# Supplementary material for: DrivAER: Identification of driving transcriptional programs in single-cell RNA sequencing data
Source: Gigascience. 2020 Dec 10;9(12):giaa122. doi: 10.1093/gigascience/giaa122 (PMC7727875; doi:10.1093/gigascience/giaa122)
Supplement: giaa122_Reviewer_3_Report_Revision_1 — Vladimir V. Galatenko -- 6/8/2020 Reviewed [file giaa122_reviewer_3_report_revision_1.pdf]

Reviewer Report

Title: DrivAER: Identification of driving transcriptional programs in single-cell RNA sequencing data

Version: Revision 1 **Date:** 6/8/2020

Reviewer name: Vladimir V. Galatenko

Reviewer Comments to Author:

As noted in the review of the initial version of the manuscript, L.M. Simon, F. Yan and Z. Zhao described a novel method for an analysis of single-cell RNA sequencing data aimed at identification of gene sets from a predefined set collection that are most relevant for a specified outcome variable. Importantly, the described method operates at the gene set level and does not rely on estimations of differential expression characteristics for individual genes.

In the revised version, the authors added a description of a systematic validation study on simulated scRNA-seq data. This study addressed the majority of questions and critical comments from the review of the initial manuscript version. At the same time, it raised a number of new questions to the authors, probably because a discussion of the results of this validation study was minimalistic and in a certain sense the authors left interpretation of figures to a reader.

Major questions and comments

1) Does it follow from Fig. 3d that for the case of a relatively low fraction of DE genes in a gene set dimension reduction / 2D-embedding methods alternative to DCA significantly outperform DCA, and DCA starts to be an optimal choice only for the case of gene sets with more than 50% of DE genes? How a plot similar to Fig. 3g looks for t-SNE and PCA?

As 20% and, moreover, 40% of DE genes in a gene set actually seem to be a large fraction for practical cases, a fact that, e.g., t-SNE and even PCA outperform DCA at these fractions could be a solid argument for adding a support of these dimension reduction / 2D-embedding methods along with DCA in the software implementation.

2) Does it follow from Fig. 3f that even in the simple simulation settings an increase of the dimensionality (e.g., "compressing" data to 4D or 8D instead of 2D) significantly improves the performance for the case of 20% and, even more, for the case of 40%-60% of DE genes in a gene set? Does the same hold for other dimension reduction methods, e.g., t-SNE or PCA? How a plot similar to Fig. 3g looks for DCA with the (16,8,16) configuration of the hidden layer?

3) Along with VISION, the authors included Pagoda in their validation study. Yet, in spite of reviewers' recommendations, they did not compare their results vs. a conventional approach (i.e., analysis of differential expression of individual genes followed by a standard gene set enrichment analysis). It would be interesting to get an idea how significantly the proposed method outperforms this most conventional one.

4) It is also desirable to present (e.g., as a Supplementary) results for a similar validation study on simulated data with a less simplified data structure (e.g., three or four unbalanced groups). Theoretically, it could lead to different conclusions, for example, a benefit of a wider bottleneck layer

can turn out to be essentially higher.
Minor points.
1) P-value for Fig. 3g should be explicitly specified (and reporting of two-sided p-value, not one-sided one, is recommended).
2) A more detailed description of ideas of Pagoda and VISION is desirable (at least, in a Supplementary text). Currently it is almost impossible for a reader not familiar with original papers on these methods to understand approaches used by these methods and hence to grasp fundamental differences between approaches of VISION, Pagoda and DrivAER.
3) The red curve in Fig. 3f seems to be different from red curves shown in Figs. 3d,e,j. Does it mean that default configuration of the hidden layer for DCA was not (4,2,4)? In any case, this configuration should be explicitly specified in the text.
4) Similarly, more information should be provided about applying t-SNE and UMAP (at least, initialization type and main parameters - e.g., perplexity for t-SNE and number of nearest neighbors plus minimum distance for UMAP). Details for SVM application (e.g., kernel type, selection of hyper-parameters) should be explicitly specified as well.
5) For the red curve in Figs 3d,e,j values for DE fractions 0.0 and 0.2 look nearly identical, which seems to be inconsistent with Fig. 3g.
6) For all curves in Fig. 3d-j, if they were constructed based on values for fractions 0.0, 0.2, 0.4, 0.6, 0.8, 1.0, it makes sense to show these values for the X-axis instead of 0.25, 0.5 and 0.75, as it would simplify figure inspection and analysis.
Actually, the above questions and comments are driven primarily by an interest raised by the manuscript, and my overall recommendation is definitely to accept the manuscript for publication. At the same time, I hope that the authors would consider an option to update the text taking these question and comments into account, at least partially.

Level of Interest

Please indicate how interesting you found the manuscript: Choose an item.

Quality of Written English

Please indicate the quality of language in the manuscript: Choose an item.

Declaration of Competing Interests

Please complete a declaration of competing interests, considering the following questions:

- Have you in the past five years received reimbursements, fees, funding, or salary from an organisation that may in any way gain or lose financially from the publication of this manuscript, either now or in the future?

- Do you hold any stocks or shares in an organisation that may in any way gain or lose financially from the publication of this manuscript, either now or in the future?
- Do you hold or are you currently applying for any patents relating to the content of the manuscript?
- Have you received reimbursements, fees, funding, or salary from an organization that holds or has applied for patents relating to the content of the manuscript?
- Do you have any other financial competing interests?
- Do you have any non-financial competing interests in relation to this paper?

If you can answer no to all of the above, write 'I declare that I have no competing interests' below. If your reply is yes to any, please give details below.

I declare that I have no competing interests.

I agree to the open peer review policy of the journal. I understand that my name will be included on my report to the authors and, if the manuscript is accepted for publication, my named report including any attachments I upload will be posted on the website along with the authors' responses. I agree for my report to be made available under an Open Access Creative Commons CC-BY license (http://creativecommons.org/licenses/by/4.0/). I understand that any comments which I do not wish to be included in my named report can be included as confidential comments to the editors, which will not be published.

Choose an item.

To further support our reviewers, we have joined with Publons, where you can gain additional credit to further highlight your hard work (see: https://publons.com/journal/530/gigascience). On publication of this paper, your review will be automatically added to Publons, you can then choose whether or not to claim your Publons credit. I understand this statement.

Yes Choose an item.
