# Supplementary material for: DrivAER: Identification of driving transcriptional programs in single-cell RNA sequencing data
Source: Gigascience. 2020 Dec 10;9(12):giaa122. doi: 10.1093/gigascience/giaa122 (PMC7727875; doi:10.1093/gigascience/giaa122)
Supplement: giaa122_Reviewer_3_Report_Revision_2 — Vladimir V. Galatenko -- 9/21/2020 Reviewed [file giaa122_reviewer_3_report_revision_2.pdf]

Reviewer Report

Title: DrivAER: Identification of driving transcriptional programs in single-cell RNA sequencing data

Version: Revision 2 **Date:** 9/21/2020

Reviewer name: Vladimir V. Galatenko

Reviewer Comments to Author:

As noted in the review of the previous versions of the manuscript, L.M. Simon, F. Yan and Z. Zhao described a novel method (DrivAER) for an analysis of single-cell RNA sequencing data aimed at identification of gene sets from a predefined set collection that are most relevant for a specified outcome variable. Importantly, the described method operates at the gene set level and does not rely on estimations of differential expression characteristics for individual genes.
In the updated version of the manuscript, the authors took the reviewers' comments into account and changed the text and the plots accordingly. Some reviewers' questions remained unaddressed in the manuscript. For example, no results on the comparison of DrivAER vs. a conventional approach (i.e., analysis of differential expression of individual genes followed by a standard gene set enrichment analysis) have been presented. This comparison is really critical for an evaluation of the proposed method as currently a reader can not be sure if the methodological complexity of the proposed method (e.g., utilization of Deep Count Autoencoder) actually gives any benefits in comparison with a conventional "straightforward" approach.
At the same time, I completely agree with the authors that it is impossible to discuss all the aspects and questions within one manuscript, and that certain discussion and certain comparisons should appear later as separate manuscripts (both from the same group of authors and from independent groups - I'm completely sure that multiple groups will be interested in evaluation and routine application of the proposed method). Therefore, my overall recommendation is to accept the manuscript for publication in its present form.

Level of Interest

Please indicate how interesting you found the manuscript: Choose an item.

Quality of Written English

Please indicate the quality of language in the manuscript: Choose an item.

Declaration of Competing Interests

Please complete a declaration of competing interests, considering the following questions:

- Have you in the past five years received reimbursements, fees, funding, or salary from an organisation that may in any way gain or lose financially from the publication of this manuscript, either now or in the future?
- Do you hold any stocks or shares in an organisation that may in any way gain or lose financially from the publication of this manuscript, either now or in the future?
- Do you hold or are you currently applying for any patents relating to the content of the manuscript?
- Have you received reimbursements, fees, funding, or salary from an organization that holds or has applied for patents relating to the content of the manuscript?
- Do you have any other financial competing interests?
- Do you have any non-financial competing interests in relation to this paper?

If you can answer no to all of the above, write 'I declare that I have no competing interests' below. If your reply is yes to any, please give details below.

I declare that I have no competing interests.

I agree to the open peer review policy of the journal. I understand that my name will be included on my report to the authors and, if the manuscript is accepted for publication, my named report including any attachments I upload will be posted on the website along with the authors' responses. I agree for my report to be made available under an Open Access Creative Commons CC-BY license (http://creativecommons.org/licenses/by/4.0/). I understand that any comments which I do not wish to be included in my named report can be included as confidential comments to the editors, which will not be published.

Choose an item.

To further support our reviewers, we have joined with Publons, where you can gain additional credit to further highlight your hard work (see: https://publons.com/journal/530/gigascience). On publication of this paper, your review will be automatically added to Publons, you can then choose whether or not to claim your Publons credit. I understand this statement.

Yes Choose an item.
