# Supplementary material for: DrivAER: Identification of driving transcriptional programs in single-cell RNA sequencing data
Source: Gigascience. 2020 Dec 10;9(12):giaa122. doi: 10.1093/gigascience/giaa122 (PMC7727875; doi:10.1093/gigascience/giaa122)
Supplement: giaa122_Reviewer_4_Report_Original_Submission — Andrew McDavid -- 3/5/2020 Reviewed [file giaa122_reviewer_4_report_original_submission.pdf]

Reviewer Report

Title: DrivAER: Identification of driving transcriptional programs in single-cell RNA sequencing data

Version: Original Submission **Date:** 3/5/2020

Reviewer name: Andrew McDavid

Reviewer Comments to Author:

Simon, Yan and Zhao describe a method to infer biologically important
gene sets, from single-cell
RNA sequencing data. These gene sets are called "transcriptional
programs." The method is semi-supervised. It
requires external, a priori labels, or labels learned from a
preliminary analysis of the single cell RNAseq experiment in question. It
compares the 2-d embedding by training a neural network architecture
("Deep count autoencoder"; DCA, that Eraslan, Simon and colleagues introduced in 2019) with these semi-supervised
labels. Genes, that when run through the DCA yield
embeddings that are predictive of the semi-supervised labels are
assigned to a transcriptional program. They qualitatively compare their method to an existing method, and to an existing
gene set. Software is provided as a python module that is
intended to be compatible with scanpy.
The paper appears to propose a novel application of auto-encoders to
score gene sets in single cell RNAseq data, and addresses one, of many
areas of interest in single cell RNAseq analysis. 1) However, the methods do not provide adequate detail for an interested reader to understand the proposal. 2) The
benchmarking of the proposed approach is too
limited for users to judge if it provides benefits over
existing methods.
1) Important details of the method and its application are unclear,
including
a) Can only one gene set be examine at a time? If
multiple gene sets are at work, for instance cell cycle and interferon
response, how could a user identify these, or manage such confounding?
c) Only after a careful second reading was it clear that the goal of
the paper is to score existing gene sets rather than discover gene
sets de novo. This should be made more explicit.
d) Is there some
online or hot-start approach that is being exploited so that the DCA

doesn't have to be retrained from scratch each time a gene is added
or deleted from the proposed set?
e) Relating to d), what are the timings for the various analyses and approaches compared here. How much compute time is required to run DrivAER?
2) Systematic explorations of the sensitivity and specificity of their approach are not provided, including against simple alternatives to the proposed method. I do credit the comparison to VISION, but the problem of scoring gene sets is an old one.
a) At a minimum, the authors should consider including typical methods for gene set enrichment, ie, GSEA, hypergeometric tests or competitive tests developed for single cell RNAseq (Finak 2015) on genes that are differentially expressed according to the semi-supervision label that the Random Forest is using. This approach is currently dismissed in the introduction with the argument that "choosing the best parameters to identify differentially expressed genes across diverse scRNA-seq datasets is still an open challenge" but there are a lot of well-developed proposals that have attempted to solve this challenge. There is no reason to not pick one that is commonly used in practice and compare to it.
b) There are also factor analysis and related methods specifically for simultaneously inferring and testing gene sets in scRNAseq data such as f-scLVM (Buettner 2017) or zinbwave (Risso 2019), or pagoda2 (Fan 2016). The more comparisons the authors can make, the more useful of a contribution to the literature this paper will be.
c) The comparisons should be made quantitative, in terms of the ROC, or a related trade off for various methods. By cross-validating the sample into test and train before training the DCA, and then projecting the test into into the DCA calculating the RF accuracy on the test data, a standard error on the RF accuracy could be provided, and an approximate Wald test conducted. Other procedures may be possible here. The aspect that should be addressed is control of the false positive rate.
d) To assess sensitivity, simulation could be considered, or if the authors can show that they can control the false positive rate, they may argue that the number of positives returned at a given FPR is a proxy for the PPV of their method. Simulation of course also permits the specificity to be calculated.
e) How is the random forest accuracy (which I guess means MSE if a continuous label is present) measured? Out of bag?

Level of Interest

Please indicate how interesting you found the manuscript: Choose an item.

Quality of Written English

Please indicate the quality of language in the manuscript: Choose an item.

Declaration of Competing Interests

Please complete a declaration of competing interests, considering the following questions:

- Have you in the past five years received reimbursements, fees, funding, or salary from an organisation that may in any way gain or lose financially from the publication of this manuscript, either now or in the future?
- Do you hold any stocks or shares in an organisation that may in any way gain or lose financially from the publication of this manuscript, either now or in the future?
- Do you hold or are you currently applying for any patents relating to the content of the manuscript?
- Have you received reimbursements, fees, funding, or salary from an organization that holds or has applied for patents relating to the content of the manuscript?
- Do you have any other financial competing interests?
- Do you have any non-financial competing interests in relation to this paper?

If you can answer no to all of the above, write 'I declare that I have no competing interests' below. If your reply is yes to any, please give details below.

I declare I have no competing interests.

I agree to the open peer review policy of the journal. I understand that my name will be included on my report to the authors and, if the manuscript is accepted for publication, my named report including any attachments I upload will be posted on the website along with the authors' responses. I agree for my report to be made available under an Open Access Creative Commons CC-BY license (http://creativecommons.org/licenses/by/4.0/). I understand that any comments which I do not wish to be included in my named report can be included as confidential comments to the editors, which will not be published.

Choose an item.

To further support our reviewers, we have joined with Publons, where you can gain additional credit to further highlight your hard work (see: https://publons.com/journal/530/gigascience). On publication of this paper, your review will be automatically added to Publons, you can then choose whether or not to claim your Publons credit. I understand this statement.

Yes Choose an item.
