# Supplementary material for: DrivAER: Identification of driving transcriptional programs in single-cell RNA sequencing data
Source: Gigascience. 2020 Dec 10;9(12):giaa122. doi: 10.1093/gigascience/giaa122 (PMC7727875; doi:10.1093/gigascience/giaa122)
Supplement: giaa122_Reviewer_4_Report_Revision_1 — Andrew McDavid -- 6/5/2020 Reviewed [file giaa122_reviewer_4_report_revision_1.pdf]

Reviewer Report

Title: DrivAER: Identification of driving transcriptional programs in single-cell RNA sequencing data

Version: Revision 1 **Date:** 6/5/2020

Reviewer name: Andrew McDavid

Reviewer Comments to Author:

In this revision, the authors have substantially clarified their presentation. It is easier to understand the data input requirements, and expected results of the method. The authors have provided some comparison to a two existing methods using a simulation study, which has the potential to better place DrivAER method into context.
I do still yearn for discussion of the advantages the authors envision from applying their method, compared to classical gene set enrichment or hypergeometric tests on the differential expression coefficients. As I wrote in my initial critique, I am not convinced that because identifying "differentially expressed genes
across diverse scRNA-seq datasets is still an open challenge" as the authors say, means that these methods should be ignored. The lack of discussion or context about existing methods for geneset enrichment using differential expression is worrisome, given the very large body of literature and history behind them, and their simplicity. The authors should consider reading and discussing Subramanian 2005 (https://www.pnas.org/content/102/43/15545), Wu 2012 (https://www.ncbi.nlm.nih.gov/pmc/articles/PMC3458527/), and Goeman 2007 (10.1093/bioinformatics/btm051). Subramanian and the hypergeometric test are used incredibly frequently in applied scRNAseq work, even though I am not aware of a reference yet about their repurposing. Finak 2015 (https://genomebiology.biomedcentral.com/articles/10.1186/s13059-015-0844-5, full disclosure, I am a coauthor of this work) proposed a modification to the Wu 2012 method, that purports to be better suited for scRNAseq data. Deep auto-encoders are more complicated than any of the references above, and there is always a risk of complexity for its own sake.
This being said, I believe there is likely is some benefit to the DCA method. Particularly its the ability to visualize the low-dimensional embedding of the data along the gene set is quite different from methods that only use beta coefficients, and might uncover biology that would be hidden using all the genes. There are some methods that also attempt to identify latent variation that is aligned with gene sets, some which were mentioned in my initial review. Existing methods include f-scLVM (Buettner 2017), zinbwave (Risso
2019), Plier (Mao 2019, https://www.nature.com/articles/s41592-019-0456-1). The authors could comment if they believe DrivAER could be used in this fashion. The fact that DrivAER appears to be relatively time-efficient seems like it could be a real advantage.
I am heartened to see that the authors have expanded their comparison set to include Pagoda, and offer a new figure that shows the power vs signal size. However, I am left to wonder why the authors didn't choose to present this as an ROC-type curve showing the sensitivity vs specificity of their method vs

competitors? As it stands, it's not really possible to compare performance across the methods.
All said, this work has been improved in revision. Its impact would be even further improved by a better connection in its bibliography, discussion and potentially methodological comparison to the many existing methods for conducting gene set enrichment.

Level of Interest

Please indicate how interesting you found the manuscript: Choose an item.

Quality of Written English

Please indicate the quality of language in the manuscript: Choose an item.

Declaration of Competing Interests

Please complete a declaration of competing interests, considering the following questions:

- Have you in the past five years received reimbursements, fees, funding, or salary from an organisation that may in any way gain or lose financially from the publication of this manuscript, either now or in the future?
- Do you hold any stocks or shares in an organisation that may in any way gain or lose financially from the publication of this manuscript, either now or in the future?
- Do you hold or are you currently applying for any patents relating to the content of the manuscript?
- Have you received reimbursements, fees, funding, or salary from an organization that holds or has applied for patents relating to the content of the manuscript?
- Do you have any other financial competing interests?
- Do you have any non-financial competing interests in relation to this paper?

If you can answer no to all of the above, write 'I declare that I have no competing interests' below. If your reply is yes to any, please give details below.

I declare I have no competing interests.

I agree to the open peer review policy of the journal. I understand that my name will be included on my report to the authors and, if the manuscript is accepted for publication, my named report including any attachments I upload will be posted on the website along with the authors' responses. I agree for my report to be made available under an Open Access Creative Commons CC-BY license (http://creativecommons.org/licenses/by/4.0/). I understand that any comments which I do not wish to be included in my named report can be included as confidential comments to the editors, which will not be published.

Choose an item.

To further support our reviewers, we have joined with Publons, where you can gain additional credit to further highlight your hard work (see: https://publons.com/journal/530/gigascience). On publication of this paper, your review will be automatically added to Publons, you can then choose whether or not to claim your Publons credit. I understand this statement.

Yes Choose an item.
